# Chemical constituents from the aerial parts of *Musella lasiocarpa*

**DOI:** 10.1007/s13659-011-0007-7

**Published:** 2011-09-05

**Authors:** Liao-Bin Dong, Juan He, Xing-Yao Li, Xing-De Wu, Xu Deng, Gang Xu, Li-Yan Peng, Yu Zhao, Yan Li, Xun Gong, Qin-Shi Zhao

**Affiliations:** 1 State Key Laboratory of Phytochemistry and Plant Resources in West China, Kunming Institute of Botany, Chinese Academy of Sciences, Kunming, 650201 China; 2 Graduate University of Chinese Academy of Sciences, Beijing, 100039 China

**Keywords:** monotypic, diarylheptanoid, phenylphenalenone, acenaphtylene, sucrose ester, *Musella lasiocarpa*

## Abstract

Phytochemical investigation of the aerial parts of the monotypic plant, *Musella lasiocarpa*, led to the isolation of four rare bicyclic diarylheptanoids, musellarins B-E (**2–5**), two new phenylphenalenones, 2-methoxy-9-(3′,4′-dihydroxyphenyl)-1*H*phenalen-1-one (**9**), 2-methoxy-9-(3′-methoxy-4′-hydroxyphenyl)-1*H*-phenalen-1-one (**10**), a new acenaphtylene derivative, *trans*-(1*S*,2*S*)-3-(4′-methoxyphenyl)-acenaphthene-1,2-diol (**13**), and two new sucrose esters, 1,2′,3′,4′,6′-*O*-pentaacetyl-3-*O*-*trans*-*p*coumaroylsucrose (**16**), 1,2′,3′,4′,6′-*O*-pentaacetyl-3-*O*-*cis*-*p*-coumaroylsucrose (**17**), together with nine known compounds. In addition, (4*E*,6*E*)-1-(3′,4′-dihydroxyphenyl)-7-(4″-hydroxyphenyl)-hepta-4,6-dien-3-one (**15**) was isolated for the first time from a natural source. The structures of new compounds were elucidated by analysis of their spectroscopic data. Compounds **2, 6, 8–10, 12**, and **14** were cytotoxic toward several of the human tumor cell lines (HL-60, SMMC-7721, A-549, MCF-7, and SW480). Of these, the new compound **9** was the most potent one, with IC_50_ values of 5.8, 10.3, 6.3, 3.3, and 2.3 µM, respectively.

**Electronic Supplementary Material:**

Supplementary material is available for this article at 10.1007/s13659-011-0007-7 and is accessible for authorized users.

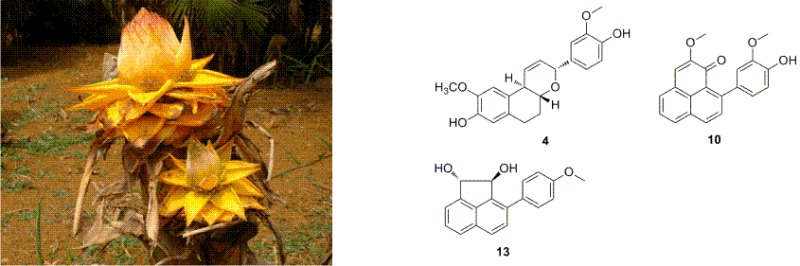

## Introduction

*Musella lasiocarpa* (Musaceae) is a monotypic species, which is distributed in the conifer-oak mixed forests at 1500-2500 m and endemic to the area from the middle to the west of Yunnan Province in China.[1] Due to its strongly yellow-colored spherical flowers, the plant was used as an ornamental in some Asian countries. Moreover, *M. lasiocarpa* has been used as a folk remedy for treatment of some gynaecological diseases, such as metrorrhagia and leucorrhoea, bleeding, enteritis, constipation, monkshood (*Aconitum* spp.) poisoning, drunkenness, etc.[1,2] Previous studies on this plant have resulted in the isolation of four phenylphenalenones,[3] the characteristic compounds of the family Musaceae, an amide,[4] a lactone,[4] and several other compounds.[5] In addition, several of the compounds exhibited antibacteria and cytotoxic activities.[3,4] In the course of our systematic search for bioactive compounds from the monotypic species endemic in China,[6] four rare bicyclic diarylheptanoids (**2–5**), two new phenylphenalenones (**9** and **10**), a new acenaphtylene derivative (**13**), and two new sucrose esters (**16** and **17**), together with nine known compounds were isolated from the title plant. Except for **13**, **16**, and **17**, the other compounds were evaluated for cytotoxicity against five human tumor cell lines (HL-60, SMMC-7721, A-549, MCF-7,

*To whom correspondence should be addressed. E-mail: qinshizhao@mail.kib.ac.cn.

Springer and SW480). Herein, we report the isolation, structural elucidation, and cytotoxicity of the compounds obtained in this investigation.

## Results and Discussion

Compound **2** was isolated as a white, amorphous powder. The molecular formula $C_{21}H_{22}O_5$ was established on the basis of HREIMS (*m/z* 354.1461 $[M]^+$, calcd for 354.1467). The IR spectrum showed the presence of hydroxyl (3424 $cm^{-1}$) and aromatic (1611 and 1512 $cm^{-1}$) functionalities. The $^1H$ NMR spectrum (Table 1) revealed signals of a 1,3,4-trisubstituted aromatic ring [$\delta_H$ 7.01 (1H, br s, H-2′), 6.80 (1H, d, $J$ = 8.0 Hz, H-5′), and 6.86 (1H, br d, $J$ = 8.0 Hz, H-6′)], a 1,2,4,5-tetrasubstituted aromatic ring [$\delta_H$ 6.54 (1H, s, H-7), 6.89 (1H, s, H-10)], two aromatic methoxy groups [$\delta_H$ 3.82 (3H, s), 3.84 (3H, s)], two olefinic protons [$\delta_H$ 6.28 (1H, ddd, $J$ = 10.2, 4.0, 2.0 Hz, H-1), 5.90 (1H, dt, $J$ = 10.2, 2.0 Hz, H-2)], two oxygenated methine protons [$\delta_H$ 5.05 (1H, br s, H-3), 4.14 (1H, m, H-4a)], and four methylene protons [$\delta_H$ 1.79 (1H, m, H-5$\alpha$), 2.03 (1H, m, H-5$\beta$), 2.55 (1H, m, H-6$\alpha$), and 2.85 (1H, m, H-6$\beta$)]. The above moieties were further confirmed by the $^{13}C$ NMR data (Table 2) and DEPT experiments. Correlations in the $^1H$–$^1H$ COSY and HSQC spectra revealed the presence of a $CH_2$(6)–$CH_2$(5)–CH(4a)–CH(10b) –CH(1)=CH(2)–CH(3) unit (Figure 1). The HMBC correlations from H-3 at $\delta_H$ 5.05 (1H, br s) to C-4a ($\delta_C$ 68.1, d), C-2′ ($\delta_C$ 112.1, d), and C-6′ ($\delta_C$ 121.3, d) suggested the presence of a tetrahydropyran unit bearing an aromatic ring connected to C-3. Furthermore, in the HMBC spectrum the correlations from H-7 at $\delta_H$ 6.54 (1H, s) to C-6 ($\delta_C$ 26.2, t), and H-10 at $\delta_H$ 6.89 (1H, s) to C-10b ($\delta_C$ 37.5, d) indicated the existence of a 1,2,3,4-tetrahydronaphthalene group. The two methoxy groups were located at C-9 and C-3′ as evidenced by the HMBC correlations of $OC\underline{H}_3$/C-9 and $OC\underline{H}_3$/C-3′, and the ROESY correlations of $OC\underline{H}_3$/H-10 and $OC\underline{H}_3$/H-2′.

The relative configuration of compound **2** was determined on the basis of a ROESY experiment (Figure 1). The ROESY correlations of H-4a/H-10b, H-4a/H-2′ and H-6′, H-4a/H-6$\alpha$, and H-10b/H-5$\alpha$, indicated that these protons were cofacial and assigned as $\alpha$-oriented.[7] In consequence, the ROESY cross-peak of H-3/H-5$\beta$ demonstrated that they were $\beta$-oriented. Thus, compound **2** was elucidated as *rel*-(3*S*,4a*R*,10b*R*)-3-(3′-methoxy-4′-hydroxyphenyl)-8-hydroxy-9-methoxy-4a,5,6,10b-tetrahydro-3*H*-naphtho[2,1-*b*]pyran, and named as musellarin B.

**Figure 1.** Selected 2D NMR correlations observed for **2**.

Compounds **3** and **4** had the same molecular weight [**3**, HREIMS *m/z* 354.1471 $[M]^+$, $C_{21}H_{22}O_5$; **4**, HREIMS *m/z* 354.1470 $[M]^+$, $C_{21}H_{22}O_5$] and their NMR spectroscopic data (Tables 1 and 2) indicated that their structures were closely related to **2**. Compared to **2**, one apparent change in **3** was the different position of the methoxy group at the 1,3,4-trisubstituted aromatic ring. The strong cross-peaks in the HMBC ($OC\underline{H}_3$-4′/C-4′ and H-6′/C-4′) and ROESY ($OC\underline{H}_3$-4′/H-5′) spectra of **3** indicated that the methoxy group was placed at C-4′. The difference between **4** and **2** was the relative configuration of H-4a, which was determined by the ROESY correlation of H-4a/H-3 in **4**. Therefore, compound **3** was identified as *rel*-(3*S*,4a*R*,10b*R*)-3-(3′-hydroxy-4′-methoxyphenyl)-8-hydroxy-9-methoxy-4a,5,6,10b-tetrahydro-3*H*-naphtho[2,1-*b*]pyran, and named as musellarin C, while the structure *rel*-(3*S*,4a*S*,10b*R*)-3-(3′-methoxy-4′-hydroxyphenyl)-8-hydroxy-9-methoxy-4a,5,6,10b-tetrahydro-3*H*-naphtho[2,1-*b*]pyran was proposed for musellarin D (**4**).

Compound **5**, a white, amorphous powder, gave an $[M]^+$ peak at *m/z* 324.1367 ($C_{20}H_{20}O_4$) in the HREIMS, 30 mass units less than that of **4**. According to the 1D NMR data (Tables 1 and 2), compound **5** was determined to be an analogue

**Table 1.** $^1H$ **NMR spectroscopic data of compounds 2–5**[a] **in** $CD_3OD$ (***J*** **in Hz).**

| Pos. | **2**[bc] | **3**[d] | **4**[c] | **5**[d] |
|---|---|---|---|---|
| 1 | 6.28, ddd (10.2, 4.0, 2.0) | 6.26, ddd (10.2, 4.0, 2.0) | 6.45, ddd (10.0, 6.4, 1.8) | 6.42, ddd (10.0, 6.0, 1.8) |
| 2 | 5.90, dt (10.2, 2.0) | 5.87, dt (10.2, 2.0) | 5.73, br d (10.0) | 5.74, dt (10.0, 1.8) |
| 3 | 5.05, br s | 5.05, d (1.5) | 5.13, d (1.9) | 5.11, br s |
| 4a | 4.14, m | 4.18, m | 4.23, dd (5.6, 3.2) | 4.22, dd (6.8, 3.8) |
| 5 | 1.79, m ($\alpha$); 2.03, m ($\beta$) | 1.84, m ($\alpha$); 2.05, m ($\beta$) | 1.90, m ($\alpha$); 2.07, m ($\beta$) | 1.87, m($\alpha$); 2.04, m ($\beta$) |
| 6 | 2.55, m ($\alpha$); 2.85, m ($\beta$) | 2.58, m ($\alpha$); 2.84, m ($\beta$) | 2.49, ddd (16.6, 5.6, 2.4, $\alpha$) | 2.43, ddd (16.0, 5.5, 3.0, $\alpha$) |
| | | | 2.96, ddd (16.6, 12.8, 4.8, $\beta$) | 2.93, ddd (16.0, 12.5, 4.5, $\beta$) |
| 7 | 6.54, s | 6.52, s | 6.56, s | 6.54, s |
| 10 | 6.89, s | 6.83, s | 6.87, s | 6.86, s |
| 10b | 3.36, br s | 3.41, br s | 3.27, br s | 3.25, br s |
| 2′ | 7.01, br s | 6.89, overlap | 6.52, d (1.2) | 6.96, d (8.5) |
| 3′ | | | | 6.64, d (8.5) |
| 5′ | 6.80, d (8.0) | 6.90, overlap | 6.64, overlap | 6.64, d (8.5) |
| 6′ | 6.86, br d (8.0) | 6.85, dd (8.0, 1.5) | 6.63, overlap | 6.96, d (8.5) |
| 9-$OCH_3$ | 3.82, s | 3.84, s | 3.82, s | 3.83, s |
| 3′-$OCH_3$ | 3.84, s | | 3.50, s | |
| 4′-$OCH_3$ | | 3.85, s | | |

[a]Assignments are based on 1D and 2D NMR experiments. [b] in $CD_3COCD_3$. [c] 400 MHz. [d] 500 MHz of **4** in which the methoxy group at C-3′ was replaced by a hydrogen. Detailed analysis of 2D NMR spectra indicated that the other parts of **5** were the same as those of **4**. Thus, compound **5** was determined as *rel*-(3*S*,4a*S*,10b*R*)-3-(4′-hydroxyphenyl)-8-hydroxy-9-methoxy-4a,5,6,10b-tetrahydro-3*H*-naphtho[2,1-*b*]pyran, and named as musellarin E.

The HREIMS of compound **9** showed a protonated molecular ion at *m/z* 318.0883 $[M]^+$, corresponding to molecular formula $C_{20}H_{14}O_4$ with 14 degrees of unsaturation. Analysis of the $^1H$ NMR spectrum (Table 3) indicated the existence of a 2,4- or 2,9-substituted phenalen-1-one, a 1,3,4-trisubstituted benzene, and a methoxy moieties, which suggested that **9** was a phenylphenalen-1-one derivative substituted at C-4 or C-9 with a 1,3,4-trisubstituted aromatic ring. Moreover, in the mass spectrum, **9** exhibited higher intensity of the $[M - H]^+$ ion at *m/z* 317 than that of the $[M]^+$ ion at *m/z* 318 (Supporting Information), supporting that the side chain was located at C-9.[8] This conclusion was also supported by the cross peak (H-3/H-4) in ROESY spectrum. The correlations of -OC$\underline{H}_3$/C-2 in HMBC spectrum and -OC$\underline{H}_3$/H-3 in ROESY spectrum suggested the methoxy group was connected to C-2. Thus, comp ound **9** was established as 2-methoxy-9-(3′,4′-dihydroxyphenyl)-1*H*-phenalen-1-one.

**Table 2.** $^{13}$**C NMR spectroscopic data of compounds 2–5**[a] **in** $CD_3OD$.

| Pos. | 2[bc] | 3[c] | 4[c] | 5[d] |
|---|---|---|---|---|
| 1 | 129.8, CH | 130.2, CH | 128.3, CH | 128.5, CH |
| 2 | 128.5, CH | 128.4, CH | 131.6, CH | 131.5, CH |
| 3 | 73.0, CH | 73.7, CH | 78.4, CH | 78.4, CH |
| 4a | 68.1, CH | 69.6, CH | 72.0, CH | 72.5, CH |
| 5 | 27.2, CH2 | 27.3, CH2 | 29.3, CH2 | 29.6, CH2 |
| 6 | 26.2, CH2 | 26.8, CH2 | 24.8, CH2 | 24.8, CH2 |
| 6a | 129.4, C | 129.8, C | 129.7, C | 129.9, C |
| 7 | 115.4, CH | 115.9, CH | 116.0, CH | 116.1, CH |
| 8 | 145.5, C | 145.6, C | 145.4, C | 145.3, C |
| 9 | 147.0, C | 147.8, C | 147.6, C | 147.6, C |
| 10 | 112.4, CH | 112.7, CH | 112.6, CH | 112.5, C |
| 10a | 129.4, C | 129.7, C | 130.3, C | 129.9, C |
| 10b | 37.5, CH | 38.1, CH | 37.6, CH | 37.5, CH |
| 1′ | 133.8, C | 135.1, C | 134.8, C | 134.0, C |
| 2′ | 112.1, CH | 116.1, CH | 111.4, CH | 129.5, CH |
| 3′ | 148.1, C | 147.5, C | 148.9, C | 116.0, CH |
| 4′ | 147.0, C | 148.8, C | 147.1, C | 158.1, C |
| 5′ | 115.3, CH | 112.4, CH | 115.5, CH | 116.0, CH |
| 6′ | 121.3, CH | 120.5, CH | 120.7, CH | 129.5, CH |
| 9-OCH3 | 56.3, CH3 | 56.5, CH3 | 56.5, CH3 | 56.6, CH3 |
| 3′-OCH3 | 56.2, CH3 | | 55.9, CH3 | |
| 4′-OCH3 | | 56.4, CH3 | | |

[a]Assignments are based on 1D and 2D NMR experiments. [b]in $CD_3COCD_3$. [c]100 MHz. [d]125 MHz.

**Table 3.** $^{13}$**C NMR and** $^1$**H NMR spectroscopic data of compounds 9**[a]**, 10**[a]**, 13**[a] **in** $CD_3OD$.

| | 9 | | 10 | | 13 | | |
|---|---|---|---|---|---|---|---|
| Pos. | $\delta_C$[b] | $\delta_H$ (*J* in Hz)[d] | $\delta_C$[c] | $\delta_H$ (*J* in Hz)[d] | Pos. | $\delta_C$[c] | $\delta_H$ (*J* in Hz)[e] |
| 1 | 181.8, C | | 181.8, C | | 1 | 84.0, CH | 5.30, br s |
| 2 | 154.6, C | | 154.6, C | | 2 | 83.1, CH | 5.48, br s |
| 3 | 113.4, CH | 7.14, s | 113.6, CH | 7.12, s | 2a | 139.5, C | |
| 3a | 126.4, C | | 126.5, C | | 3 | 136.9, C | |
| 4 | 131.5, CH | 7.82, d (7.2) | 131.5, CH | 7.79, d (7.2) | 4 | 131.1, CH | 7.65, d (8.5) |
| 5 | 127.9, CH | 7.61, t (7.5) | 127.9, CH | 7.60, overlap | 5 | 126.8, CH | 7.86, d (8.5) |
| 6 | 130.8, CH | 7.98, d (8.4) | 130.7, CH | 7.97, d (8.4) | 5a | 131.6, C | |
| 6a | 132.6, C | | 132.7, C | | 6 | 125.7, CH | 7.80, dd (6.8, 1.8) |
| 7 | 136.0, CH | 8.24, d (8.4) | 136.1, CH | 8.23, d (7.8) | 7 | 122.4, CH | 7.56, overlap |
| 8 | 133.0, CH | 7.59, d (8.4) | 132.9, CH | 7.59, overlap | 8 | 128.9, CH | 7.57, overlap |
| 9 | 150.5, C | | 150.2, C | | 8a | 144.0, C | |
| 9a | 126.4, C | | 126.5, C | | 8b | 138.8, C | |
| 9b | 129.6, C | | 129.6, C | | 1′ | 133.8, C | |
| 1′ | 135.8, C | | 135.8, C | | 2′/6′ | 131.1, CH | 7.74, d (8.5) |
| 2′ | 116.7, CH | 6.80, d (1.8) | 113.5, CH | 6.93, d (1.8) | 3′/5′ | 115.0, CH | 7.04, d (8.5) |
| 3′ | 146.2, C | | 148.9, C | | 4′ | 160.6, C | |
| 4′ | 146.2, C | | 147.4, C | | 4′-OCH3 | 55.8, CH3 | 3.86, s |
| 5′ | 116.3, CH | 6.84, d (7.8) | 116.3, CH | 6.87, d (7.8) | | | |
| 6′ | 120.9, CH | 6.69, dd (7.8, 1.8) | 122.1, CH | 6.80, dd (7.8, 1.8) | | | |
| 2-OCH3 | 56.1, CH3 | 3.88, s | 56.1, CH3 | 3.88, s | | | |
| 3′-OCH3 | | | 56.6, CH3 | 3.84, s | | | |

[a]Assignments are based on 1D and 2D NMR experiments. [b]150 MHz. [c]125 MHz. [d]600 MHz. [e]500 MHz.

Compound **10** had an $[M]^+$ peak at *m/z* 332.1046 ($C_{21}H_{16}O_4$), 14 mass units higher than that of **9**. Analysis of its 1D NMR spectra (Table 3) indicated that **10** was a structural analogue of **9**, the only difference in **10** being the replacement of the hydroxyl group at C-3′ with a methoxy group. This was determined by the correlation of –OC$\underline{H}_3$/H-2′ in ROESY spectrum. Therefore, compound **10** was established as 2-methoxy-9-(3′-methoxy-4′-hydroxyphenyl)-1*H*-phenalen-1-one.

Compound **13** had the molecular formula $C_{19}H_{16}O_3$ based on HRESIMS ($[M + Na]^+$ *m/z* 315.0990; calcd for $C_{19}H_{16}O_3Na$, 315.0997). The $^1H$ NMR spectrum (Table 3) exhibited nine aromatic protons corresponding to three systems (AMX, AB, and $A_2B_2$ system) of three benzene rings. The $^{13}C$ NMR spectrum showed 19 signals, including 16 aromatic carbons, a methoxy group at $\delta_C$ 55.8, and two oxygenated carbons at $\delta_C$ 84.0 and 83.1. The above data were very similar to those of 3-phenyl-1,2-dihydroacenaphtylen-1,2-diol.[9] Analysis of the 1D NMR spectra of the two compounds revealed that marked differences were the *trans* configuration of H-1 and H-2 as well as one more methoxy group located at C-4′ in **13**. These were confirmed by the small coupling constant ($J = 0$ Hz) of H-1 and H-2,[9,10] and the HMBC correlation of $\delta_H$ 3.86 (s, OC$\underline{H}_3$-4′) with $\delta_C$ 160.6 (s, C-4′). Due to the small amounts obtained (1.0 mg), the absolute configuration of **13** could not be determined through the method of exiton-coupled circular dichroism (ECCD).[11] However, the specific rotation ($[\alpha]^{20}_D$ –22.7) value of **13** was similar to those of *trans*-(1*S*,2*S*)-acenaphthene-1,2-diol ($[\alpha]^{20}_D$ –24.1)[11] and opposite to those of

*trans*-(1*R*,2*R*)-acenaphthene-1,2-diol ($[\alpha]_D^{20}$ +33.2),[12] suggesting that the absolute configuration of **13** should be (1*S*,2*S*). Thus, compound **13** was deduced as *trans*-(1*S*,2*S*)-3-(4′-methoxyphenyl)-acenaphthene-1,2-diol.

**Table 4. $^{13}$C NMR and $^1$H NMR spectroscopic data of compounds 16$^a$, 17$^a$ in $CD_3OD$.**

| Pos. | 16 $\delta_C$[b] | 16 $\delta_H$ (*J* in Hz)[c] | 17 $\delta_C$[b] | 17 $\delta_H$ (*J* in Hz)[c] |
|---|---|---|---|---|
| 1 | 66.3, CH₂ | 4.23, d (11.6); 4.11, d (11.6) | 66.0, CH₂ | 4.17, d (11.4); 4.11, d (11.4) |
| 2 | 103.8, C | | 103.8, C | |
| 3 | 79.8, CH | 5.39, d (8.1) | 79.2, CH | 5.39, d (8.1) |
| 4 | 73.8, CH | 4.32, t (8.1) | 73.3, CH | 4.26, t (8.1) |
| 5 | 84.5, CH | 3.94, m | 84.3, CH | 3.90, m |
| 6 | 63.3, CH₂ | 3.79, overlap | 63.2, CH₂ | 3.77, overlap |
| 1′ | 90.5, CH | 5.69, d (3.6) | 90.5, CH | 5.66, d (3.6) |
| 2′ | 71.5, CH | 4.87, overlap | 71.6, CH | 4.87, overlap |
| 3′ | 71.2, CH | 5.39, t (9.8) | 71.3, CH | 5.35, t (9.8) |
| 4′ | 69.9, CH | 4.99, t (9.8) | 69.8, CH | 5.00, t (9.8) |
| 5′ | 69.7, CH | 4.35, m | 69.6, CH | 4.17, overlap |
| 6′ | 63.5, CH₂ | 4.18, overlap | 63.3, CH₂ | 4.17, overlap; 4.07, overlap |
| 1″ | 127.1, C | | 127.4, C | |
| 2″/6″ | 131.6, CH | 7.54, d (8.4) | 134.0, CH | 7.68, d (8.4) |
| 3″/5″ | 116.8, CH | 6.20, d (8.4) | 115.9, CH | 6.78, d (8.4) |
| 4″ | 161.5, C | | 160.4, CH | |
| 7″ | 147.9, CH | 7.75, d (16.0) | 146.7, C | 7.00, d (12.8) |
| 8″ | 114.2, CH | 6.44, d (16.0) | 115.4, CH | 5.90, d (12.8) |
| 9″ | 168.1, C | | 168.4, C | |
| OAc-1 | 172.0, C; 20.6, CH₃ | 2.10, s | 172.1, C; 20.6, CH₃ | 2.09, s |
| OAc-2′ | 171.8 C; 20.7, CH₃ | 2.04, s | 171.8, C 20.7, CH₃ | 2.03, s |
| OAc-3′ | 171.5, C; 20.5, CH₃ | 1.94, s | 171.6, C 20.6, CH₃ | 1.96, s |
| OAc-4′ | 171.3, C; 20.4, CH₃ | 1.84, s | 171.3, C 20.7, CH₃ | 2.01, s |
| OAc-6′ | 172.4, C; 20.7, CH₃ | 2.06, s | 172.4, C 20.6, CH₃ | 2.06, s |

[a]Assignments are based on 1D and 2D NMR experiments. [b]100 MHz. [c]400 MHz.

Compound **16** was obtained as a colorless, amorphous powder. Its molecular formula was determined to be $C_{31}H_{38}O_{18}$ by HRESIMS. The UV and IR spectra showed absorption bands for hydroxyl, $\alpha,\beta$-unsaturated carbonyl ester, and aromatic ring functionalities. The $^1$H NMR (Table 4) spectrum revealed that **16** possessed a *trans*-*p*-coumaroyl unit [$\delta_H$ 7.54 (2H, d, $J$ = 8.4 Hz, H-2″/6″), 6.20 (2H, d, $J$ = 8.4 Hz, H-3″/5″), 6.44 (1H, d, $J$ = 16.0 Hz, H-7″), and 7.75 (1H, d, $J$ = 16.0 Hz, H-8″)], 14 oxygenated protons ($\delta_H$ 3.79–5.69), and five alcoholic acetyl groups ($\delta_H$ 1.84–2.10). In the $^{13}$C NMR spectrum, the signals of the anomeric carbons [$\delta_C$ 103.8 (s, C-2), and 90.5 (d, C-1′)] indicated that the disaccharide moiety was sucrose.[13] Therefore, **16** was determined as a penta-acetylated derivative of *trans*-*p*-coumaroylsucrose. In the HMBC spectrum, the correlation networks of H-3/C-9″ ($\delta_C$ 168.1), H-1/OAc-1 ($\delta_C$ 172.0), H-2′/OAc-2′ ($\delta_C$ 171.8), H-3′/OAc-3′ ($\delta_C$ 171.5), H-4′/OAc-4′ ($\delta_C$ 171.3), and H-6′/OAc-6′ ($\delta_C$ 172.4) suggested the *trans*-*p*-coumaroyl moiety was linked to C-3 and the five alcoholic acetyl groups were located at C-1, C-2′, C-3′, C-4′, and C-6′, respectively. Moreover, alkaline hydrolysis of **16** with 0.5% NaOH in MeOH yielded sucrose (Experimental Section). Accordingly, compound **16** was assigned as 1,2′,3′,4′,6′-*O*-pentaacetyl-3-*O*-*trans*-*p*-coumaroylsucrose.

Compound **17**, a colorless, amorphous powder, gave a molecular formula of $C_{31}H_{38}O_{18}$, as determined on the basis of an HRESIMS ion at *m/z* 697.1980 $[M - H]^-$ (calcd for $C_{31}H_{37}O_{18}$, 697.1979). Its $^1$H NMR spectrum (Table 4) was similar to those of **16**, except for the existence of a *cis*-*p*-coumaroyl unit [$\delta_H$ 7.68 (2H, d, $J$ = 8.4 Hz, H-2″/6″), 6.78 (2H, d, $J$ = 8.4 Hz, H-3″/5″), 5.90 (1H, d, $J$ = 12.8 Hz, H-7″), and 7.00 (1H, d, $J$ = 12.8 Hz, H-8″)] replacing the *trans*-*p*-coumaroyl moiety of **16**. Therefore, the structure of **17** was characterized as 1,2′,3′,4′,6′-*O*-pentaacetyl-3-*O*-*cis*-*p*-coumaroylsucrose.

Hitherto, except for musellarins B–E (**2–5**), only two bicyclic diarylheptanoids were isolated from the natural kingdom, whose names were *rel*-(3*S*,4a*R*,10b*R*)-3-(4′-hydroxyphenyl)-8-hydroxy-9-methoxy-4a,5,6,10b-tetrahydro-3*H*-naphtho[2,1*b*] pyran (**1**, musellarin **A**),[7] and 3-(4′-hydroxyphenyl)-4a,5,6,10b-tetrahydro-1*H*-naphtho[2,1-*b*]pyran-1-one.[14] In addition, compound **13** was the second acenaphtylene derivative isolated from plants.

The known compounds were identified by comparison of their spectroscopic data with published values, as *rel*-(3*S*,4a*R*,10b*R*)-3-(4′-hydroxyphenyl)-8-hydroxy-9-methoxy-4a,5,6,10b-tetrahydro-3*H*-naphtho[2,1-*b*]pyran (**1**, musellarin **A**),[7] 2-hydroxy-9-(4′-hydroxyphenyl)-1*H*-phenalen-1-one (**6**, hydroxyanigorufone),[15] 2-methoxy-9-(4′-hydroxyphenyl)-1*H*-phenalen-1-one (**7**),[8] 2-hydroxy-9-(3′,4′-dihydroxyphenyl)-1*H*-phenalen-1-one (**8**, dihydroxyanigorufone),[15] 2-hydroxy-4-(3′,4′-dihydroxyphenyl)-1*H*-phenalen-1-one (**11**),[16] 2-hydroxy-4-(4′-methoxyphenyl)-1*H*-phenalen-1-one (**12**),[17] (4*E*,6*E*)-1,7-bis(4-hydroxyphenyl)-hepta-4,6-dien-3-one (**14**),[18] (4*E*,6*E*)-1-(3′,4′-dihydroxyphenyl)-7-(4″-hydroxyphenyl)-hepta-4,6-dien-

**Table 5. Cytotoxicity of 2, 6, 8–10, 12, and 14 against tumor cell lines$^a$ with $IC_{50}$ ($\mu$M).**

| Compound | HL-60 | SMMC-7721 | A-549 | MCF-7 | SW480 |
|---|---|---|---|---|---|
| **2** | 21.3 | 26.7 | 25.1 | > 40 | > 40 |
| **6** | 18.2 | 39.4 | 23.9 | > 40 | > 40 |
| **8** | 8.8 | >40 | > 40 | 27.3 | 28.4 |
| **9** | 5.8 | 10.3 | 6.3 | 3.3 | 2.3 |
| **10** | 12.1 | 21.3 | > 40 | 22.9 | 19.9 |
| **12** | > 40 | > 40 | > 40 | 33.1 | 37.5 |
| **14** | 6.4 | > 40 | 35.2 | > 40 | > 40 |
| cisplatin[b] | 1.0 | 17.0 | 16.0 | 17.1 | 19.1 |

[a]Cell lines: HL-60 acute leukemia; SMMC-7721 liver cancer; A-549 lung cancer; MCF-7 breast cancer; SW480 colon cancer. [b]positive control.

3-one (**15**),[19] and 2-(4′-hydroxyphenyl)-1,8-naphthalic anhydride.[20]

Previously, a number of phenylphenalenones with moderate cytotoxic effects against P388 murine leukemia cell line were reported from *Haemodorum simplex*.[21] In this study, except for **13**, **16**, and **17**, the other compounds were evaluated for cytotoxicity against five human tumor cell lines (HL-60, SMMC-7721, A-549, MCF-7, and SW480) using the MTT method.[22] Cisplation was used as the positive control. Of these compounds, **2**, **6**, **8–10**, **12**, and **14** were found to be active principles, and their cytotoxic activities were summarized in Table 5. The new compound **9** was the most cytotoxic against all of the five cell lines, with $IC_{50}$ values of 5.8, 10.3, 6.3, 3.3, and 2.3 $\mu$M, respectively.

## Experimental Section

**General Experimental Procedures**. Optical rotations were measured on a JASCO-20C digital polarimeter. IR spectra were obtained on a Tensor 27 spectrometer with KBr pellets. UV spectra were recorded using a Shimadzu UV-2401A spectrophotometer. 1D and 2D NMR spectra were performed on a Bruker AM-400, DRX-500 or AV III-600 spectrometers with TMS as an internal standard. Mass spectra were obtained on a VG Auto Spec-3000 or API-Qstar-Pulsar instrument. For sucrose, the ESIMS was taken on a Bruker Esquire HCT 3D ion trap mass spectrometer (ESI mode). Semipreparative HPLC was performed on an Agilent 1100 liquid chromatography with a Zorbax SB-C18 (9.4 mm × 25 cm) column. Column chromatography (CC) was performed using silica gel (200–300 mesh, Qingdao Marine Chemical Co. Ltd., Qingdao, People's Republic of China), MCI gel (75–150 $\mu$m; Mitsubishi Chemical Corporation, Japan), and Sephadex LH-20 (Amersham Pharmacia Biotech, Sweden).

**Plant Materials.** Plants of *M. lasiocarpa* were collected in the Kunming Botany Garden, Kunming, Yunnan Province, China, in September 2009, and were identified by one of the authors (Xun Gong). A voucher specimen (200909M) was deposited at the State Key Laboratory of Phytochemistry and Plant Resources in West China, Kunming Institute of Botany, Chinese Academy of Sciences.

**Extraction and Isolation.** The air-dried and powdered aerial parts of *M. lasiocarpa* (3.0 kg) were extracted with MeOH (4 × 10 L), each for 48 h, at room temperature and, concentrated in vacuo to give a crude extract. The extract was partitioned between $H_2O$ and EtOAc. The EtOAc portion (73.8 g) was decolorized on MCI gel (eluted with 90% MeOH) and then was chromatographed on MPLC (MCI gel) eluting with a gradient of MeOH–$H_2O$ (3:7, 6:4, 9:1, and 1:0) to afford four fractions (F01–F04). Fraction F03 (23.5 g) was fractionated by MPLC (MCI gel) again, eluted with MeOH-$H_2O$ (from 40% to 100%) to provide subfractions (F0301–F0305). Subfraction F0301 (800 mg) was chromatographed over silica gel CC, using $CHCl_3$-MeOH (20:1) as solvent, and then purified over Sephadex LH-20 eluted with MeOH, then by semipreparative HPLC (29% MeCN–$H_2O$) to give **16** ($t_R$ 21.8 min, 12 mg) and **17** ($t_R$ 24.7 min, 5 mg). Subfraction F0302 (1.0 g) was chromatographed over Sephadex LH-20 eluting with MeOH, and then purified repeatedly over silica gel CC, then by semipreparative HPLC (52% MeOH–$H_2O$) to give **15** ($t_R$ 20.4 min, 3 mg). Subfraction F0303 (1.84 g) was further chromatographed on silica gel CC, eluted with a gradient of $CHCl_3$-MeOH (150:1→0:1) to afford six subfractions F030301–F030306. Subfraction F030301 (94 mg) was purified on Sephadex LH-20 eluting with MeOH, and then chromatographed by semipreparative HPLC (52% MeOH–$H_2O$) to furnish **2** ($t_R$ 13.8 min, 3 mg), **3** ($t_R$ 17.1 min, 2 mg), and **4** ($t_R$ 22.4 min, 2 mg). Subfraction F030302 (136 mg) was chromatographed repeatedly over silica gel CC eluted with petroleum ether-acetone (10:1) to afford 2-(4′-hydroxyphenyl)-1,8-naphthalic anhydride (18 mg). Subfraction F030303 (240 mg) was chromatographed over a Sephadex LH-20 column, using MeOH as solvent, and then purified by semipreparative HPLC (55% MeOH–$H_2O$) to yield **13** ($t_R$ 10.8 min, 1 mg). Another peak with a retention time of 16.0 min was collected and further purified by preparative TLC eluted with petroleum ether-EtOAc (6:4) to furnish **1** (18 mg) and **5** (2 mg). Compound **8** (5 mg) and **11** (3 mg) were isolated from subfraction F030304 (110 mg) by preparative TLC, using toluene–EtOAc–formic acid (8:2:1) as solvent. Subfraction F030305 (75 mg) was purified by semipreparative HPLC (52% MeOH–$H_2O$) to yield **14** ($t_R$ 18.0 min, 15 mg). Subfraction F030306 (180 mg) was submitted to repeated silica gel CC, and then chromatographed by semipreparative HPLC (59% MeOH–$H_2O$) to afford **9** ($t_R$ 15.1 min, 5 mg). Subfraction F0304 (1.95 g) was subjected to passage over a silica gel column, eluted with a gradient of $CHCl_3$-MeOH (150:1→0:1) to afford five fractions F030401–F030405. Subfraction F030402 (140 mg) was purified by semipreparative HPLC (54% MeOH–$H_2O$) to give **10** ($t_R$ 26.2 min, 2 mg). Subfraction F030403 (145 mg) was chromatographed by semipreparative HPLC (60% MeOH–$H_2O$) to yield **6** ($t_R$ 13.5 min, 50 mg). Subfraction F030404 was separated by a silica gel column, using petroleum ether-acetone (8:2) as solvent system, then purified by Sephadex LH-20 eluted with MeOH to afford **7** (2 mg) and **12** (4 mg).

**Musellarin B (2):** white, amorphous powder; $[\alpha]_D^{20}$ − 223.3 (*c* 0.17, MeOH); UV (MeOH) $\lambda_{max}$ (log $\varepsilon$) 284 (3.63), 205 (4.51) nm; IR (KBr) $\nu_{max}$ 3424, 2931, 1611, 1512, 1450, 1272, 1113, 777 $cm^{-1}$; $^1H$ and $^{13}C$ NMR data, see Tables 1 and 2; positive EIMS *m/z* 354 $[M]^+$; positive HREIMS *m/z* 354.1461 $[M]^+$ (calcd for $C_{21}H_{22}O_5$, 354.1467).

**Musellarin C (3):** white, amorphous powder; $[\alpha]_D^{20}$ − 176.8 (*c* 0.30, MeOH); UV (MeOH) $\lambda_{max}$ (log $\varepsilon$) 284 (3.53), 206 (4.42) nm; IR (KBr) $\nu_{max}$ 3425, 2929, 1598, 1510, 1441, 1384, 1273, 1126, 790 $cm^{-1}$; $^1H$ and $^{13}C$ NMR data, see Tables 1 and 2; positive EIMS *m/z* 354 $[M]^+$; positive HREIMS *m/z* 354.1471 $[M]^+$ (calcd for $C_{21}H_{22}O_5$, 354.1467).

**Musellarin D (4):** white, amorphous powder; $[\alpha]_D^{20}$ − 41.9 (*c* 0.29, MeOH); UV (MeOH) $\lambda_{max}$ (log $\varepsilon$) 284 (3.26), 205 (4.21) nm; IR (KBr) $\nu_{max}$ 3431, 2929, 1629, 1514, 1451, 1277, 1114, 778 $cm^{-1}$; $^1H$ and $^{13}C$ NMR data, see Tables 1 and 2; positive EIMS *m/z* 354 $[M]^+$; positive HREIMS *m/z* 354.1470 $[M]^+$ (calcd for $C_{21}H_{22}O_5$, 354.1467).

**Musellarin E (5):** white, amorphous powder; $[\alpha]_D^{20}$ − 111.5 (*c* 0.19, MeOH); UV (MeOH) $\lambda_{max}$ (log $\varepsilon$) 284 (3.31), 225 (3.84), 205 (4.23) nm; IR (KBr) $\nu_{max}$ 3423, 2927, 1614, 1514, 1446, 1256, 1115, 834 $cm^{-1}$; $^1H$ and $^{13}C$ NMR data, see Tables 1 and 2; positive EIMS *m/z* 324 $[M]^+$; positive HREIMS *m/z* 324.1367 $[M]^+$ (calcd for $C_{20}H_{20}O_4$, 324.1362).

**2-Methoxy-9-(3′,4′-dihydroxyphenyl)-1*H*-phenalen-1-one (9):** red powder; UV (MeOH) $\lambda_{max}$ ( log $\varepsilon$) 412 (3.25), 366 (3.27), 268 (3.63), 263 (3.63), 205 (4.02) nm; IR (KBr) $\nu_{max}$ 3430, 1627, 1276 $cm^{-1}$; $^1H$ and $^{13}C$ NMR data, see Table 3; positive EIMS *m/z* 318 $[M]^+$; positive HREIMS *m/z* 318.0883 $[M]^+$ (calcd for $C_{20}H_{14}O_4$, 318.0892).

**2-Methoxy-9-(3′-methoxy-4′-hydroxyphenyl)-1*H*-phenalen-1-one (10):** red powder; UV (MeOH) $\lambda_{max}$ ( log $\varepsilon$) 412 (2.87), 365 (2.93), 262 (3.36), 217 (3.55) nm; IR (KBr) $\nu_{max}$ 3424, 1724, 1629, 1276 $cm^{-1}$; $^1H$ and $^{13}C$ NMR data, see Table 3; positive EIMS *m/z* 332 $[M]^+$; positive HREIMS *m/z* 332.1046 $[M]^+$ (calcd for $C_{21}H_{16}O_4$, 332.1049).

***trans*-(1*S*,2*S*)-3-(4′-Methoxyphenyl)-acenaphthene-1,2-diol (13):** colorless, amorphous powder; $[\alpha]_D^{20}$ − 22.7 (*c* 0.10, MeOH); UV (MeOH) $\lambda_{max}$ (log $\varepsilon$) 293 (3.37), 265 (3.78), 222 (3.79) nm; IR (KBr) $\nu_{max}$ 3431, 2922, 1630, 1460, 1249, 1033 $cm^{-1}$; $^1H$ and $^{13}C$ NMR data, see Table 3; positive ESIMS *m/z* 315 $[M + Na]^+$; positive HRESIMS *m/z* 315.0990 $[M + Na]^+$ (calcd for $C_{19}H_{16}O_3Na$, 315.0997).

**1,2′,3′,4′,6′-*O*-Pentaacetyl-3-*O*-*trans*-*p*-coumaroylsucrose (16):** colorless, amorphous powder; $[\alpha]_D^{20}$ + 42.5 (*c* 0.25, MeOH); UV (MeOH) $\lambda_{max}$ (log $\varepsilon$) 316 (3.73), 229 (3.41), 211 (3.38) nm; IR (KBr) $\nu_{max}$ 3440, 1723, 1630, 1244, 1050 $cm^{-1}$; $^1H$ and $^{13}C$ NMR data, see Table 4; negative ESIMS *m/z* 697 $[M - H]^-$; negative HRESIMS *m/z* 697.1960 $[M - H]^-$ (calcd for $C_{31}H_{37}O_{18}$, 697.1979).

**1,2′,3′,4′,6′-*O*-Pentaacetyl-3-*O*-*cis*-*p*-coumaroylsucrose (17):** colorless, amorphous powder; $[\alpha]_D^{20}$ − 15.3 (*c* 0.10, MeOH); UV (MeOH) $\lambda_{max}$ (log $\varepsilon$) 315 (3.59), 211 (3.39) nm; IR (KBr) $\nu_{max}$ 3433, 1751, 1630, 1236, 1046 $cm^{-1}$; $^1H$ and $^{13}C$ NMR data, see Table 4; negative ESIMS *m/z* 697 $[M - H]^-$; negative HRESIMS *m/z* 697.1980 $[M - H]^-$ (calcd for $C_{31}H_{37}O_{18}$, 697.1979).

**Alkaline Hydrolysis of 16.** A mixture of **16** (5.0 mg), 0.5% NaOH (0.5 ml), and MeOH (3 ml) was stirred at room temperature for 6 h. The reaction mixture was neutralized with 1 N HCl and extracted with $CHCl_3$ (3 × 10 ml). The aqueous layer was evaporated to dryness. The dry powder was chromatographyed over silica gel CC, eluted with $CHCl_3$-MeOH-$H_2O$ (35:25:2), to furnish sucrose (1.5 mg). Sucrose: $[\alpha]_D^{20}$ + 38.3 (*c* 0.10, $H_2O$); negative ESIMS *m/z* 377 $[M + Cl]^-$.

**Cytotoxicity Assay.** Cytotoxicity of selected compounds against HL-60, SMMC-7721, A-549, MCF-7, and SW480 cell lines was assessed using the MTT method.[22] Cells were plated in 96-well plates 12 h before treatment and continuously exposed to different concentrations of compounds. After 48 h, 20 $\mu$L of 3-(4,5-dimethylthiazol-2-yl)-2,5-diphenyltetrazolium bromide (MTT) solution were added to each well, which were incubated for another 4 h. Then 20% SDS (100 $\mu$L) were added to each well. After 12 h at room temperature, the OD value of each well was recorded at 595 nm. The $IC_{50}$ value of each compound was calculated by the Reed and Muench method.[23]

## Electronic Supplementary Material

Supplementary material is available in the online version of this article at http://dx.doi.org/10.1007/s13659-011-0007-7 and is accessible for authorized users.

## Acknowledgments

This work was financially supported by the National Basic Research Program of China (973 Program No. 2009CB522303 and No. 2011CB915503), the NSFC (No. U0932602), the National Natural Science Foundation of China (No.90813004), and the State Key Laboratory of Phytochemistry and Plant Resources in West China (No.P2010-ZZ05).

**Open Access** This article is distributed under the terms of the Creative Commons Attribution License which permits any use, distribution, and reproduction in any medium, provided the original author(s) and source are credited.

## Electronic supplementary material

Supplementary material, approximately 1.23 MB.
