# Supplementary material for: Chemical constituents from the aerial parts of Musella lasiocarpa
Source: Nat Prod Bioprospect. 2011 Sep 5;1(1):41–7. doi: 10.1007/s13659-011-0007-7 (PMC4131711; doi:10.1007/s13659-011-0007-7)

## Electronic Supplementary Material

# Chemical constituents from the aerial parts of *Musella lasiocarpa*

Liao-Bin DONG,[a,b] Juan HE,[a] Xing-Yao LI,[a,b] Xing-De WU,[a,b] Xu DENG,[a,b] Gang XU,[a] Li-Yan PENG,[a] Yu ZHAO,[a] Yan LI,[a] Xun GONG,[a] and Qin-Shi ZHAO[a,]*

[a]State Key Laboratory of Phytochemistry and Plant Resources in West China, Kunming Institute of Botany, Chinese Academy of Sciences, Kunming 650201, China

[b]Graduate School of Chinese Academy of Sciences, Beijing 100039, China

Received 3 July 2011; Accepted 26 August 2011

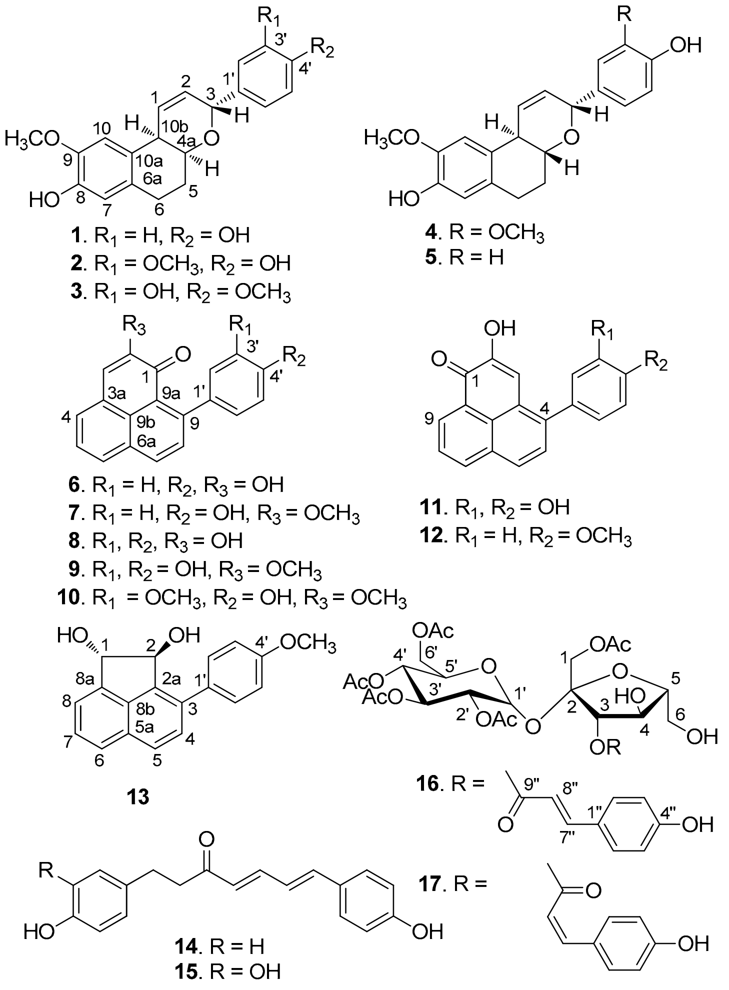


Structures of compounds **1**–**17**.

*To whom correspondence should be addressed. E-mail: qinshizhao@mail.kib.ac.cn.

**List of Electronic Supplementary Material**

- 1D, 2D NMR and HREIMS spectra of compound **2**;
- 1D NMR and HREIMS spectra of compound **3**;
- 1D, ROESY NMR and HREIMS spectra of compound **4**;
- 1D NMR and HREIMS spectra of compound **5**;
- 1D, 2D NMR, EI and HREIMS spectra of compound **9**;
- 1D NMR and HREIMS spectra of compound **10**;
- 1D, 2DNMR and HRESIMS spectra of compound **13**;
- 1D, 2DNMR and HRESIMS spectra of compound **16**;
- 1D NMR and HRESIMS spectra of compound **17**;
- ESIMS spectrum of sucrose.

Figure S1. $^{1}$H NMR Spectrum of Compound **2**.

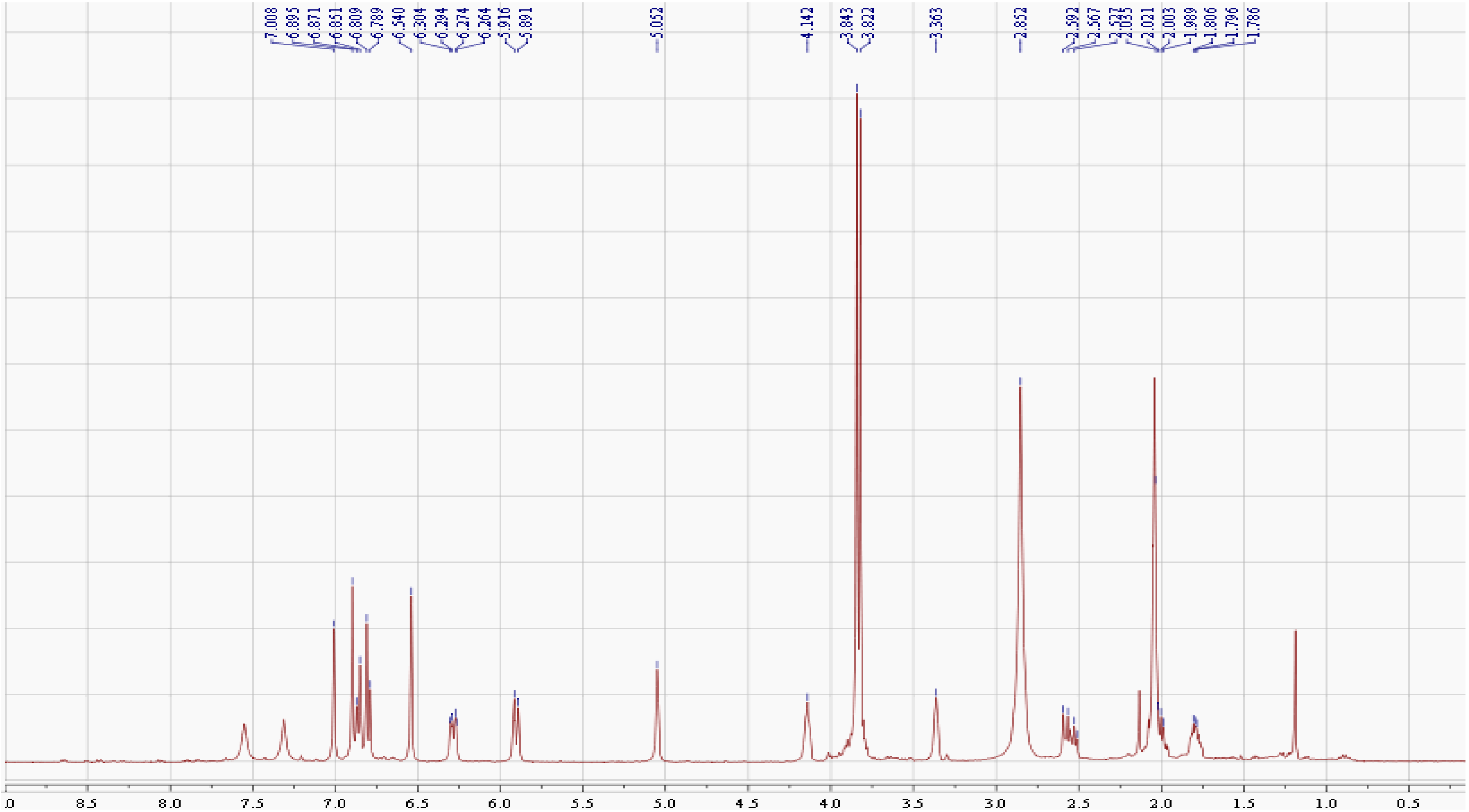

Figure S2. $^{13}C$ NMR Spectrum of Compound **2**.

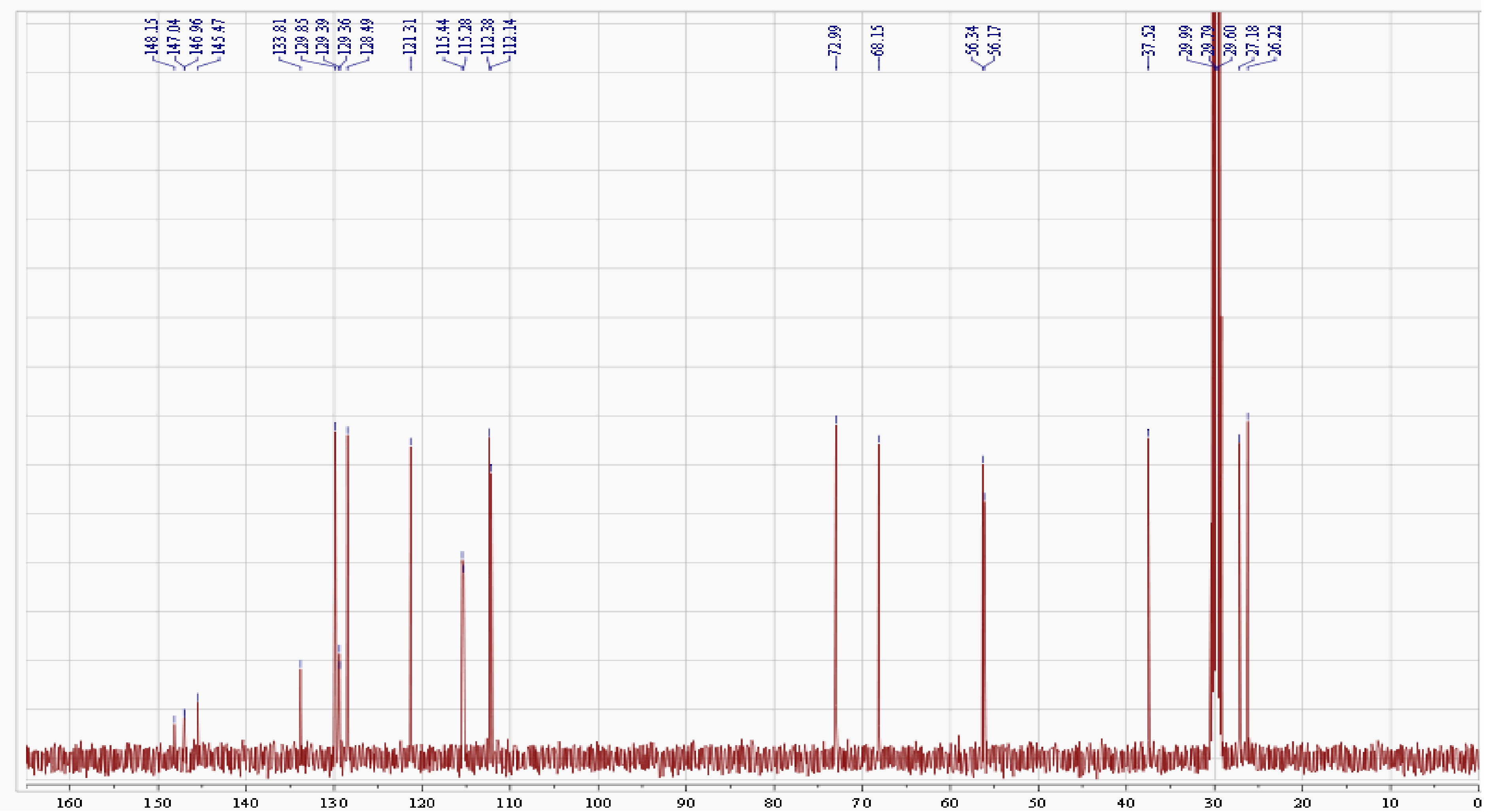

Figure S3. $^{13}$C DEPT 135 Spectrum of Compound **2.**

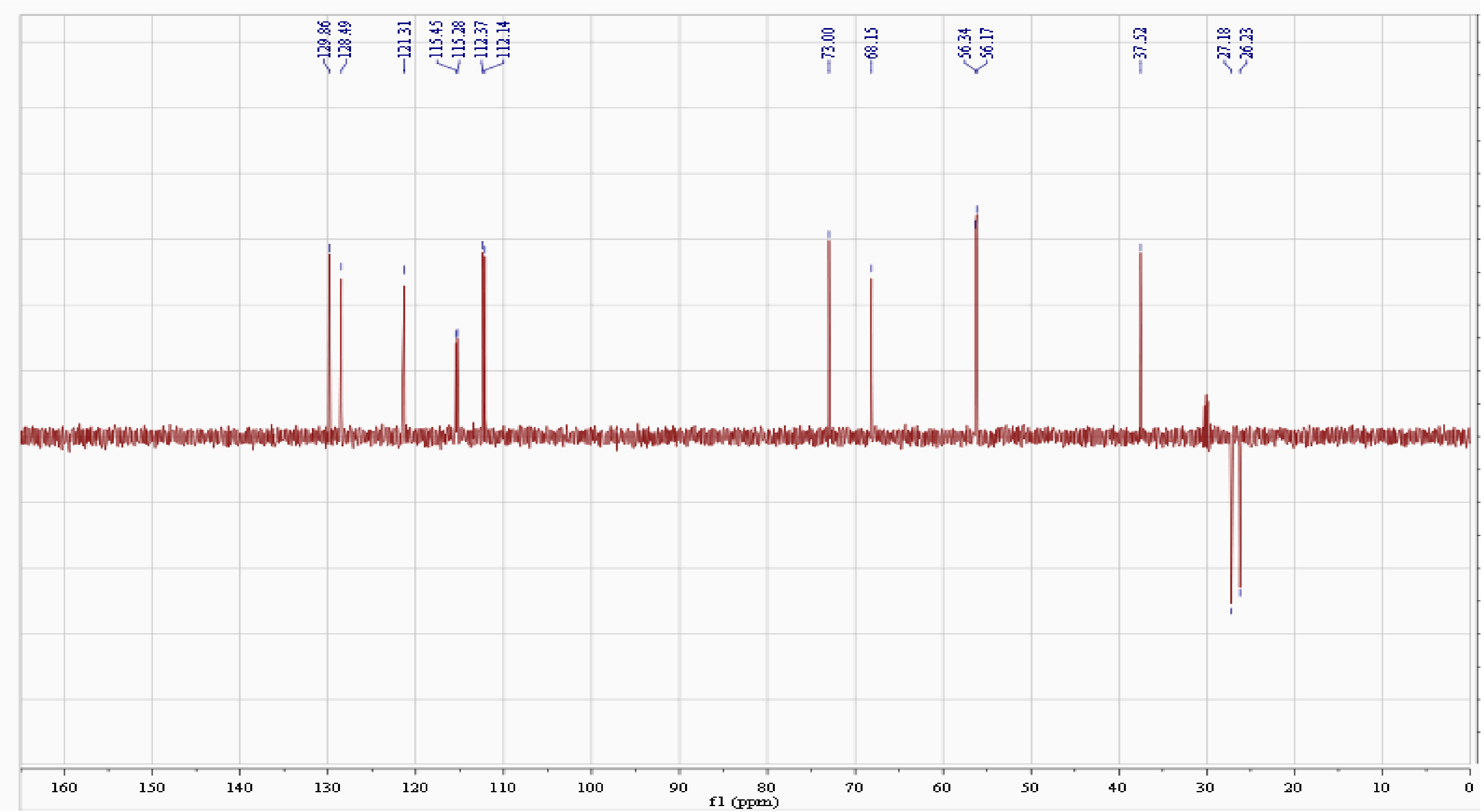

Figure S4. HSQC Spectrum of Compound **2**.

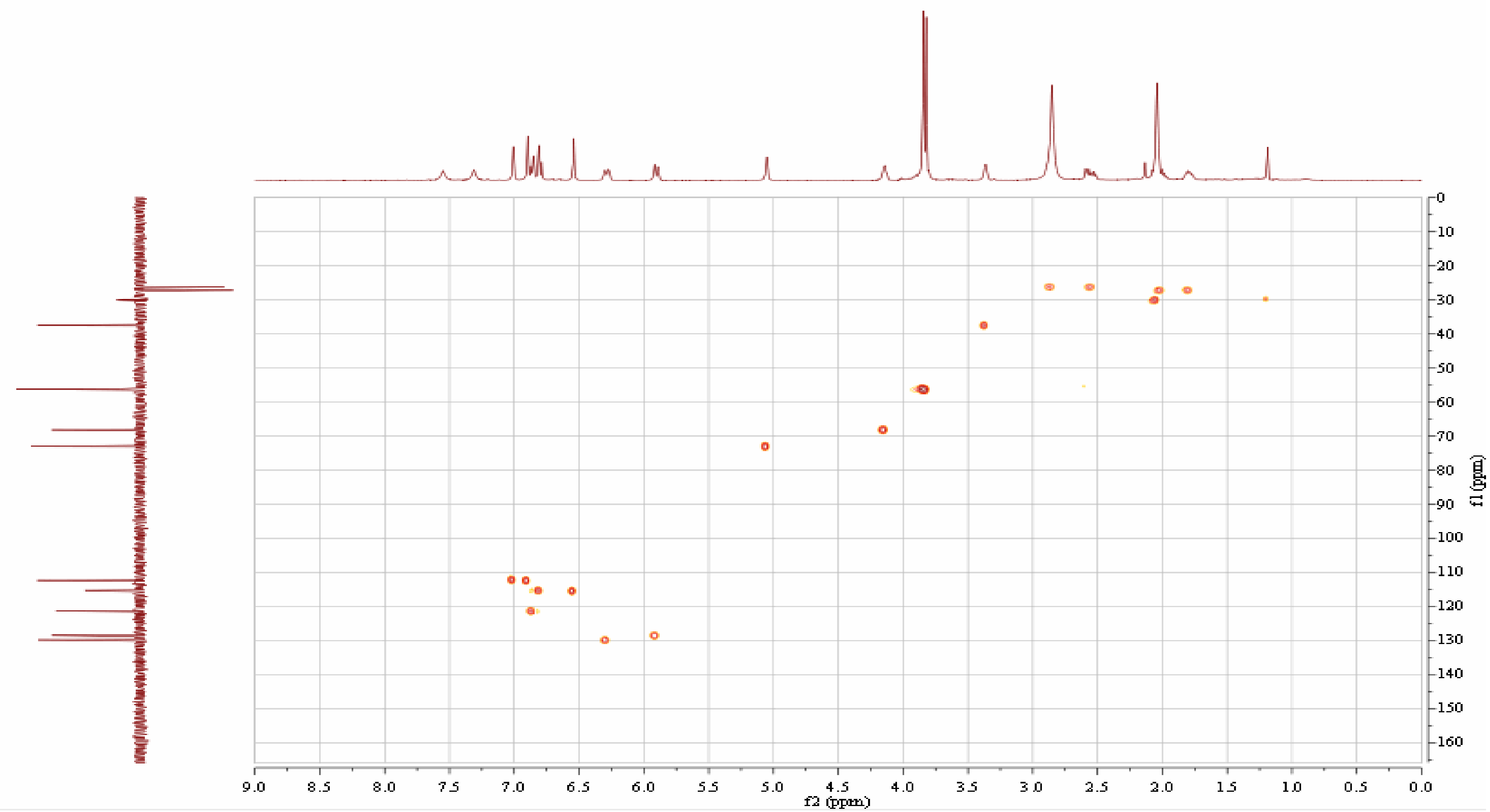

Figure S5. COSY Spectrum of Compound **2**.

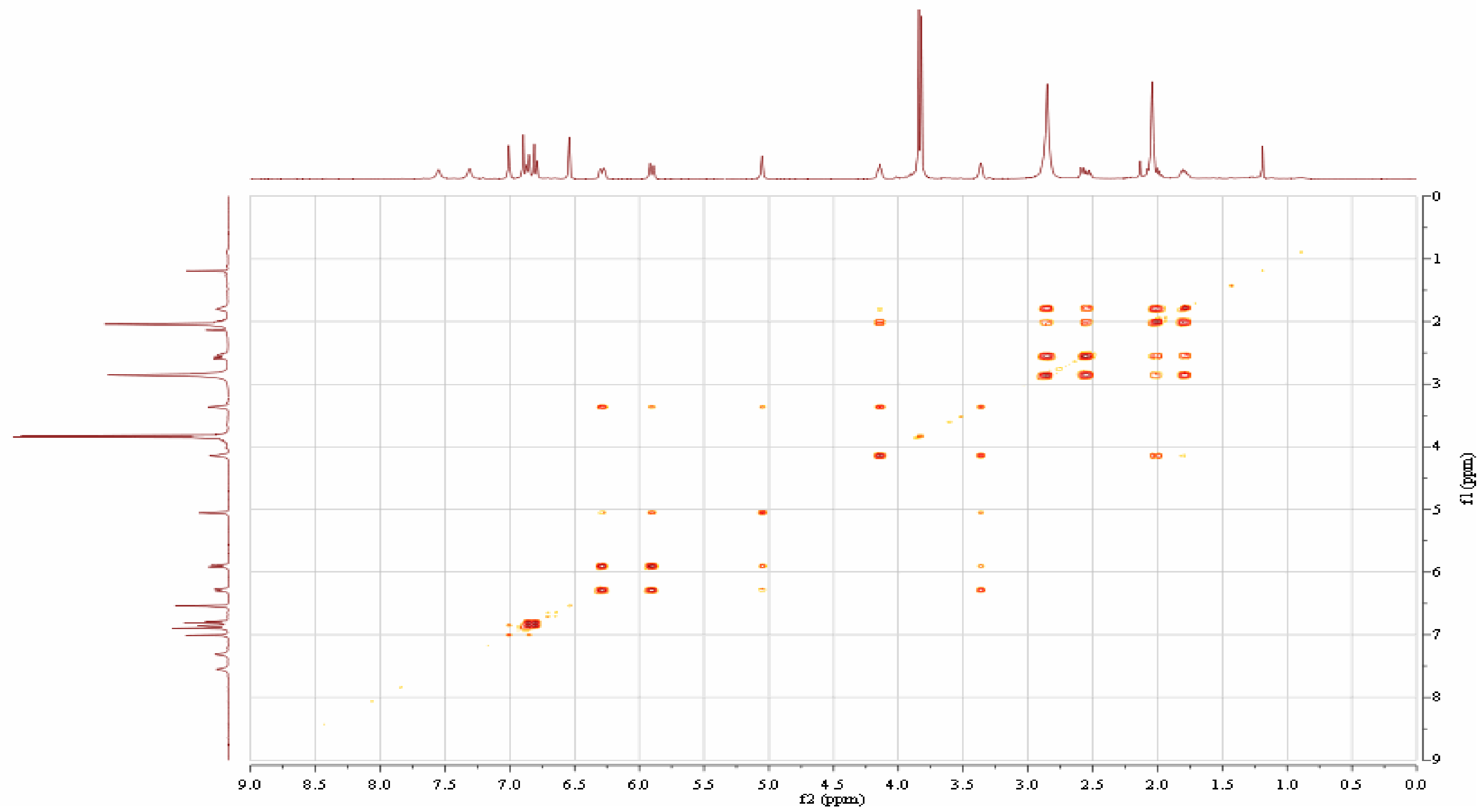

Figure S6. HMBC Spectrum of Compound **2**.

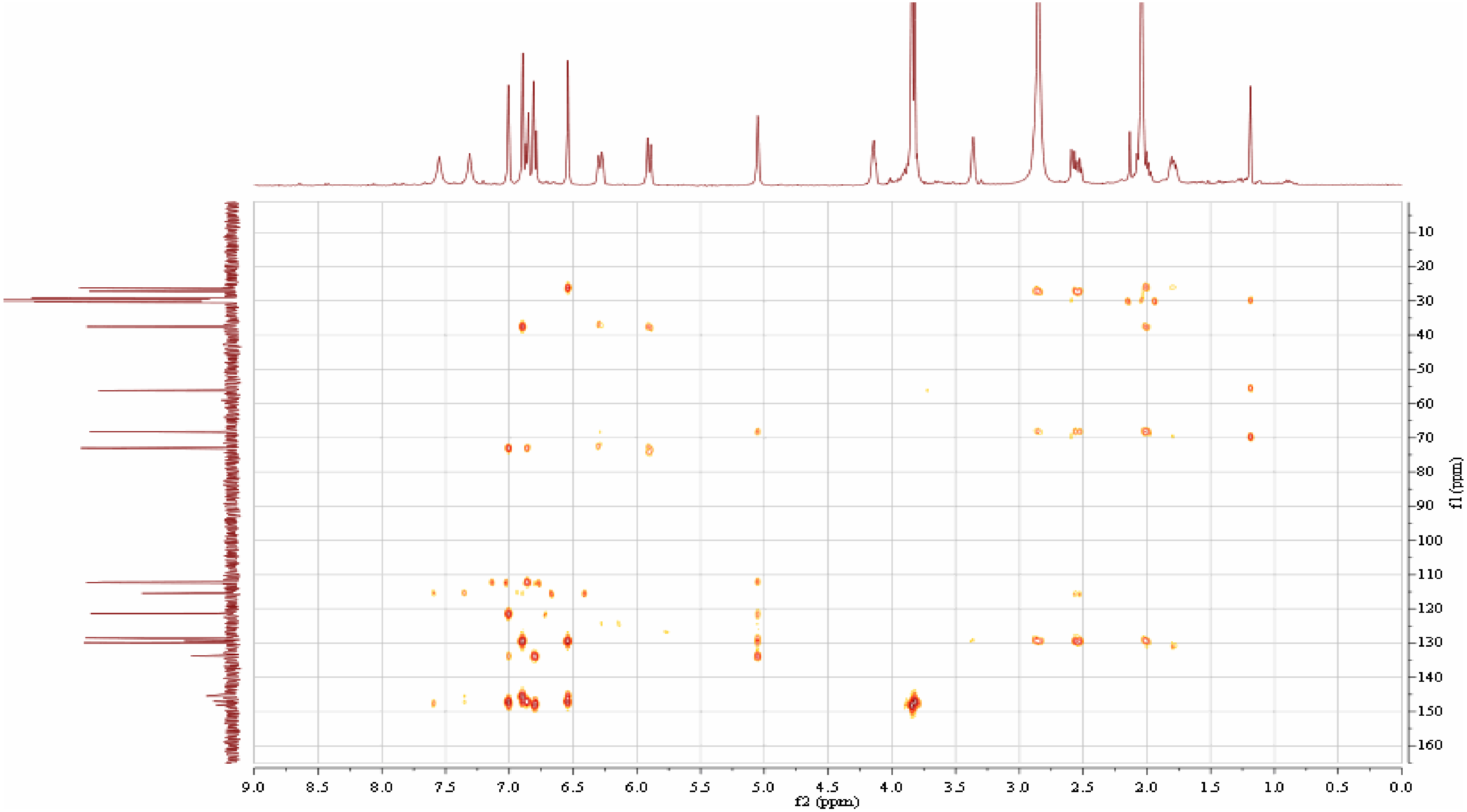

Figure S7. ROESY Spectrum of Compound **2**.

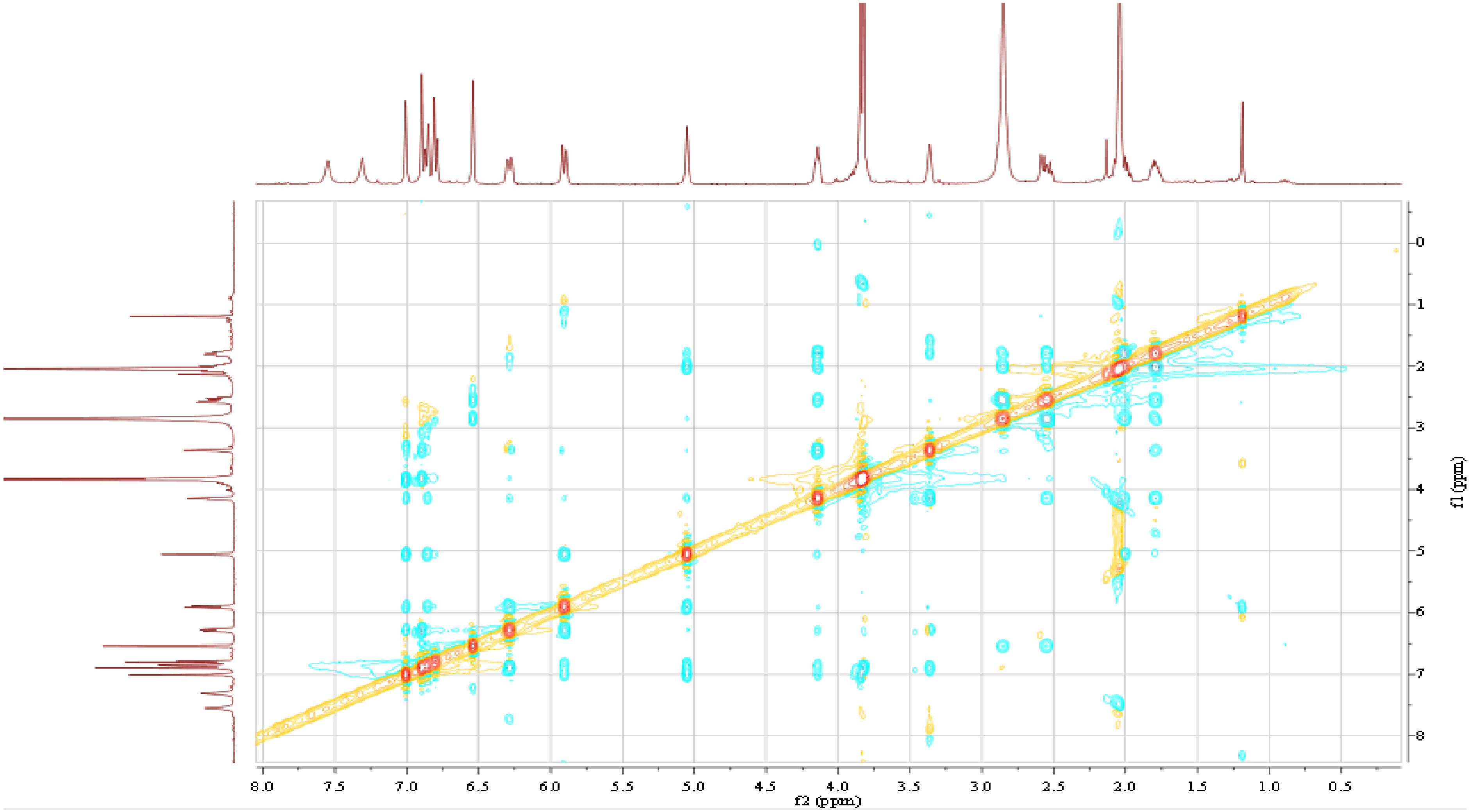

Figure S8. HREIMS Spectrum of Compound **2**.



## Elemental Composition Report

**Single Mass Analysis (displaying only valid results)**
Tolerance = 10.0 mDa / DBE: min = 0.5, max = 40.0
Selected filters: None

Monoisotopic Mass, Odd and Even Electron Ions
34 formula(e) evaluated with 2 results within limits (up to 51 closest results for each mass)
Elements Used:
C: 0-200 H: 0-400 O: 0-7

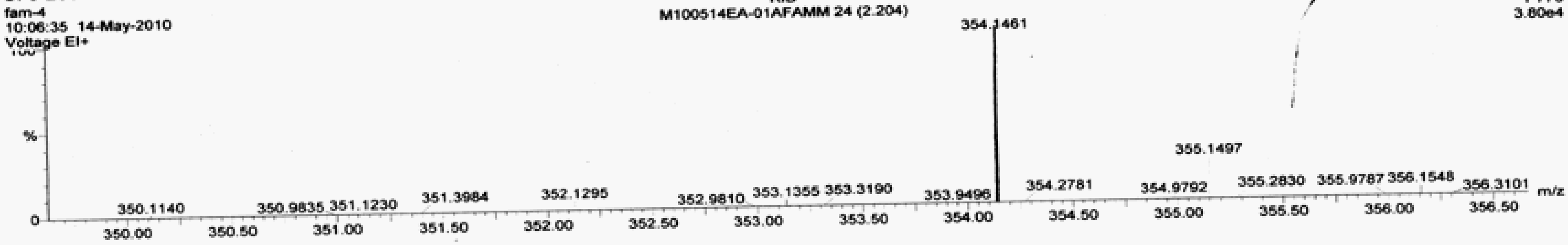


| | | | | | | |
|---|---|---|---|---|---|---|
| Minimum: | | | | 0.5 | | |
| Maximum: | | 10.0 | 10.0 | 40.0 | | |
| Mass | Calc. Mass | mDa | PPM | DBE | i-FIT | Formula |
| 354.1461 | 354.1467 | -0.6 | -1.7 | 11.0 | 65.7 | C21 H22 O5 |
| | 354.1409 | 5.2 | 14.7 | 20.0 | 322.7 | C28 H18 |

Figure S9. $^{1}$H NMR Spectrum of Compound **3**.

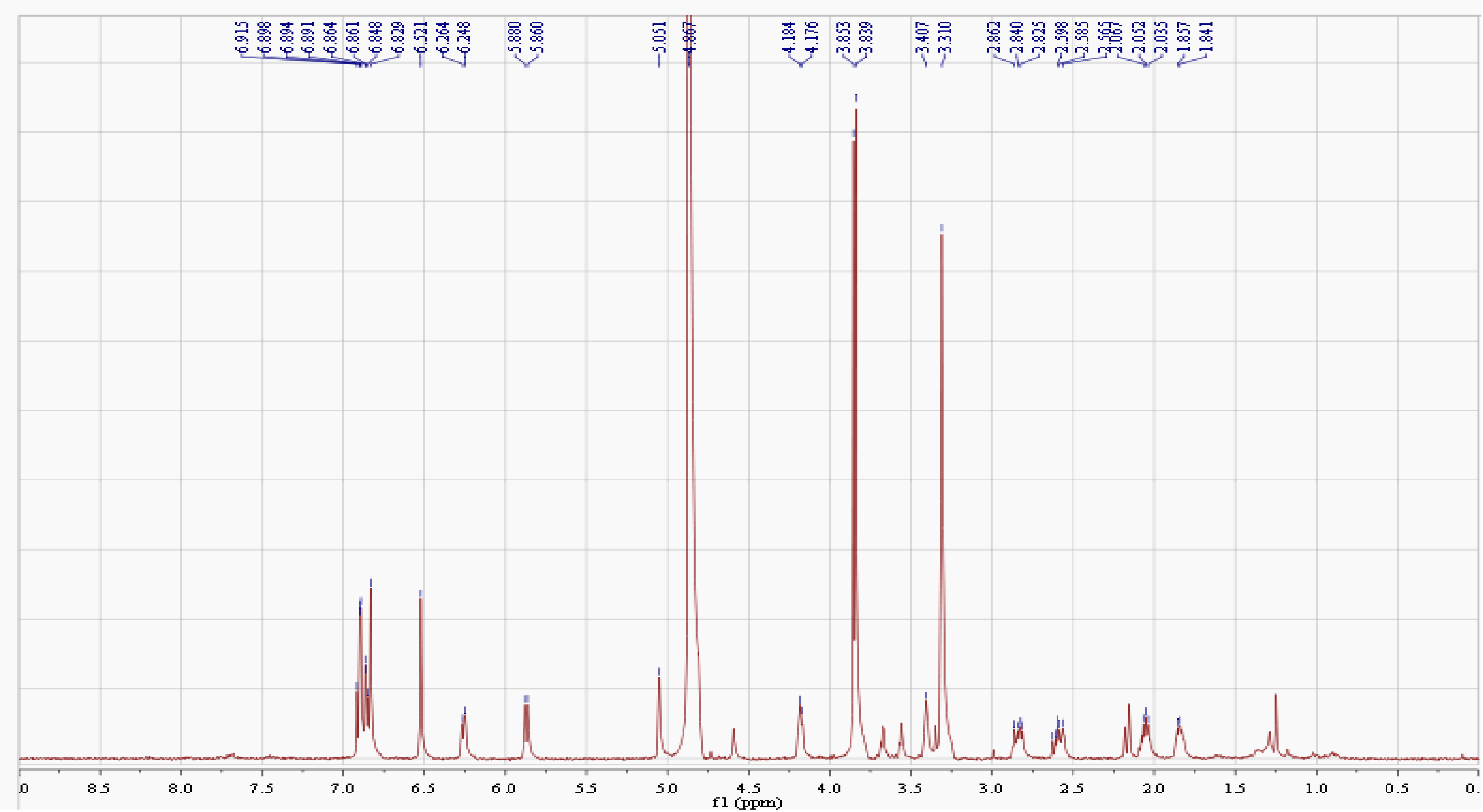

Figure S10. $^{13}$C NMR Spectrum of Compound **3**.

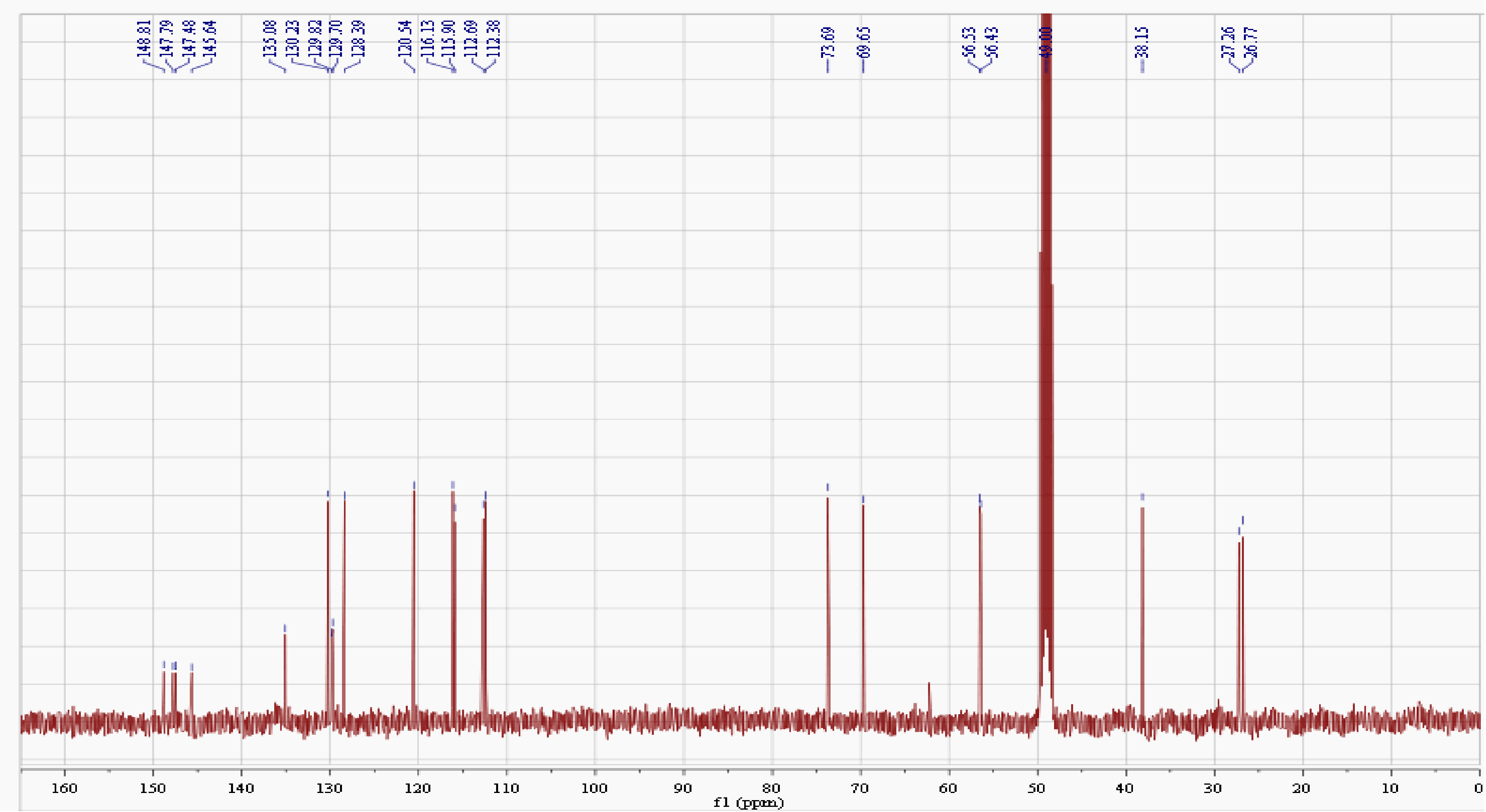

Figure S11. HREIMS Spectrum of Compound **3**.

**[illegible]al Composition Report** **Page 1**

**Single Mass Analysis (displaying only valid results)**
Tolerance = 10.0 mDa / DBE: min = 0.5, max = 40.0
Selected filters: None

Monoisotopic Mass, Odd and Even Electron Ions
26 formula(e) evaluated with 2 results within limits (up to 51 closest results for each mass)
Elements Used:
C: 0-200 H: 0-400 O: 0-5

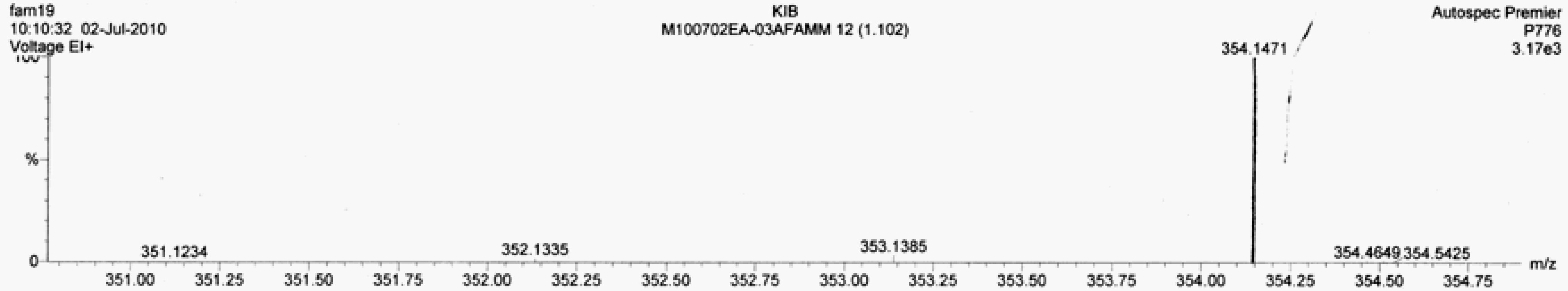


| | | | | | | |
|---|---|---|---|---|---|---|
| Minimum: | | | | 0.5 | | |
| Maximum: | | 10.0 | 10.0 | 40.0 | | |
| Mass | Calc. Mass | mDa | PPM | DBE | i-FIT | Formula |
| 354.1471 | 354.1467 | 0.4 | 1.1 | 11.0 | 5547598.5 | C21 H22 O5 |
| | 354.1409 | 6.2 | 17.5 | 20.0 | 5547606.5 | C28 H18 |

Figure S12. $^{1}$H NMR Spectrum of Compound **4**.

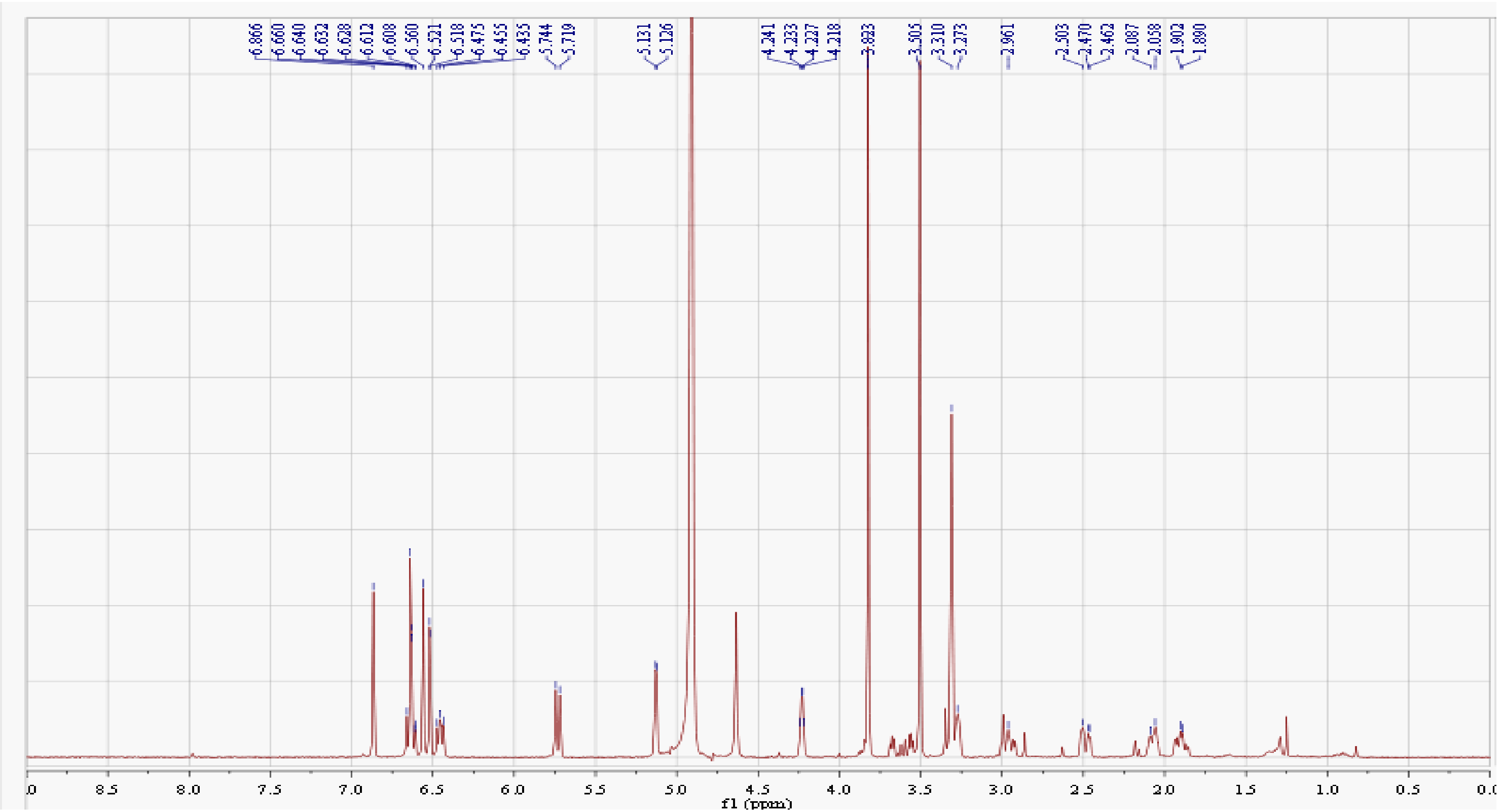

Figure S13. $^{13}C$ NMR Spectrum of Compound **4**.

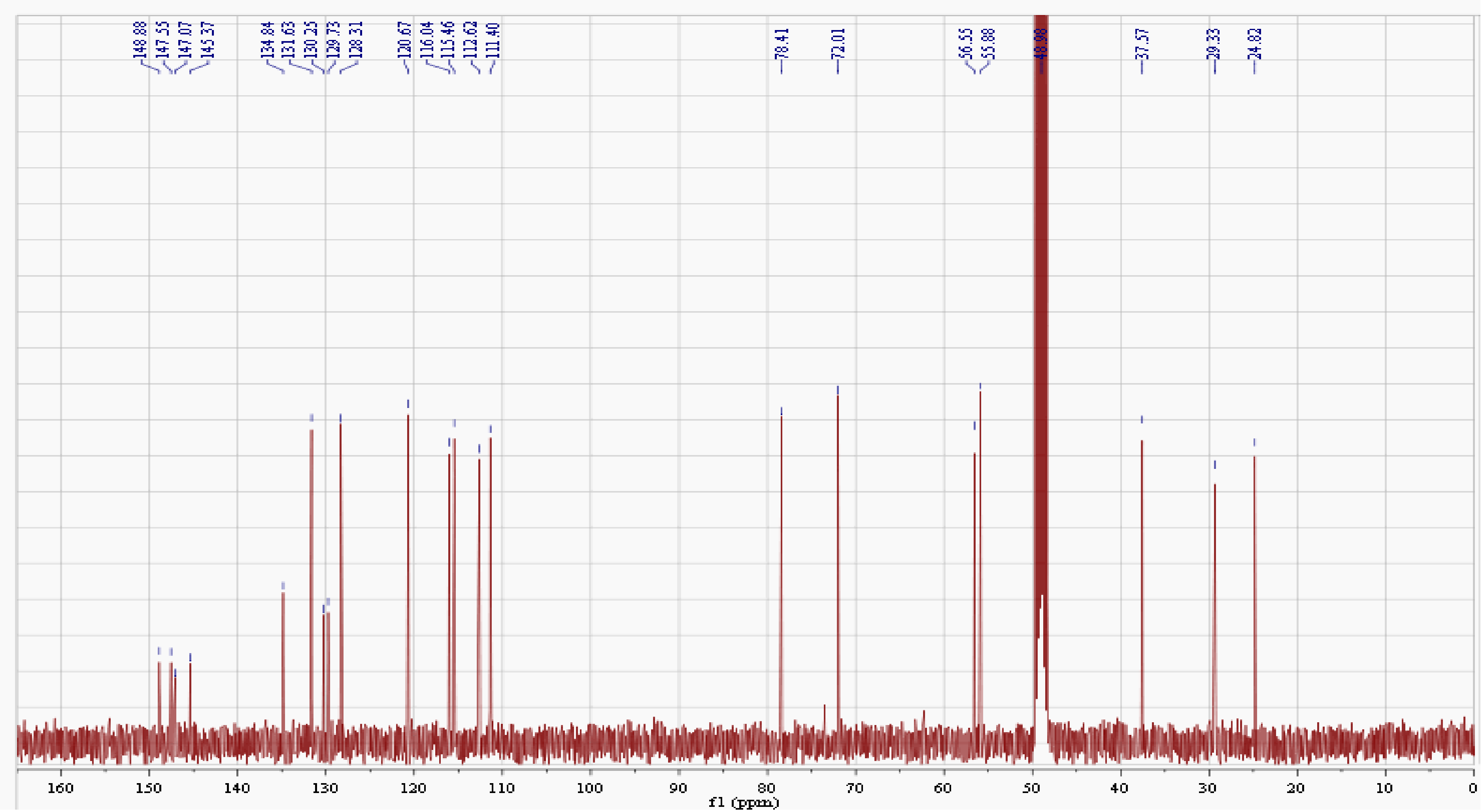

Figure S14. ROESY Spectrum of Compound **4**.

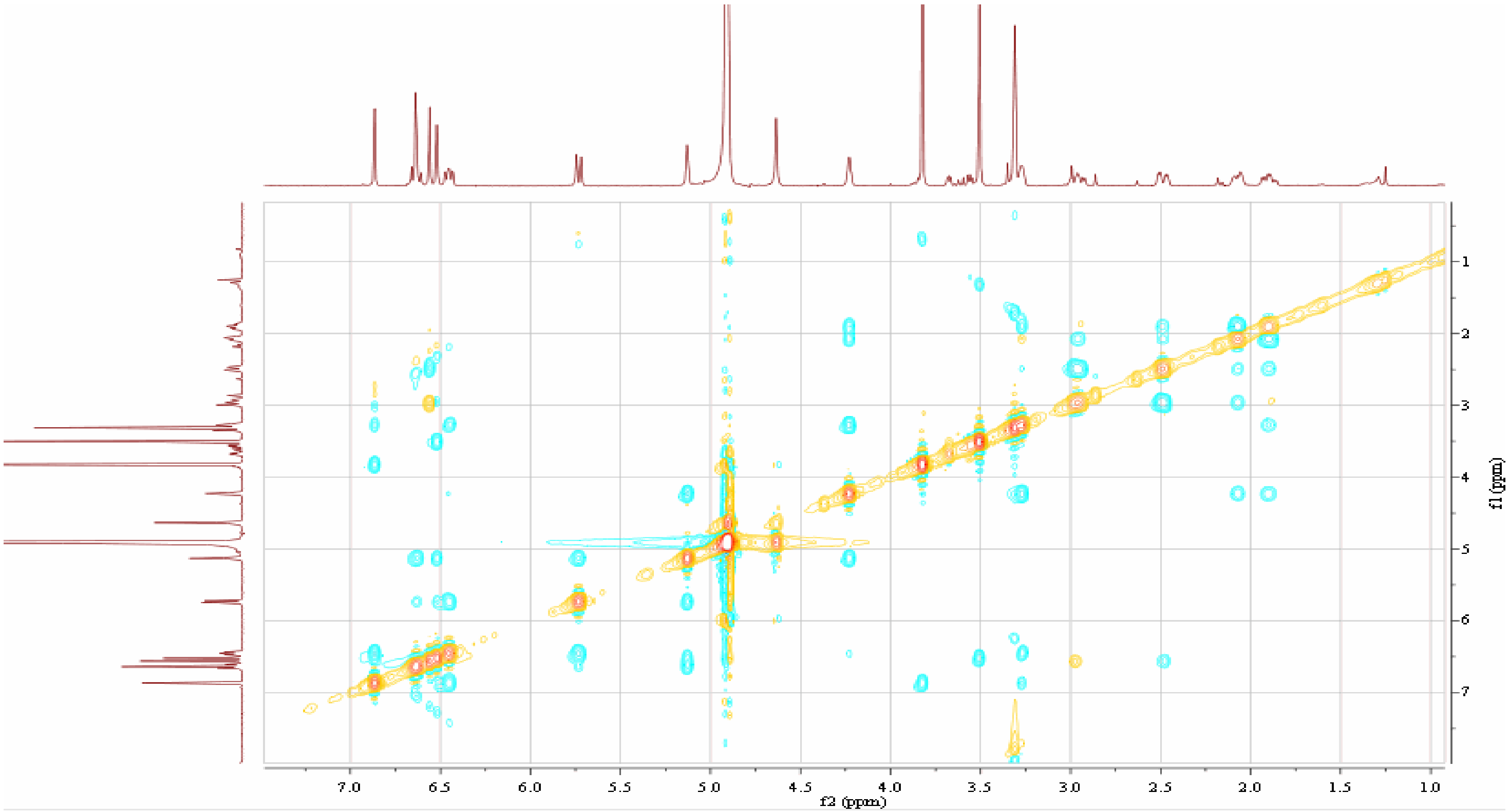

Figure S15. HREIMS Spectrum of Compound **4**.

## [illegible]mental Composition Report

Page 1

**Single Mass Analysis (displaying only valid results)**
Tolerance = 10.0 mDa / DBE: min = 0.5, max = 40.0
Selected filters: None

Monoisotopic Mass, Odd and Even Electron Ions
26 formula(e) evaluated with 2 results within limits (up to 51 closest results for each mass)
Elements Used:
C: 0-200 H: 0-400 O: 0-5

fam18
10:07:42 02-Jul-2010
Voltage EI+

KIB
M100702EA-02AFAMM 7 (0.643)

Autospec Premier
P776
4.64e4

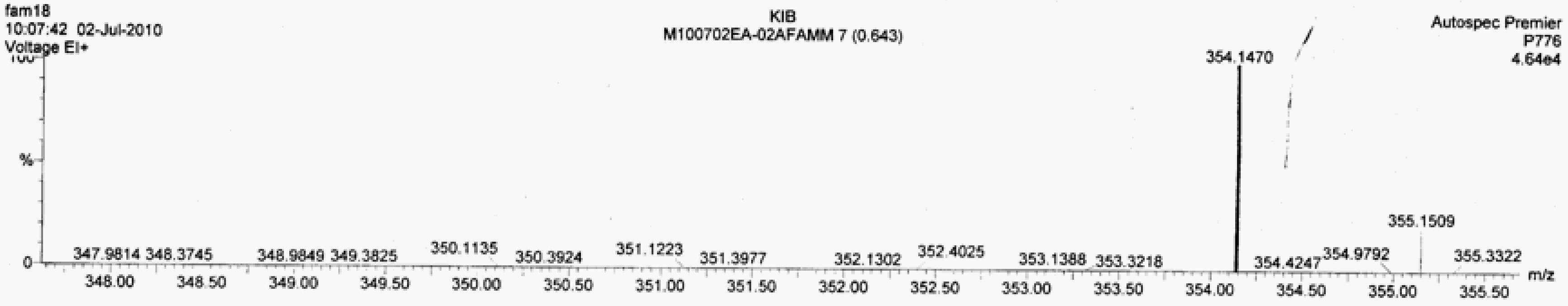


| | | | | | | |
|---|---|---|---|---|---|---|
| Minimum: | | | | 0.5 | | |
| Maximum: | | 10.0 | 10.0 | 40.0 | | |
| Mass | Calc. Mass | mDa | PPM | DBE | i-FIT | Formula |
| 354.1470 | 354.1467 | 0.3 | 0.8 | 11.0 | 2800546.8 | C21 H22 O5 |
| | 354.1409 | 6.1 | 17.2 | 20.0 | 2800768.0 | C28 H18 |

Figure S16. $^{1}H$ NMR Spectrum of Compound **5**.

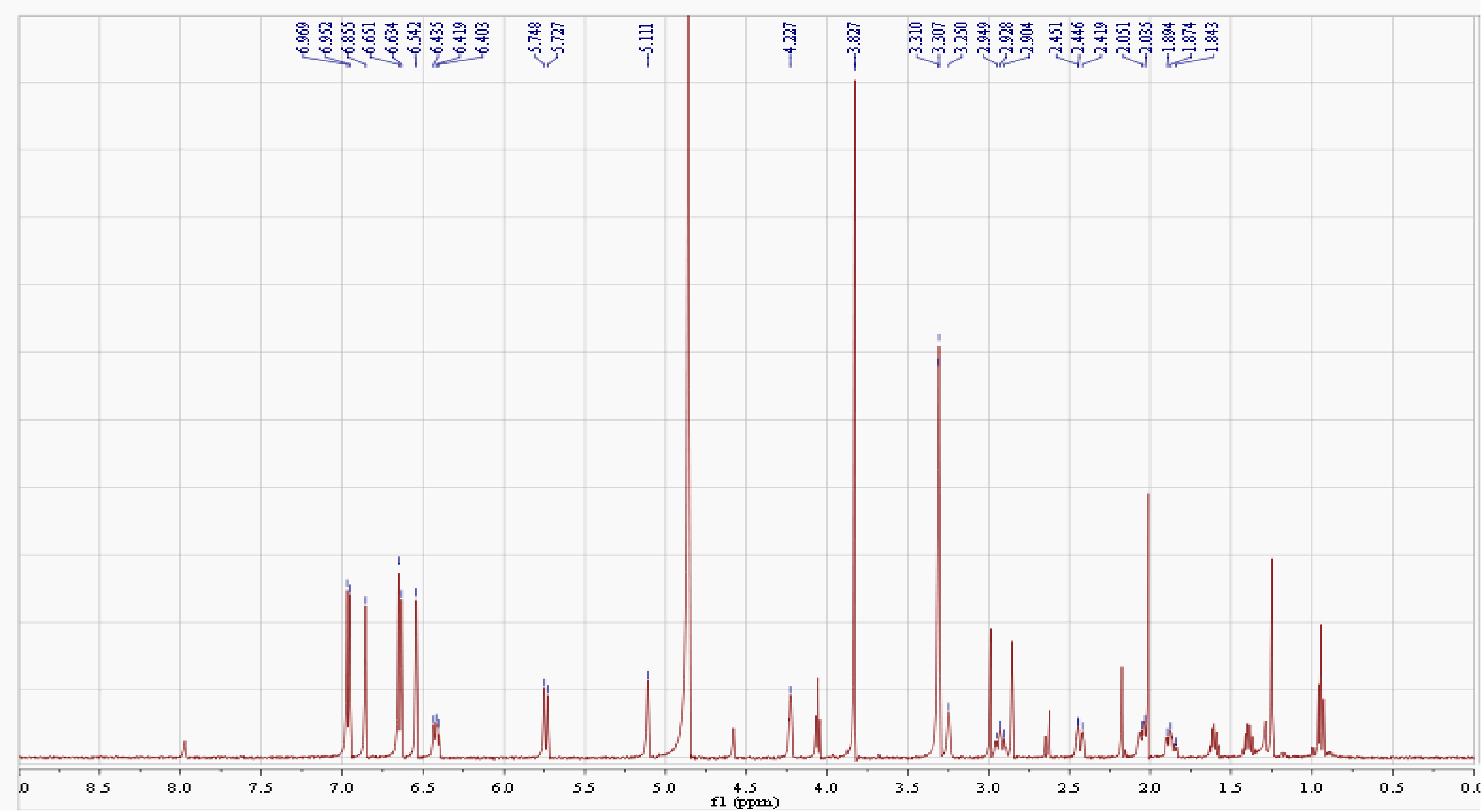

Figure S17. $^{13}$C NMR Spectrum of Compound **5**.

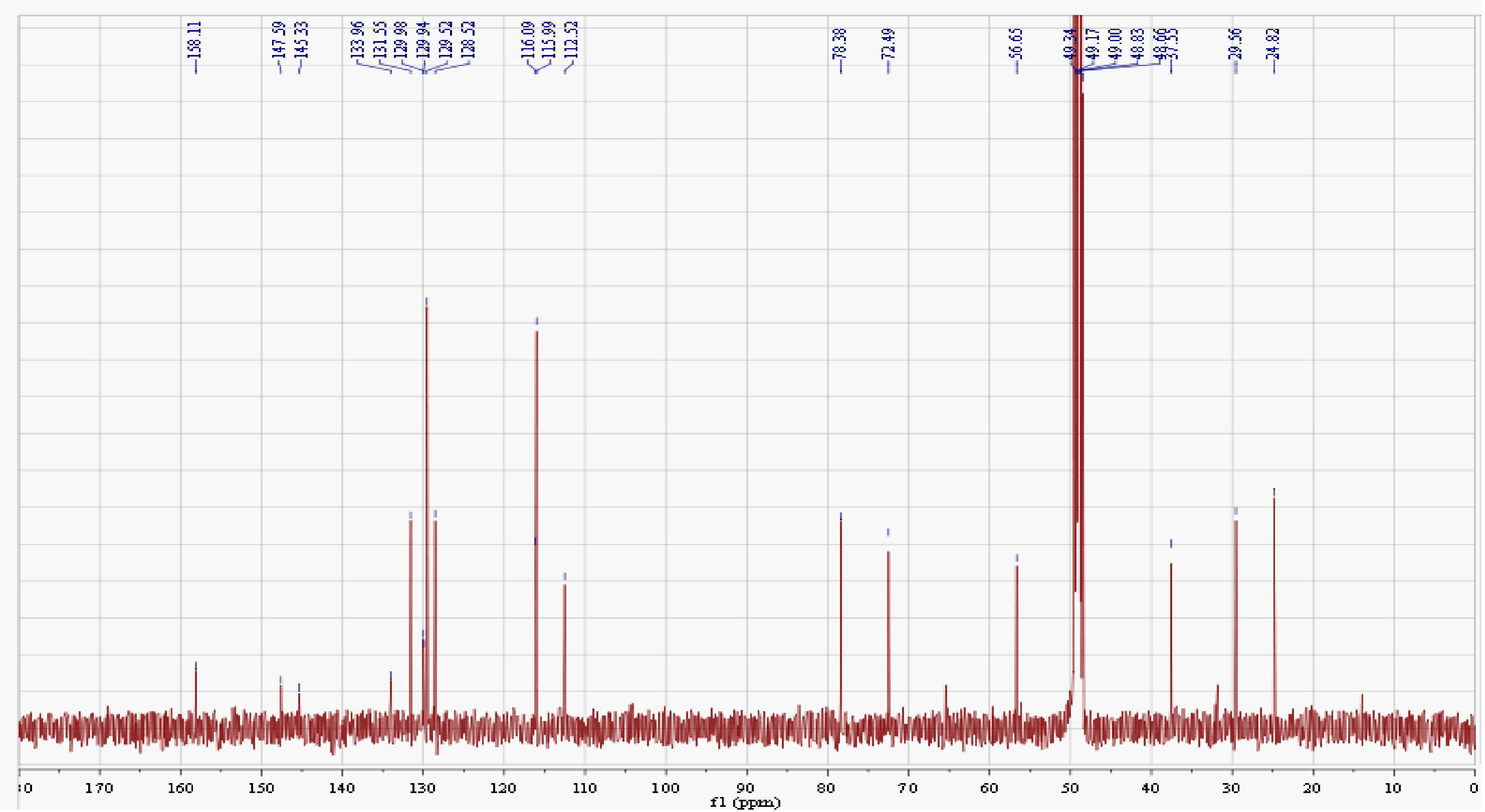

Figure S18. HREIMS Spectrum of Compound **5**.

**...mental Composition Report**

**Page 1**

**Single Mass Analysis (displaying only valid results)**
Tolerance = 10.0 mDa / DBE: min = 0.5, max = 40.0
Selected filters: None

Monoisotopic Mass, Odd and Even Electron Ions
25 formula(e) evaluated with 1 results within limits (up to 51 closest results for each mass)
Elements Used:
C: 0-200 H: 0-400 O: 0-5
fam14a
10:03:19 02-Jul-2010
Voltage EI+

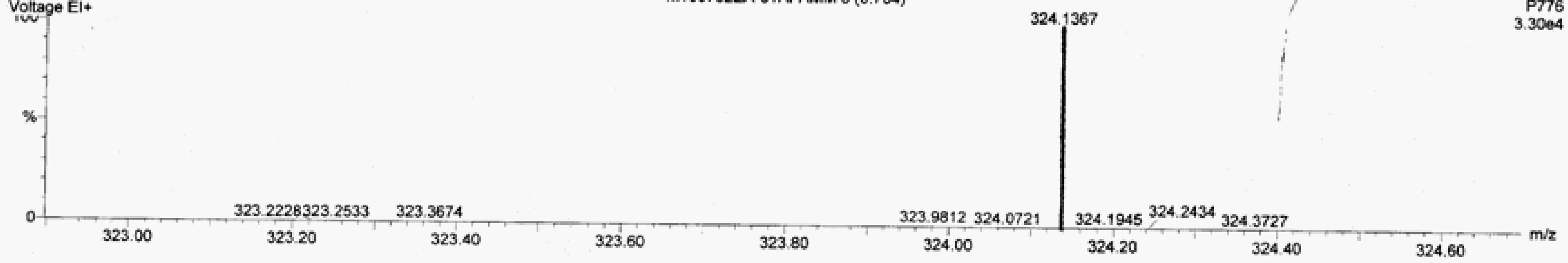


| | | | | | | |
|---|---|---|---|---|---|---|
| Minimum: | | | | 0.5 | | |
| Maximum: | | 10.0 | 10.0 | 40.0 | | |
| Mass | Calc. Mass | mDa | PPM | DBE | i-FIT | Formula |
| 324.1367 | 324.1362 | 0.5 | 1.5 | 11.0 | 5562516.0 | C20 H20 O4 |

Figure S19. $^{1}$H NMR Spectrum of Compound **9**.

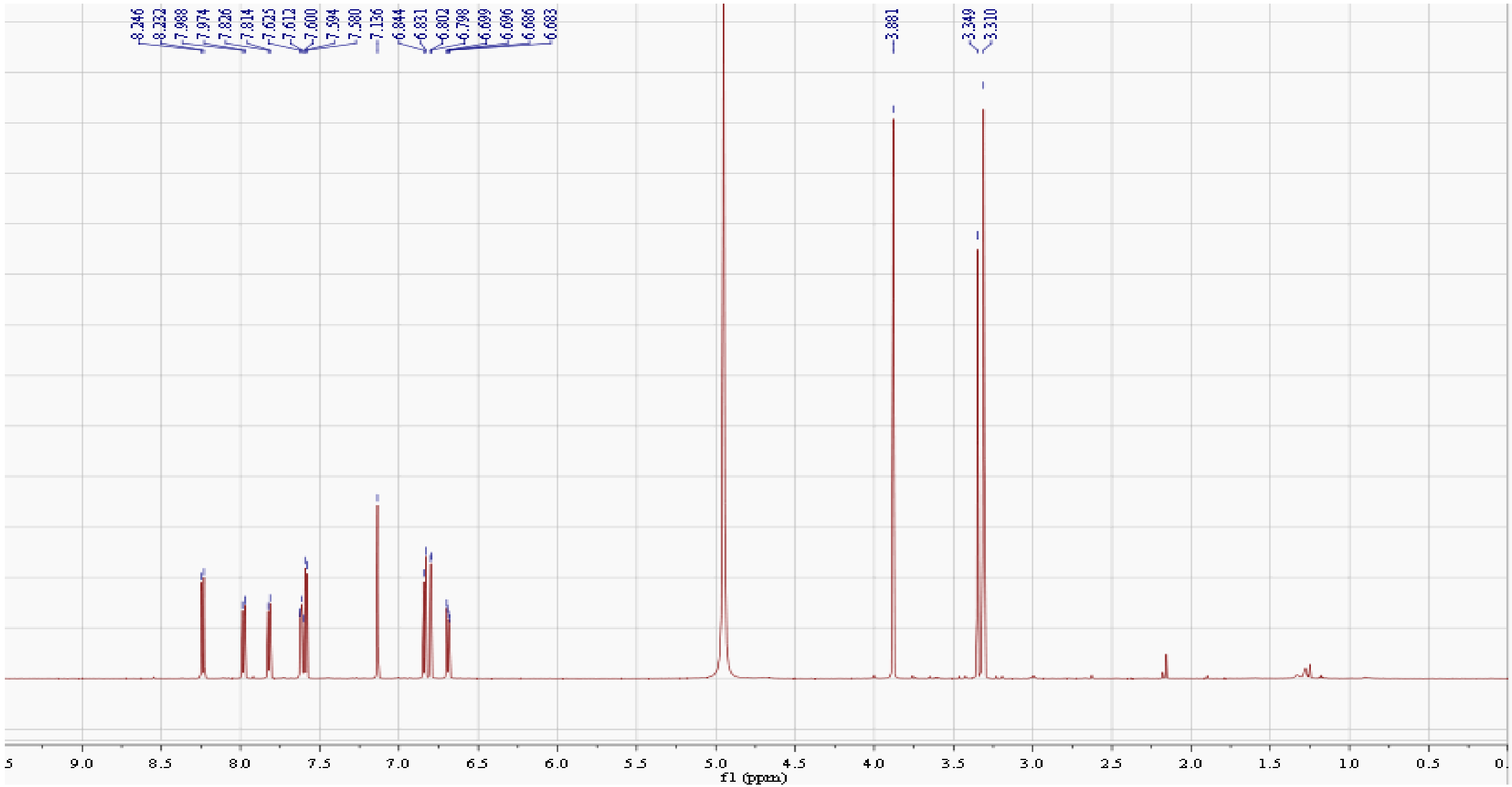

Figure S20. $^{13}$C NMR Spectrum of Compound **9**.

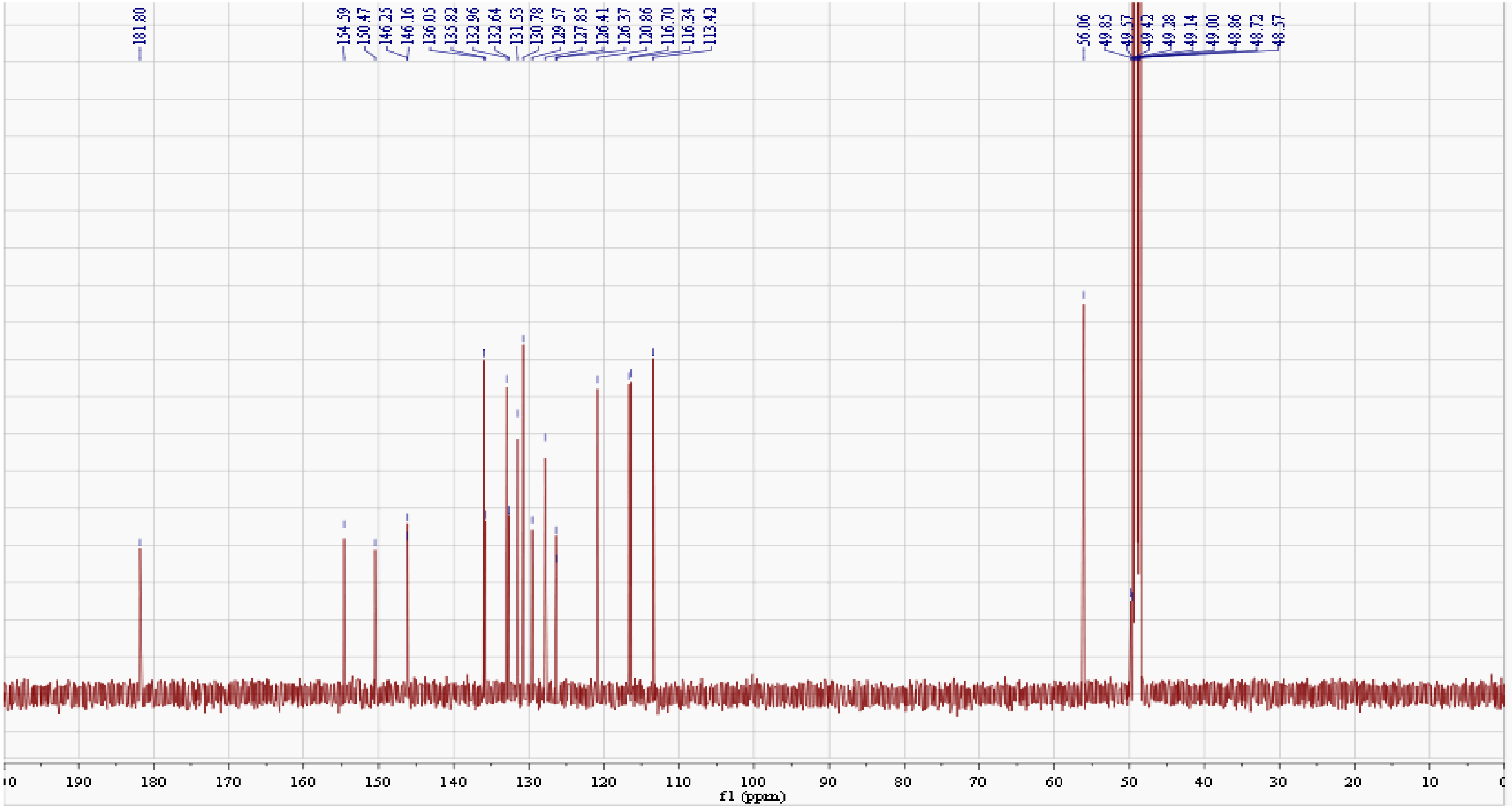

Figure S21. $^{13}$C DEPT 135 Spectrum of Compound **9**.

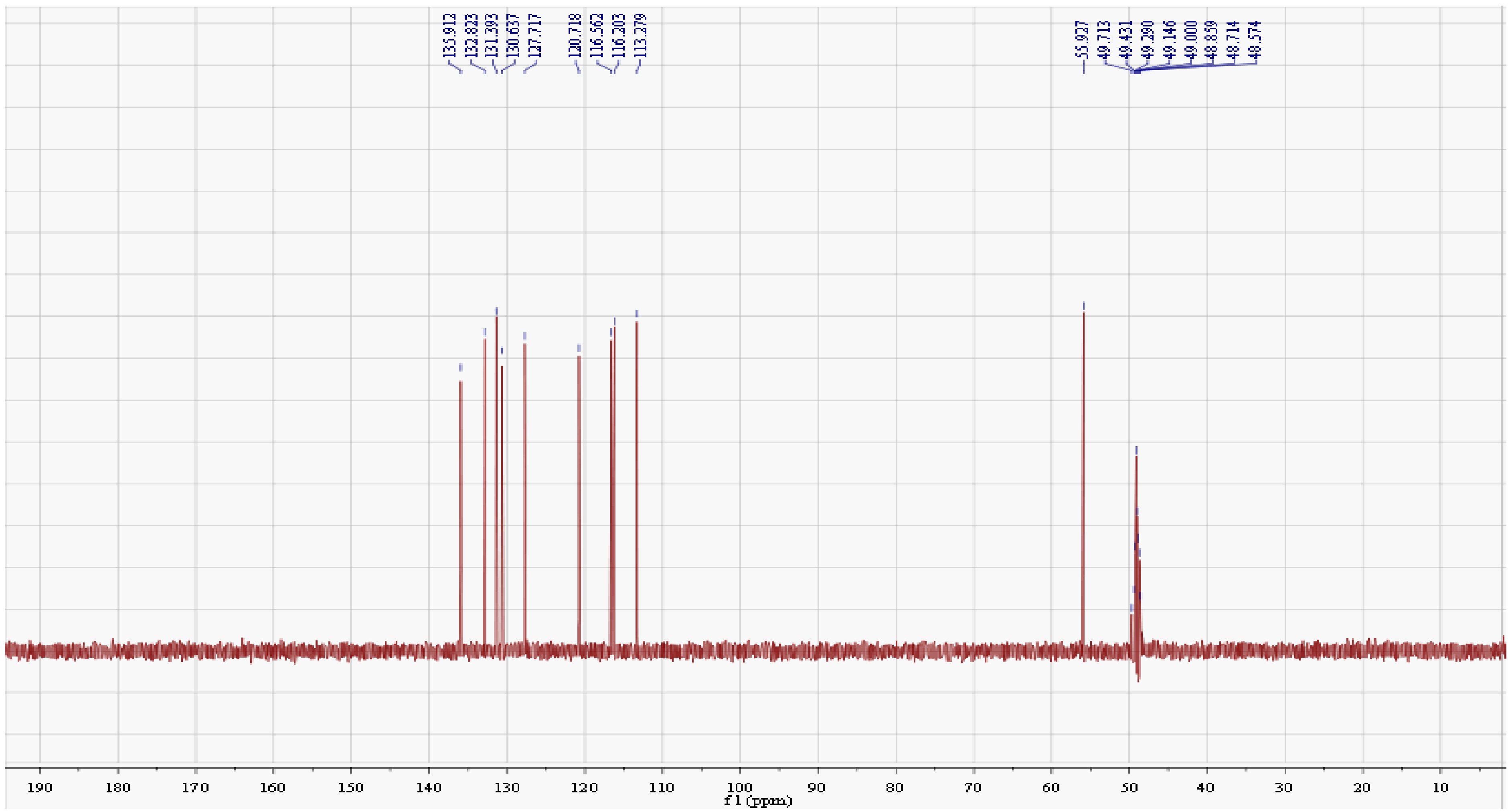

Figure S22. HSQC Spectrum of Compound **9**.

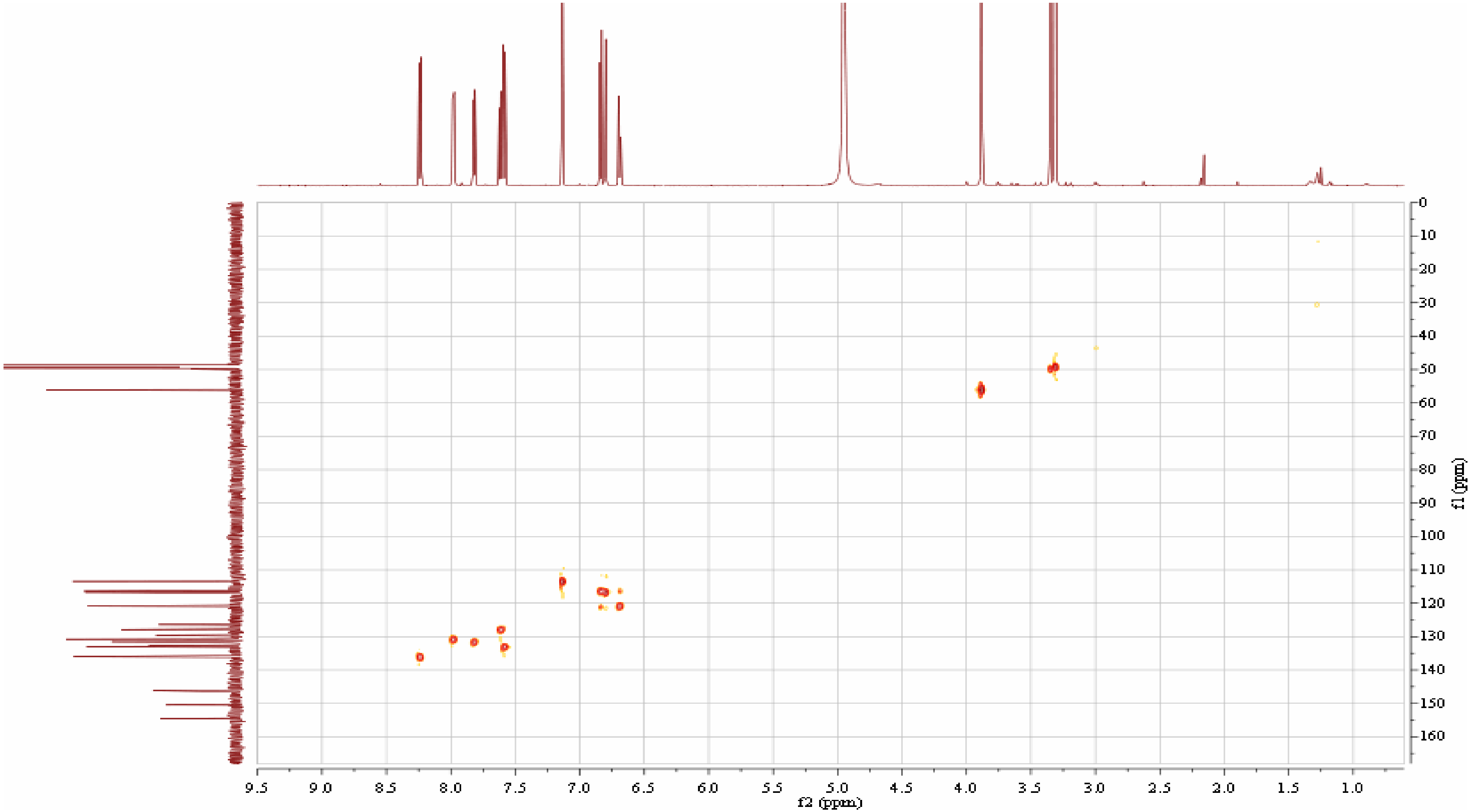

Figure S23. COSY Spectrum of Compound **9**.

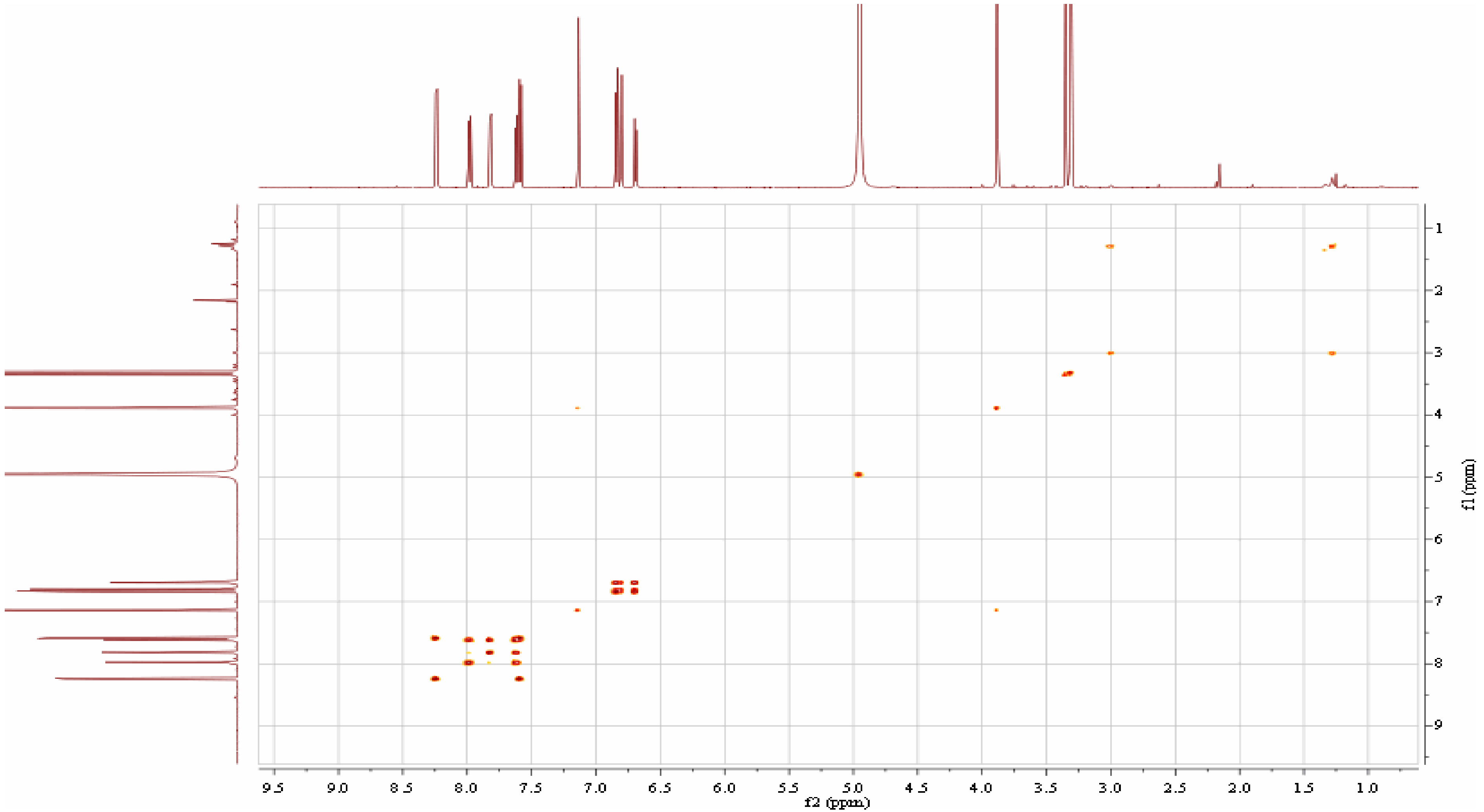

Figure S24. HMBC Spectrum of Compound **9**.

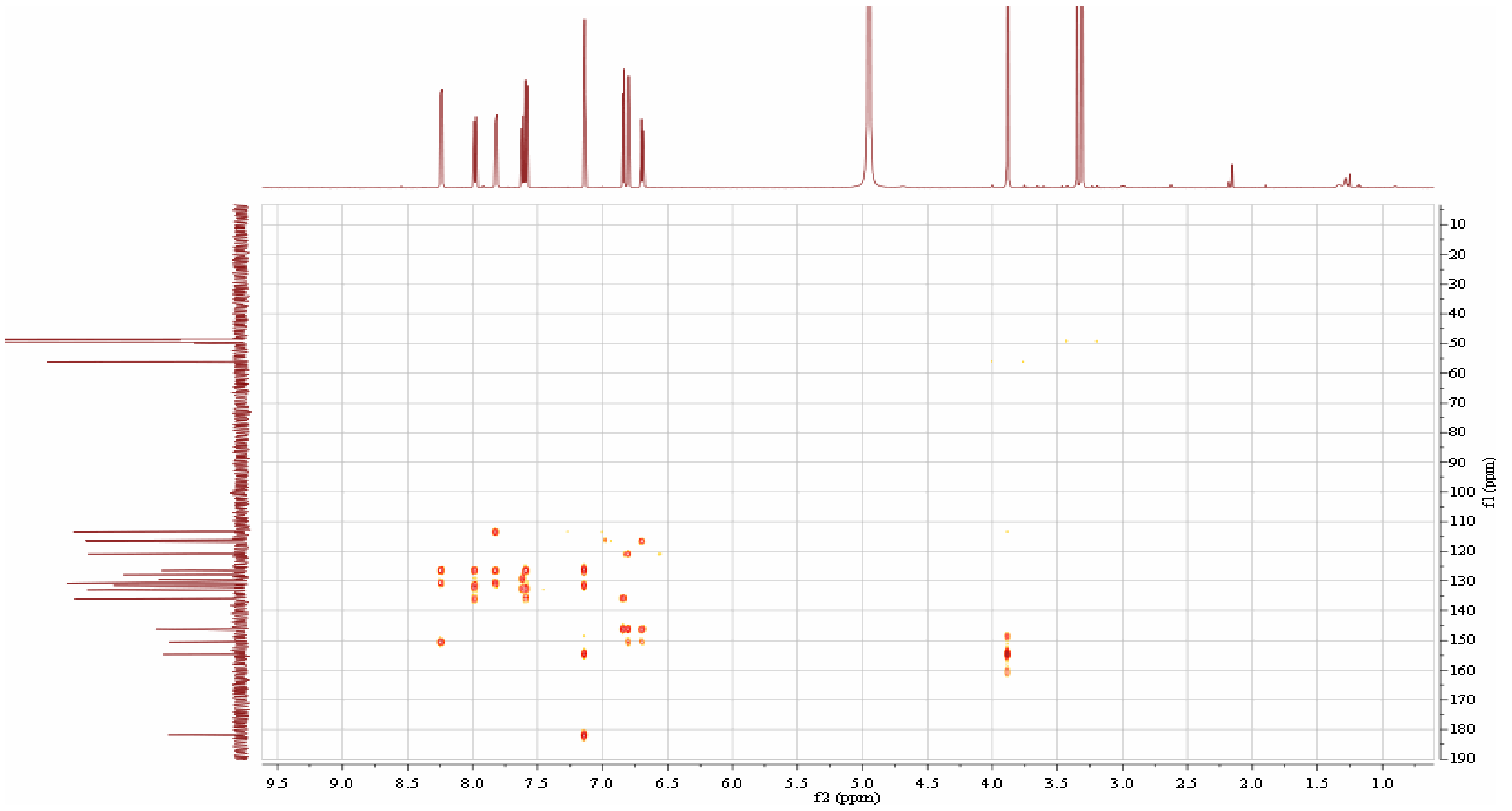

Figure S25. ROESY Spectrum of Compound **9**.

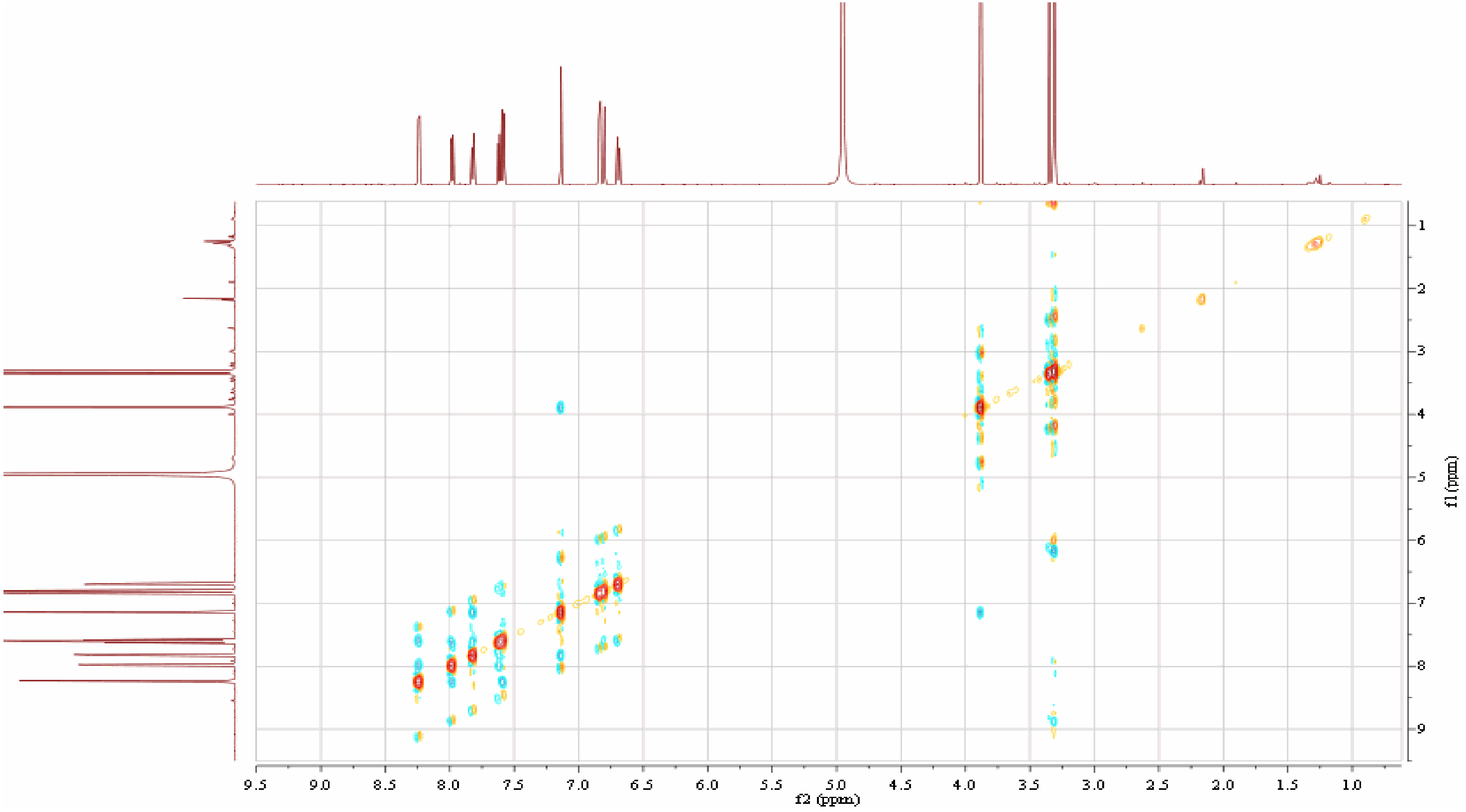

Figure S26. EIMS Spectrum of Compound **9**.

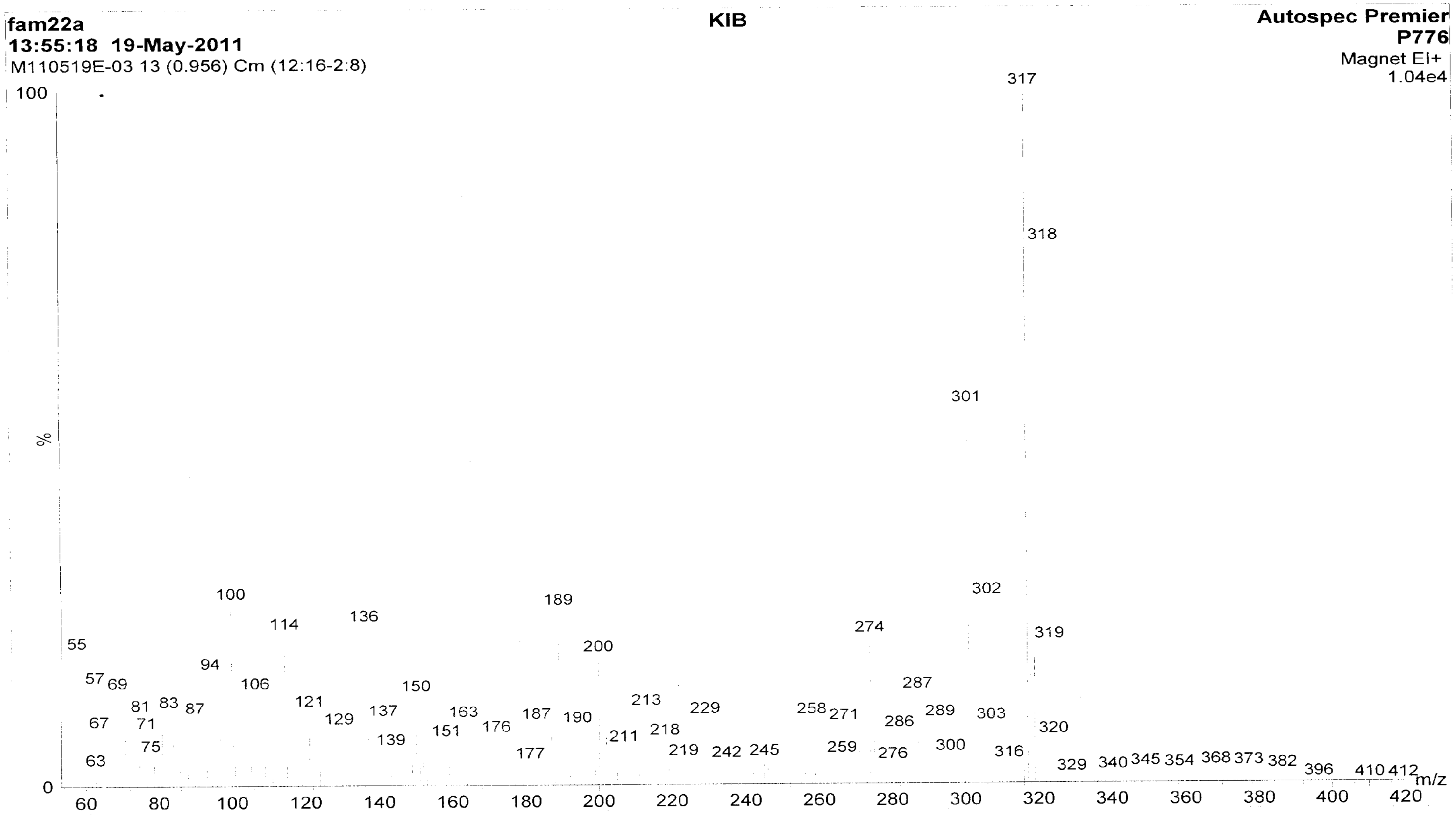

Figure S27. HREIMS Spectrum of Compound **9**.



**..ental Composition Report**

**Single Mass Analysis (displaying only valid results)**
Tolerance = 10.0 PPM / DBE: min = 0.5, max = 40.0
Selected filters: None

Monoisotopic Mass, Odd and Even Electron Ions
24 formula(e) evaluated with 1 results within limits (up to 51 closest results for each mass)
Elements Used:
C: 0-200 H: 0-400 O: 0-5

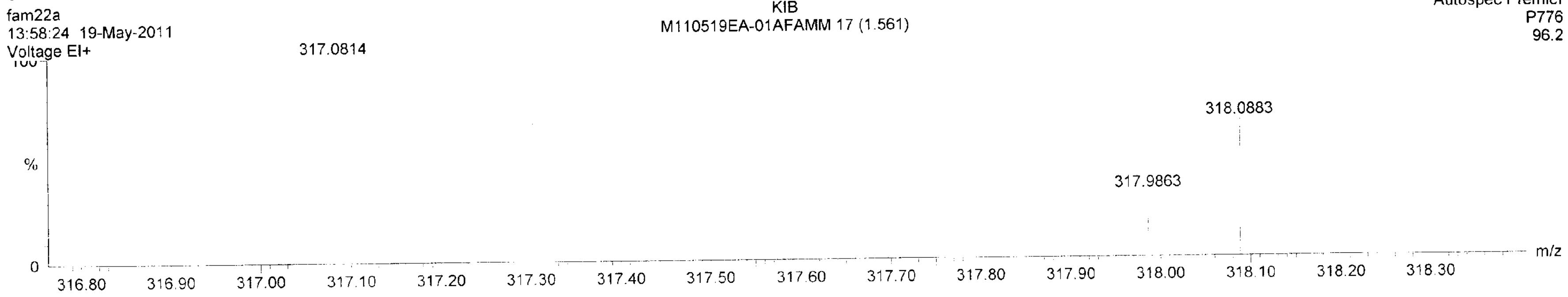


| | | | | | | |
|---|---|---|---|---|---|---|
| Minimum: | | | | 0.5 | | |
| Maximum: | | 100.0 | 10.0 | 40.0 | | |
| Mass | Calc. Mass | mDa | PPM | DBE | i-FIT | Formula |
| 318.0883 | 318.0892 | -0.9 | -2.8 | 14.0 | 5546046.5 | C20 H14 O4 |

Figure S28. $^{1}$H NMR Spectrum of Compound **10**.

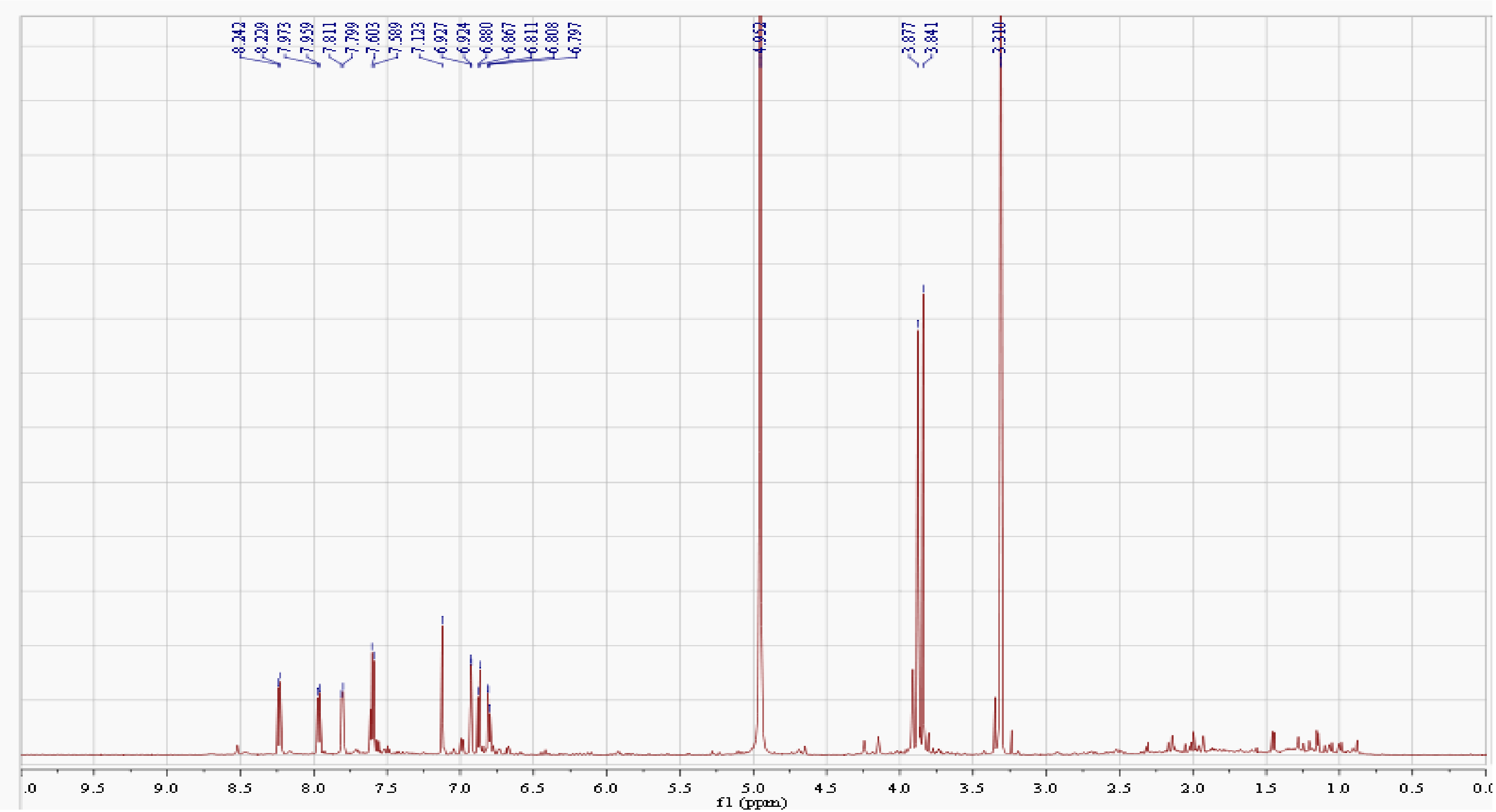

Figure S29. $^{13}$C NMR Spectrum of Compound **10**.

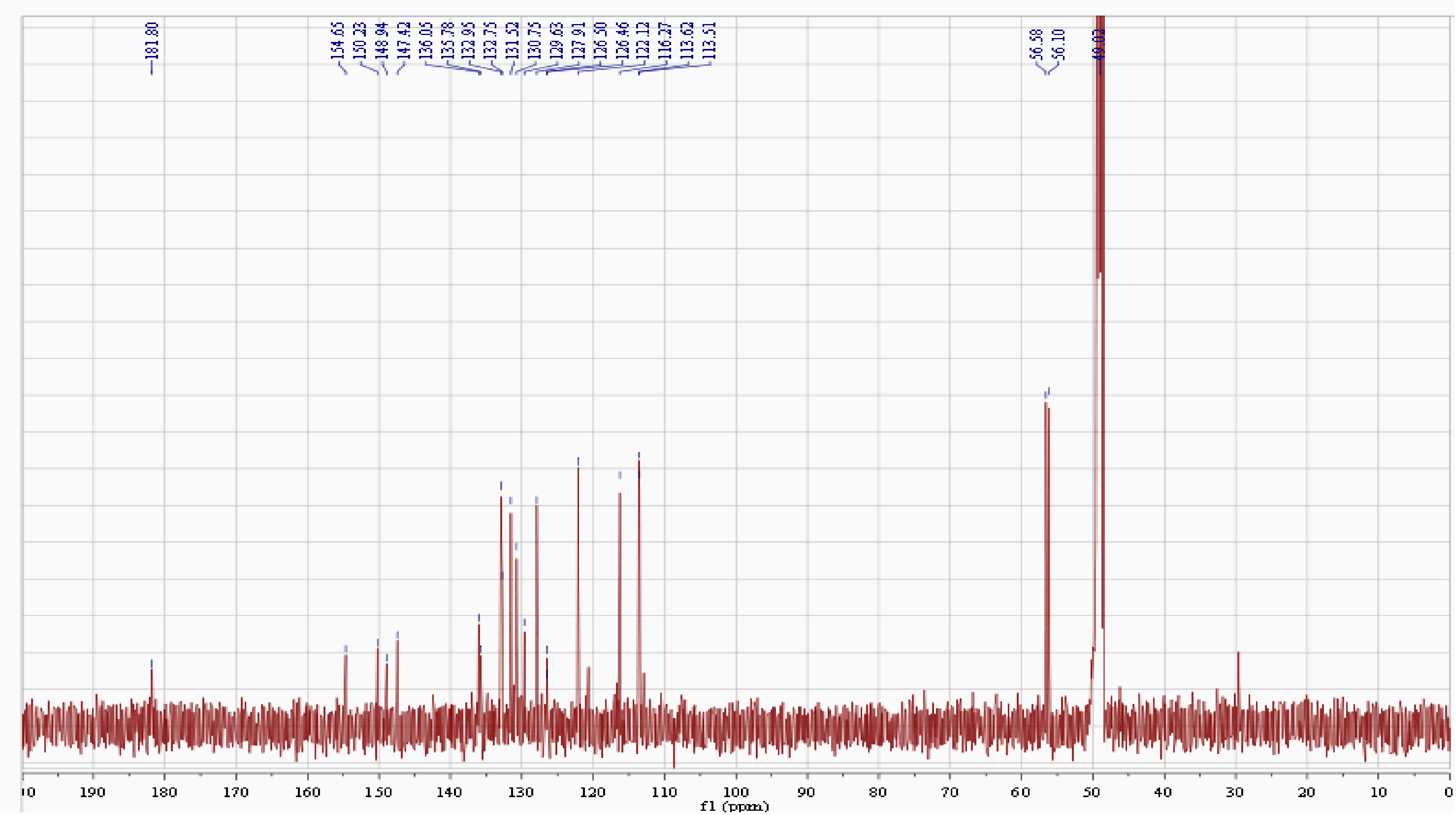

Figure S30. HREIMS Spectrum of Compound **10**.

## Elemental Composition Report

**Single Mass Analysis (displaying only valid results)**
Tolerance = 10.0 PPM / DBE: min = 0.5, max = 40.0
Selected filters: None

Monoisotopic Mass, Odd and Even Electron Ions
26 formula(e) evaluated with 1 results within limits (up to 51 closest results for each mass)
Elements Used:
C: 0-200 H: 0-400 O: 0-5

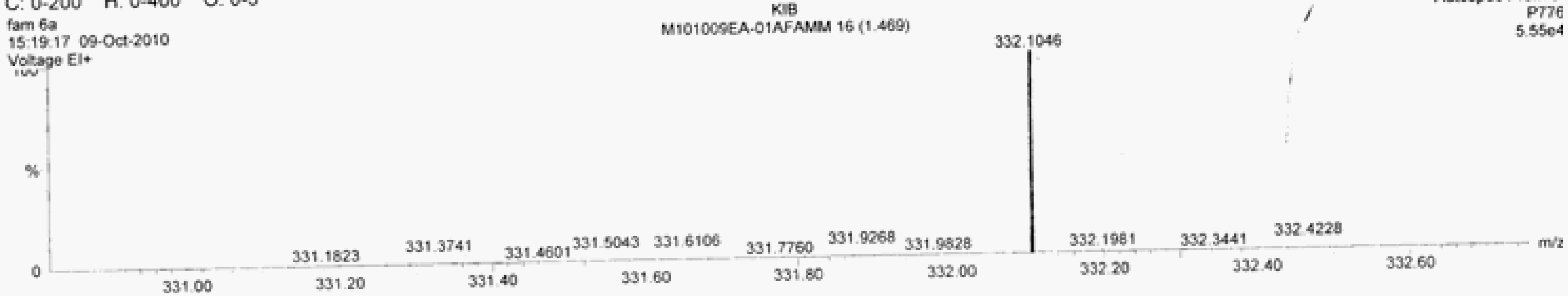


| | | | | | | |
|---|---|---|---|---|---|---|
| Minimum: | | | | 0.5 | | |
| Maximum: | | 100.0 | 10.0 | 40.0 | | |
| Mass | Calc. Mass | mDa | PPM | DBE | i-FIT | Formula |
| 332.1046 | 332.1049 | -0.3 | -0.9 | 14.0 | 5573777.0 | C21 H16 O4 |

Figure S31. $^{1}$H NMR Spectrum of Compound **13**.

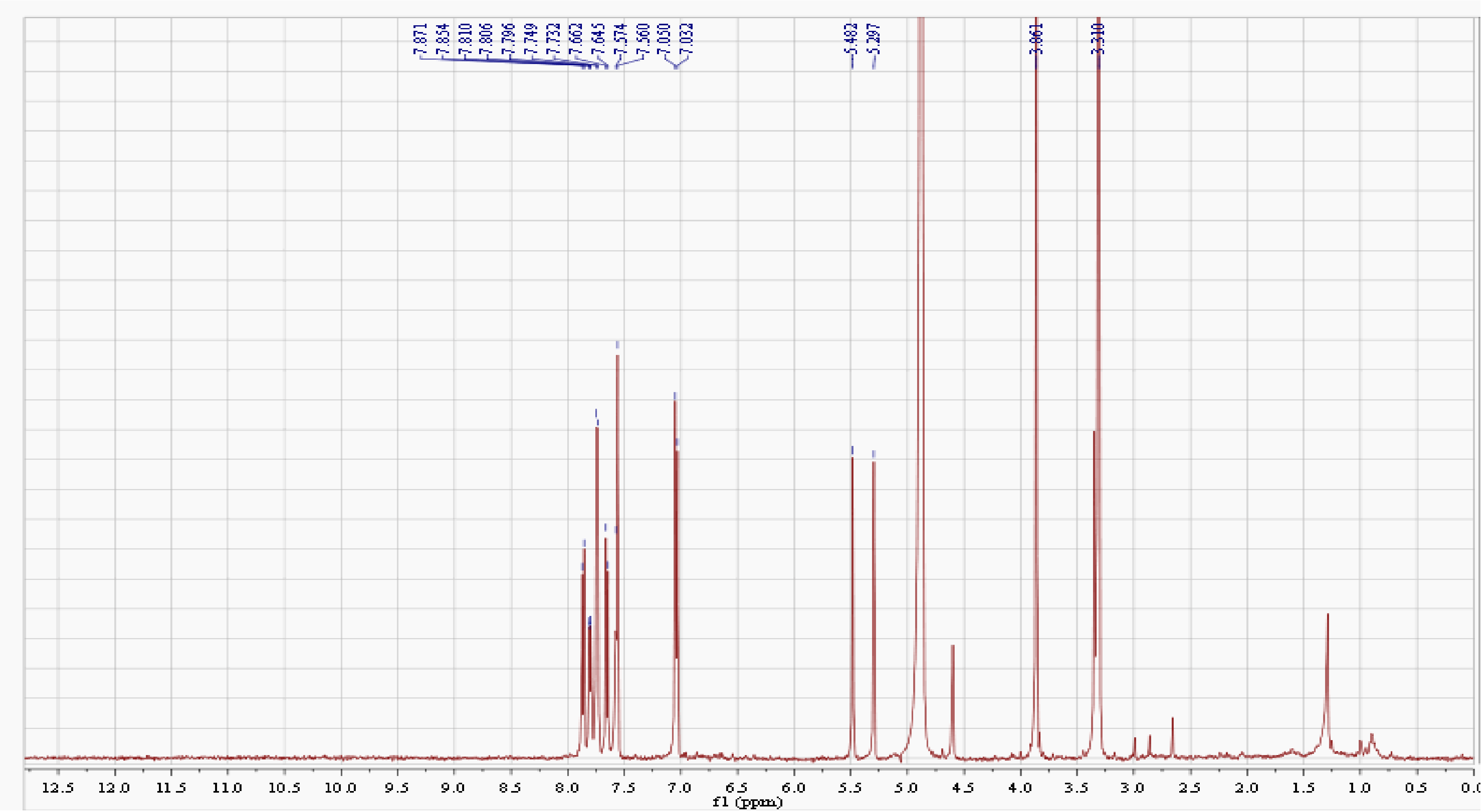

Figure S32. $^{13}$C NMR Spectrum of Compound **13**.

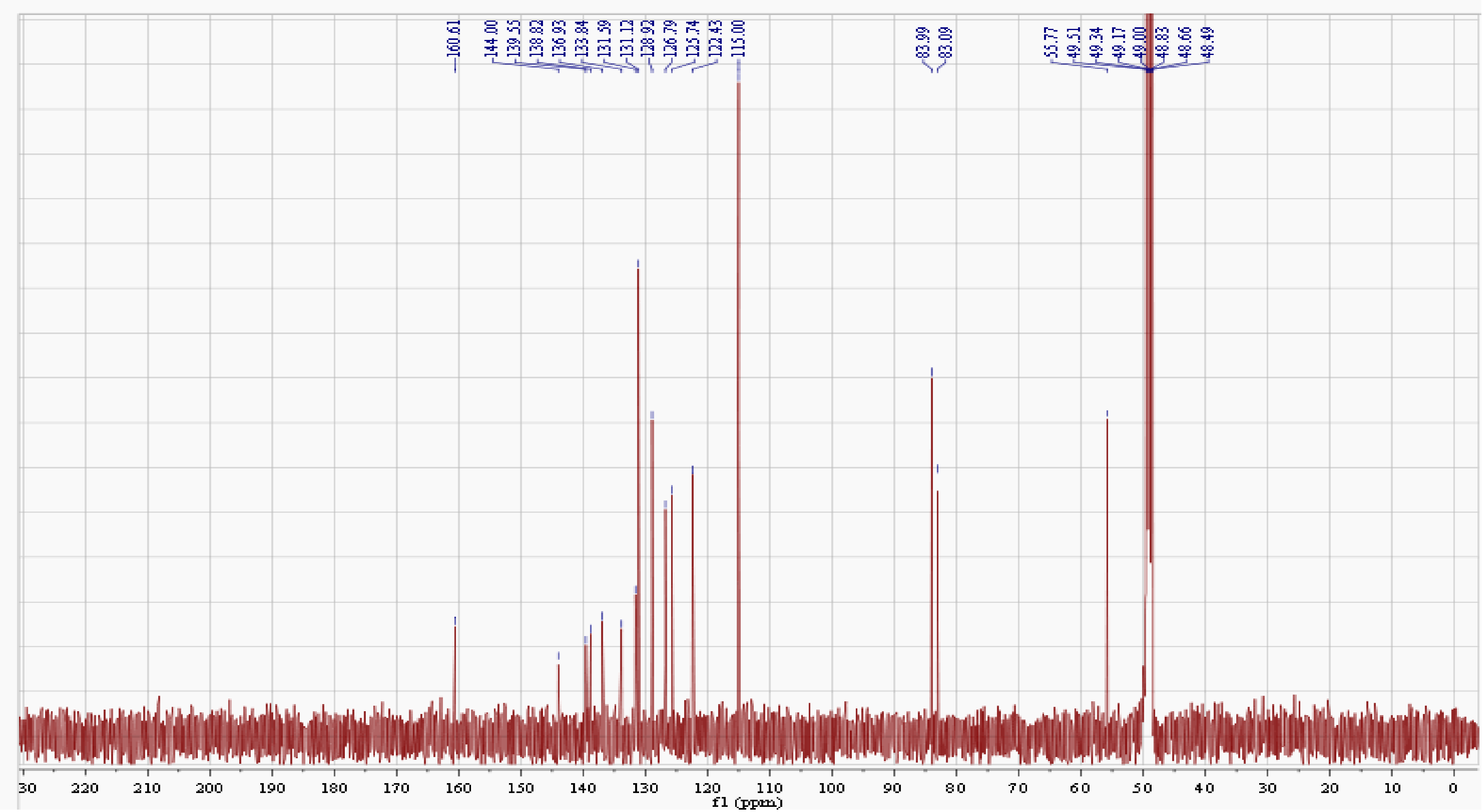

Figure S33. $^{13}$C DEPT 135 Spectrum of Compound **13**.

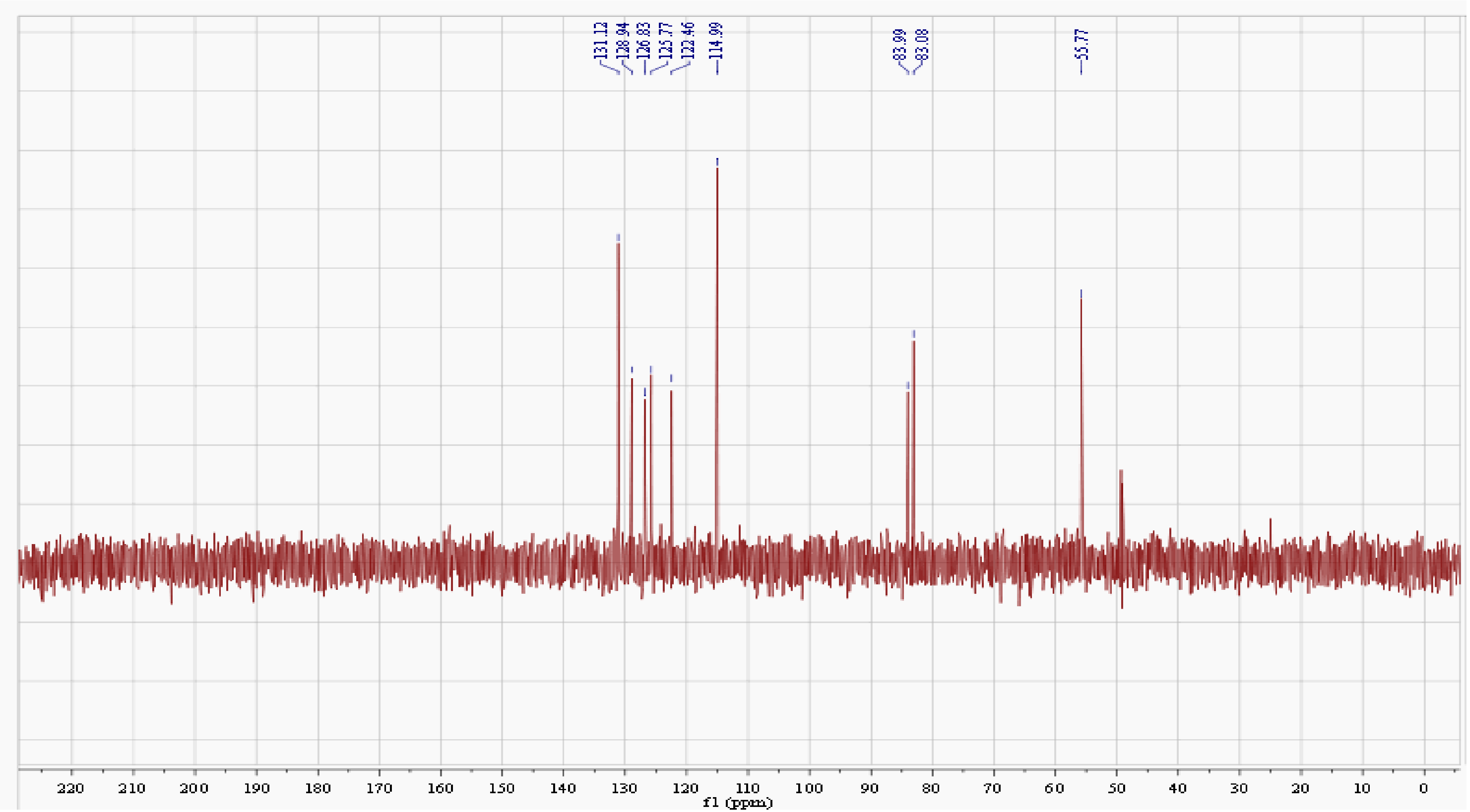

Figure S34. HSQC Spectrum of Compound **13**.

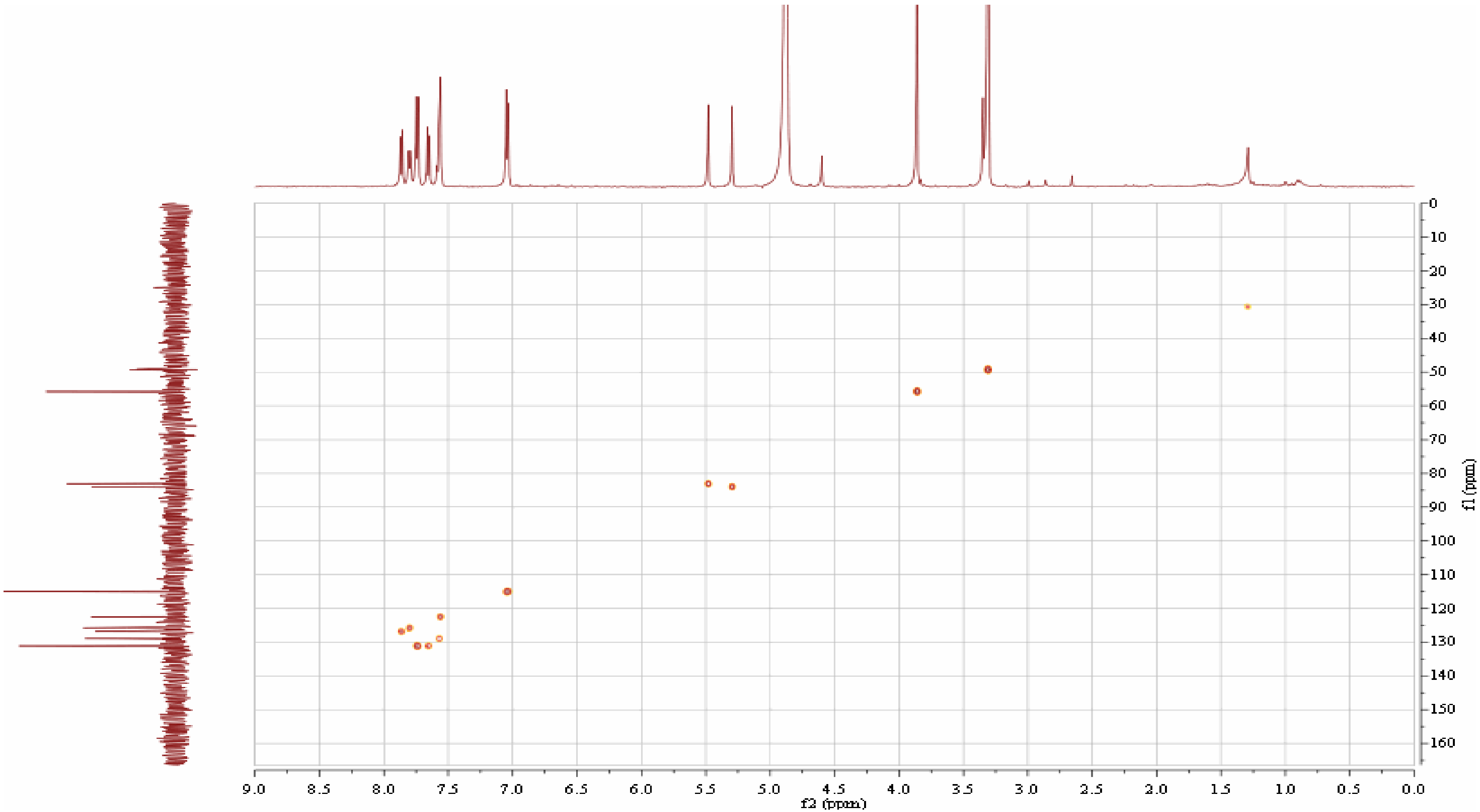

Figure S35. COSY Spectrum of Compound **13**.

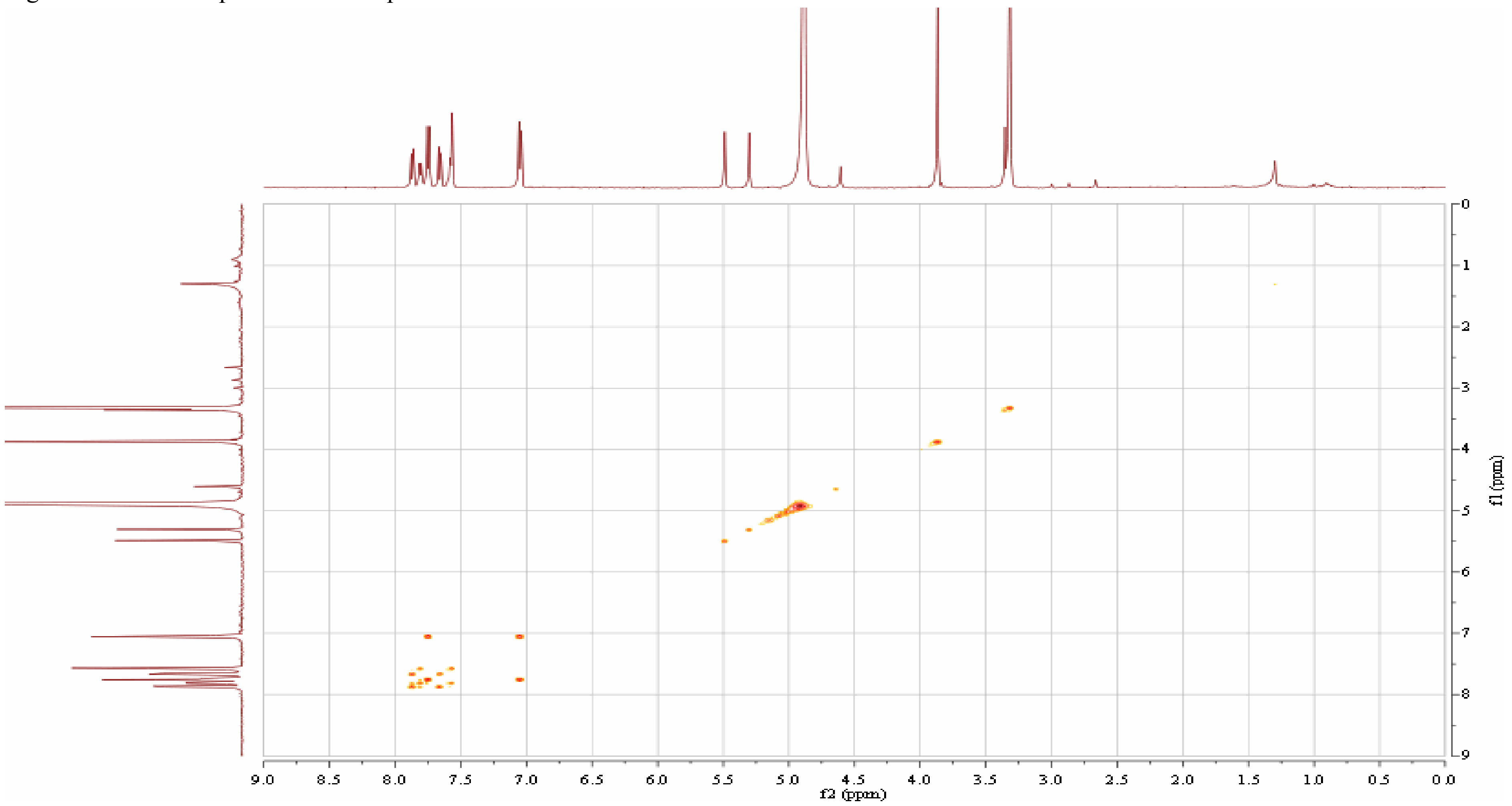

Figure S36. HMBC Spectrum of Compound **13**.

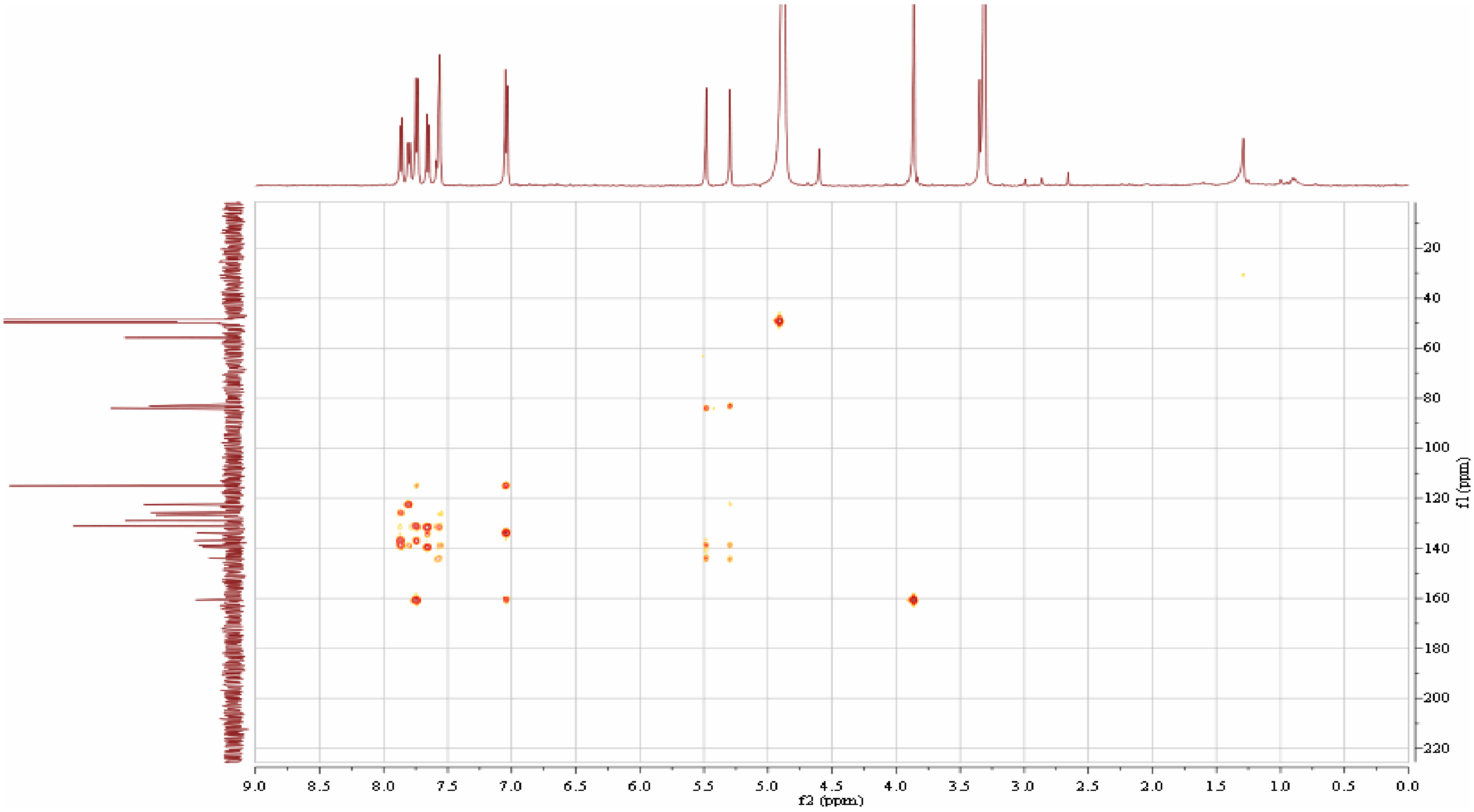

Figure S37. ROESY Spectrum of Compound **13**.

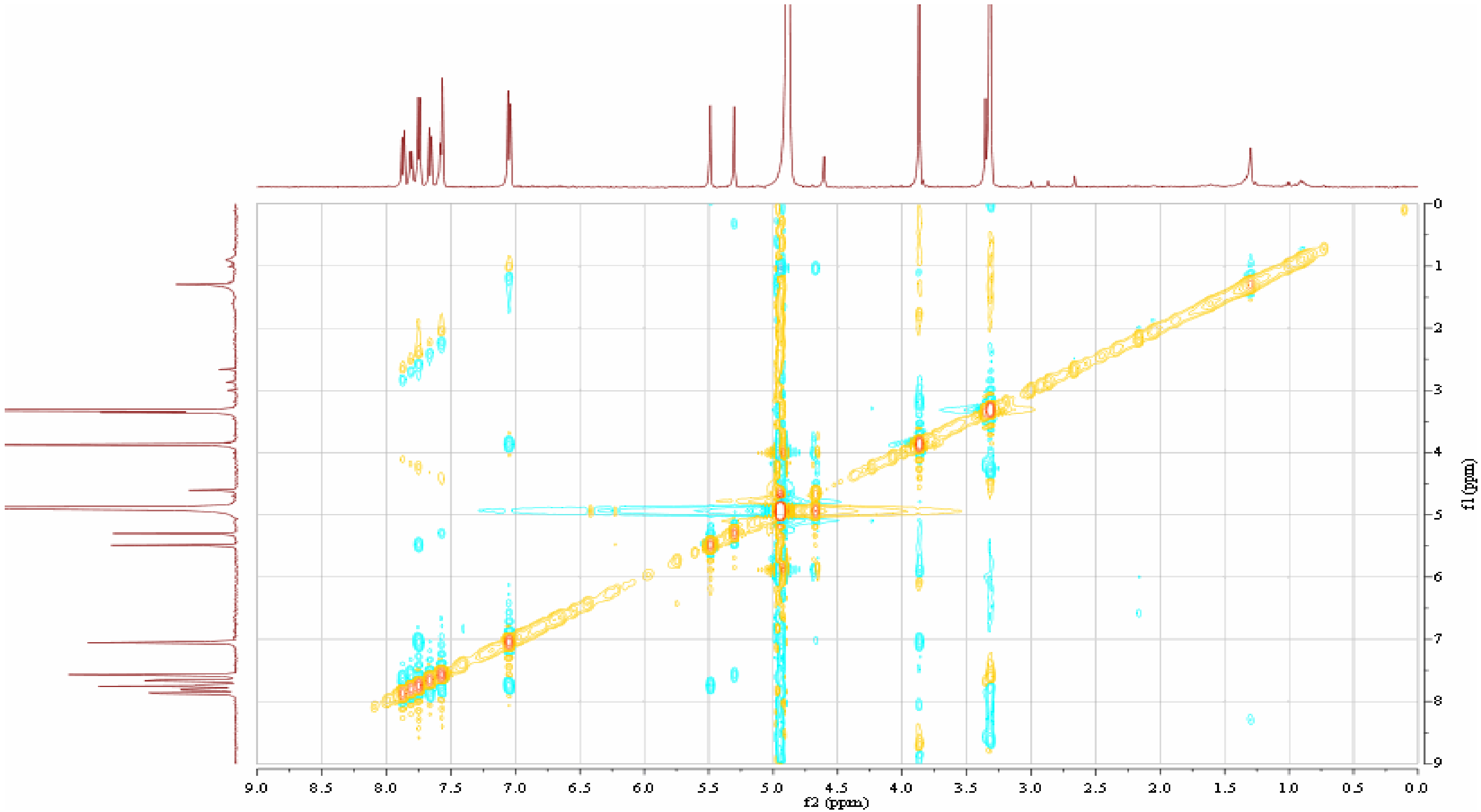

Figure S38. HRESIMS Spectrum of Compound **13**.

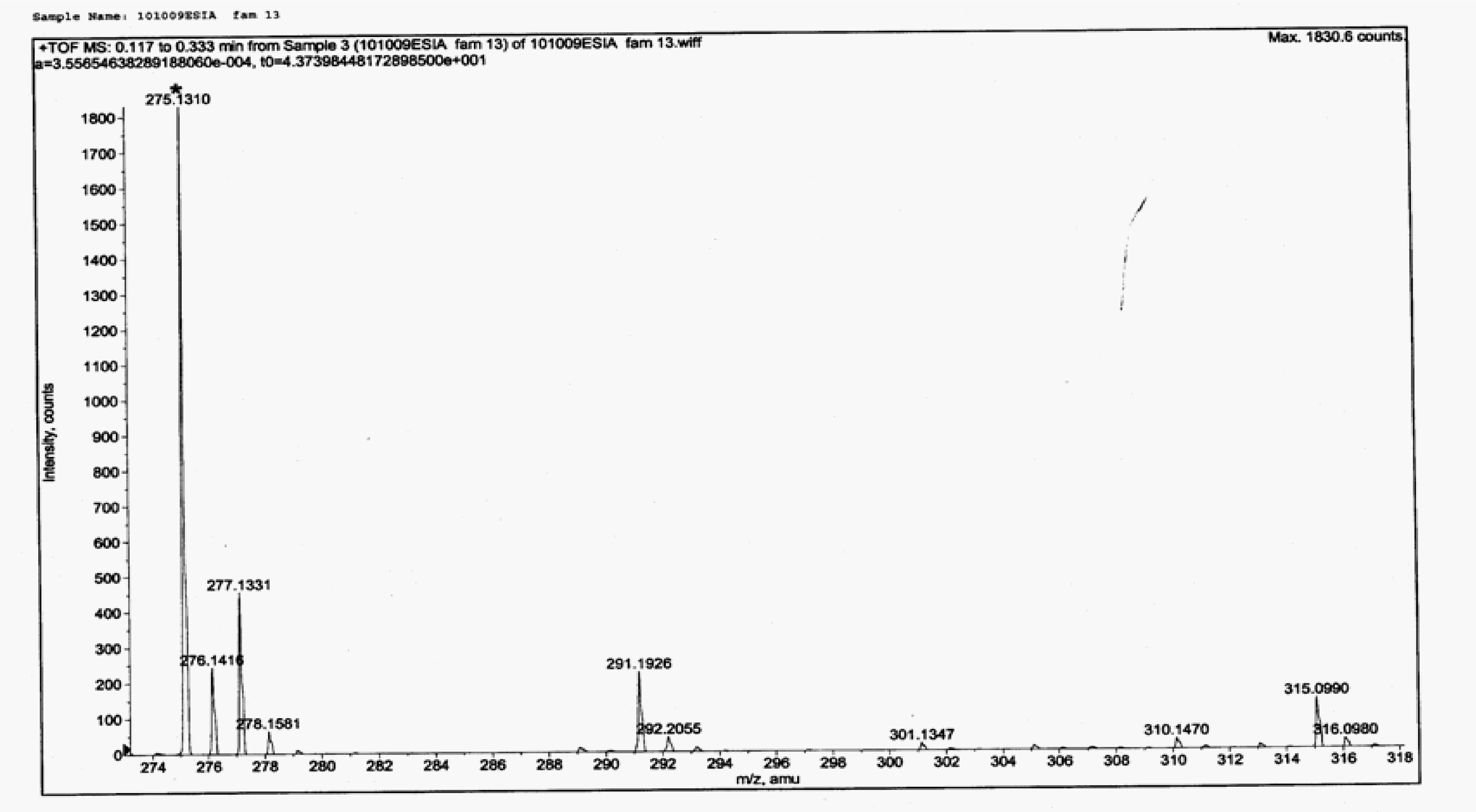

Figure S39. $^{1}$H NMR Spectrum of Compound **16**.

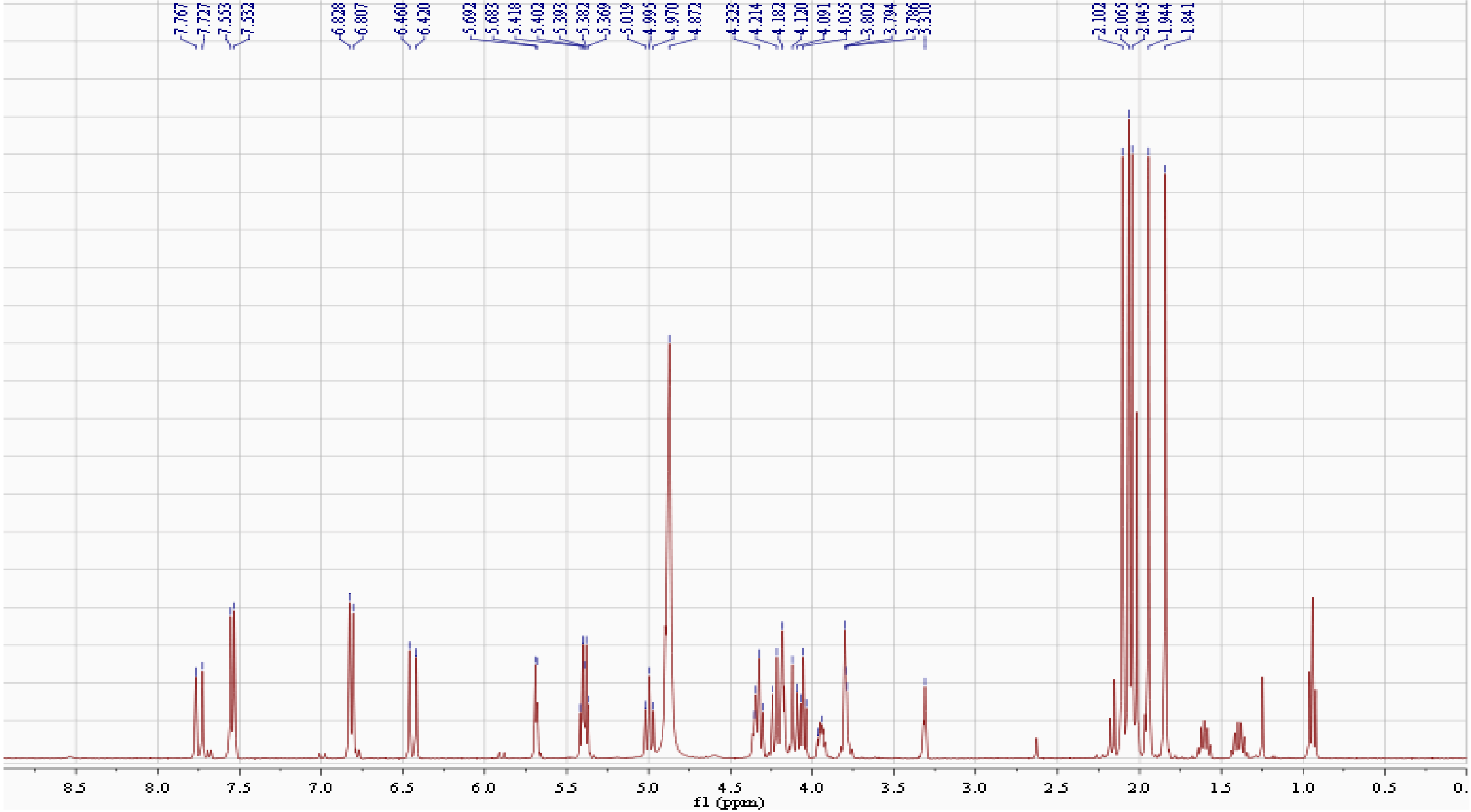

Figure S40. $^{13}$C NMR Spectrum of Compound **16**.

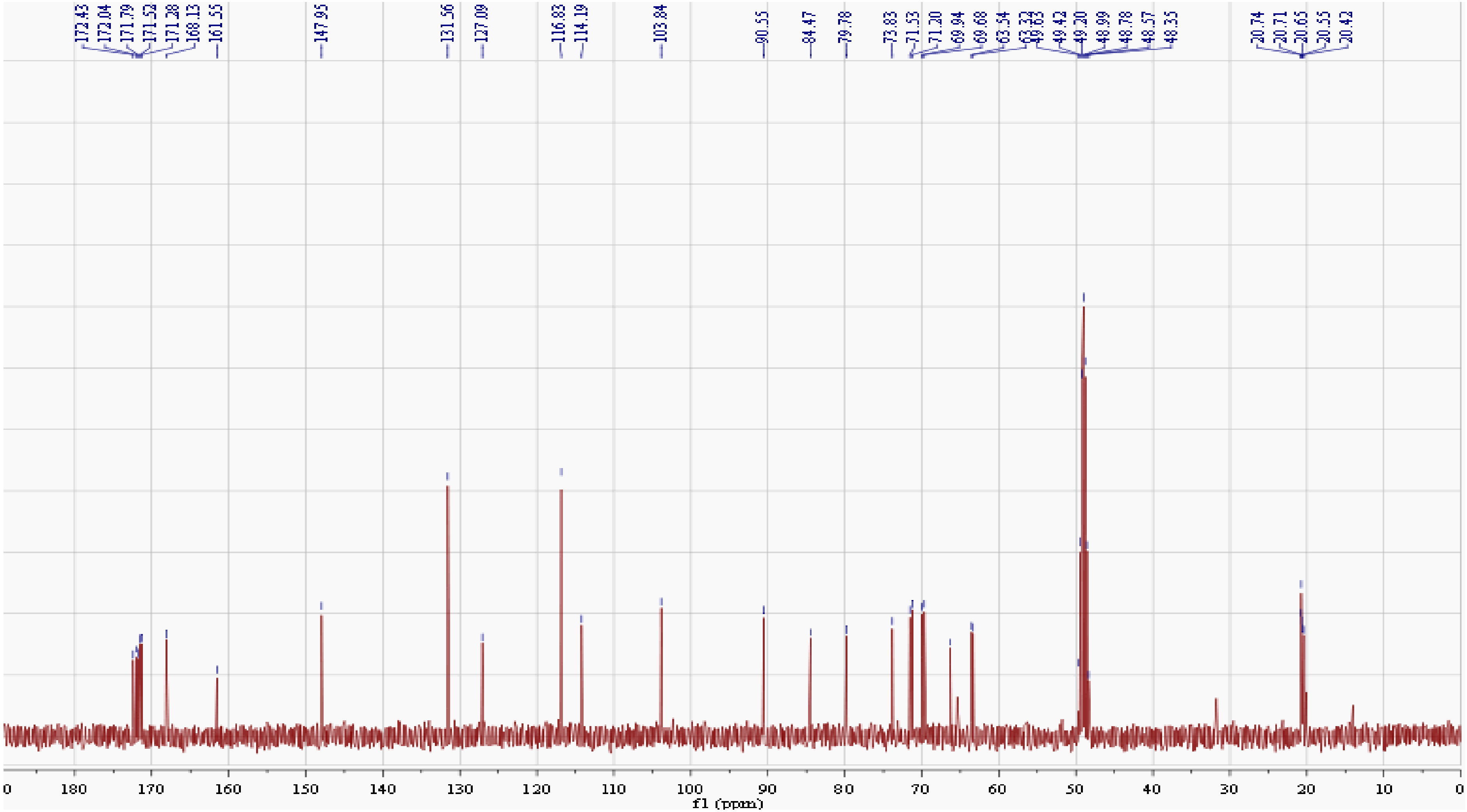

Figure S41. $^{13}$C DEPT 135 Spectrum of Compound **16**.

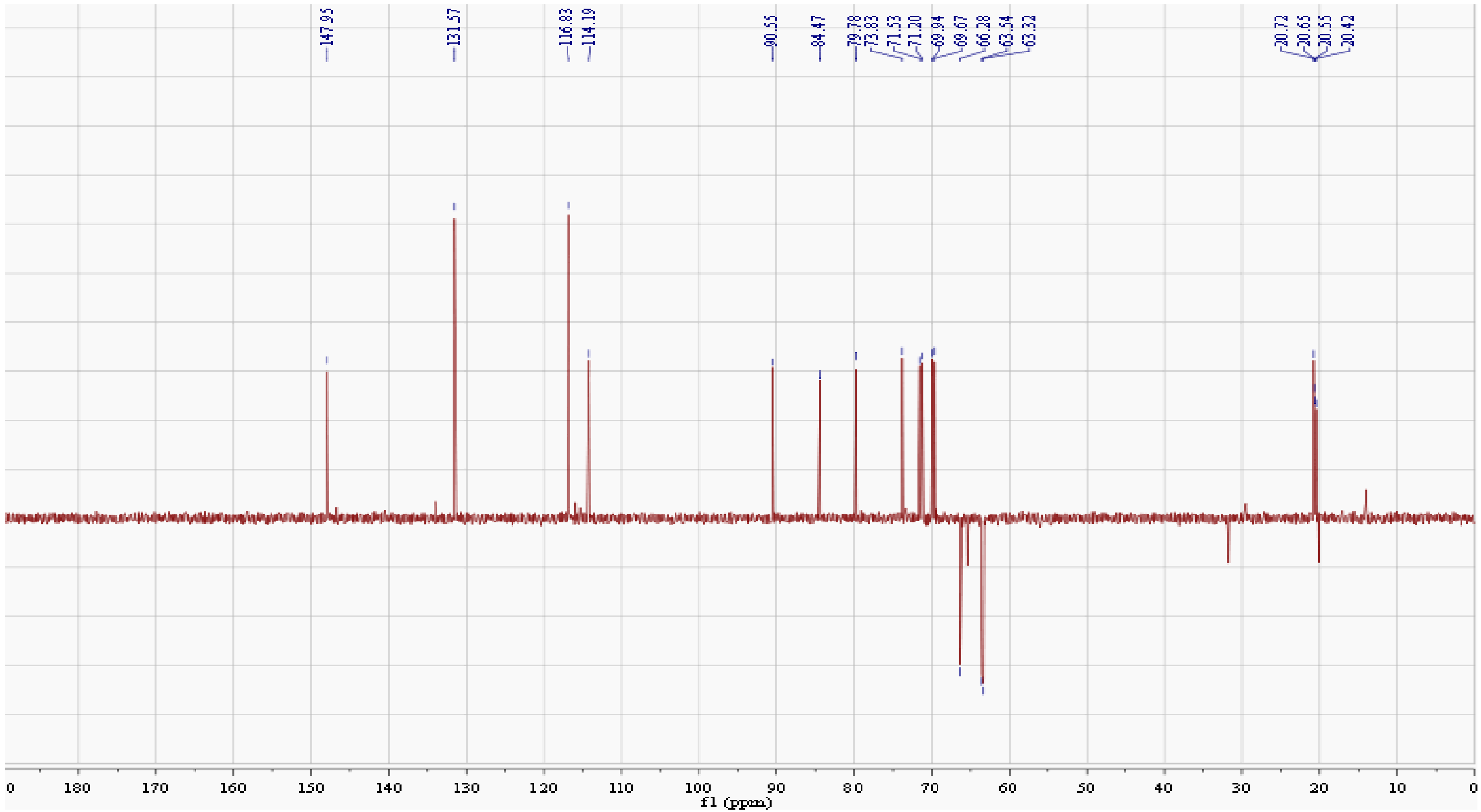

Figure S42. HSQC Spectrum of Compound **16**.

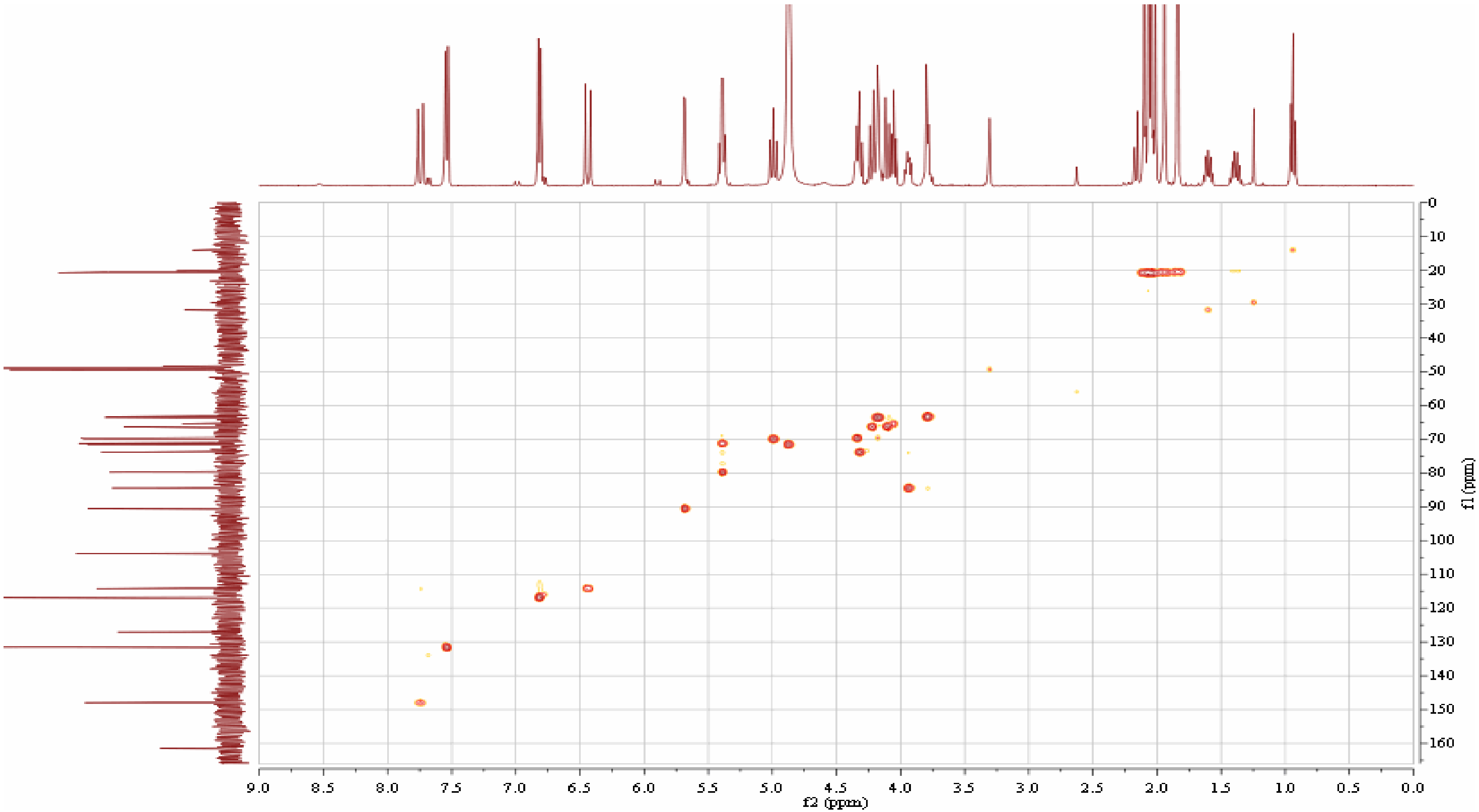

Figure S43. COSY Spectrum of Compound **16**.

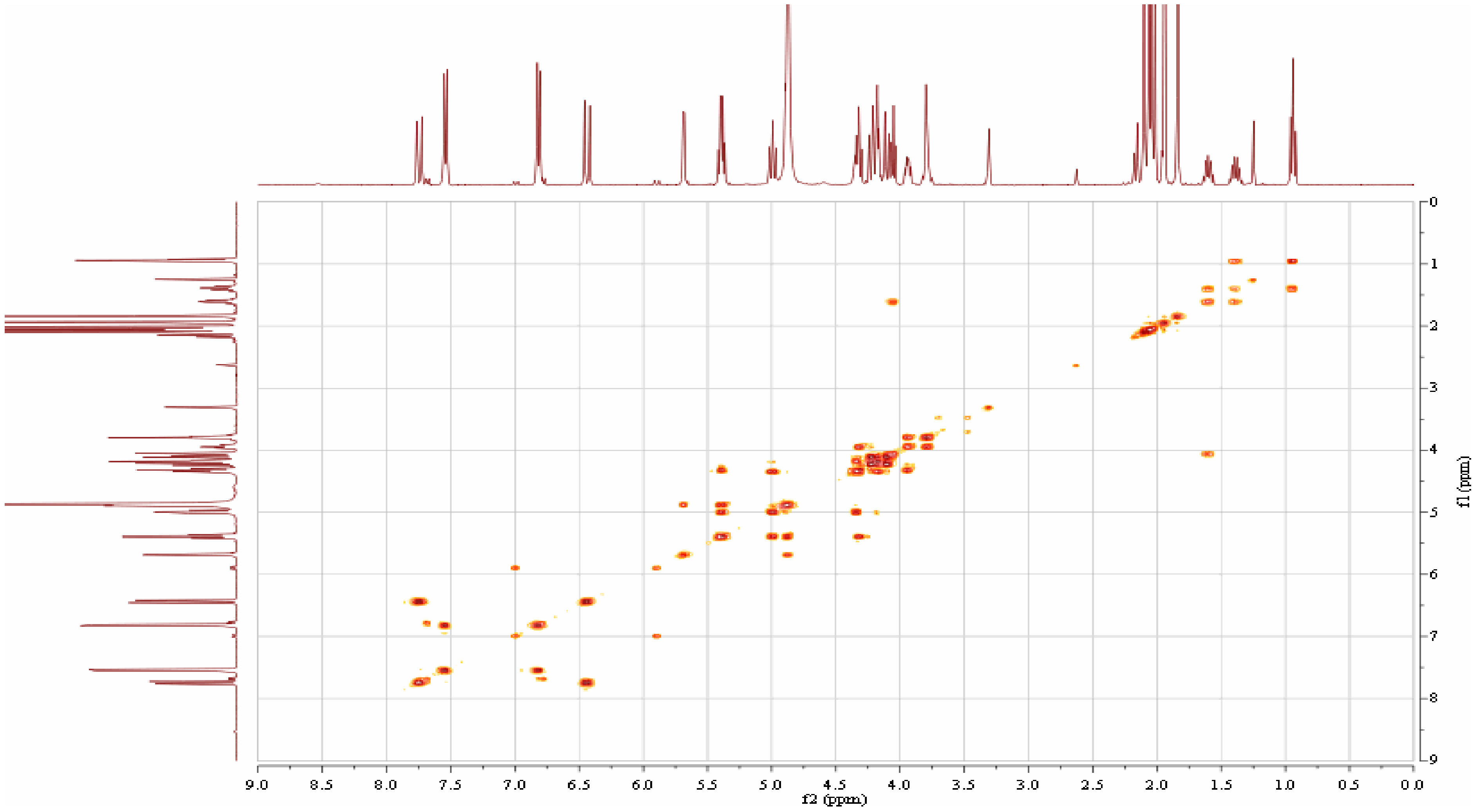

Figure S44. HMBC Spectrum of Compound **16**.

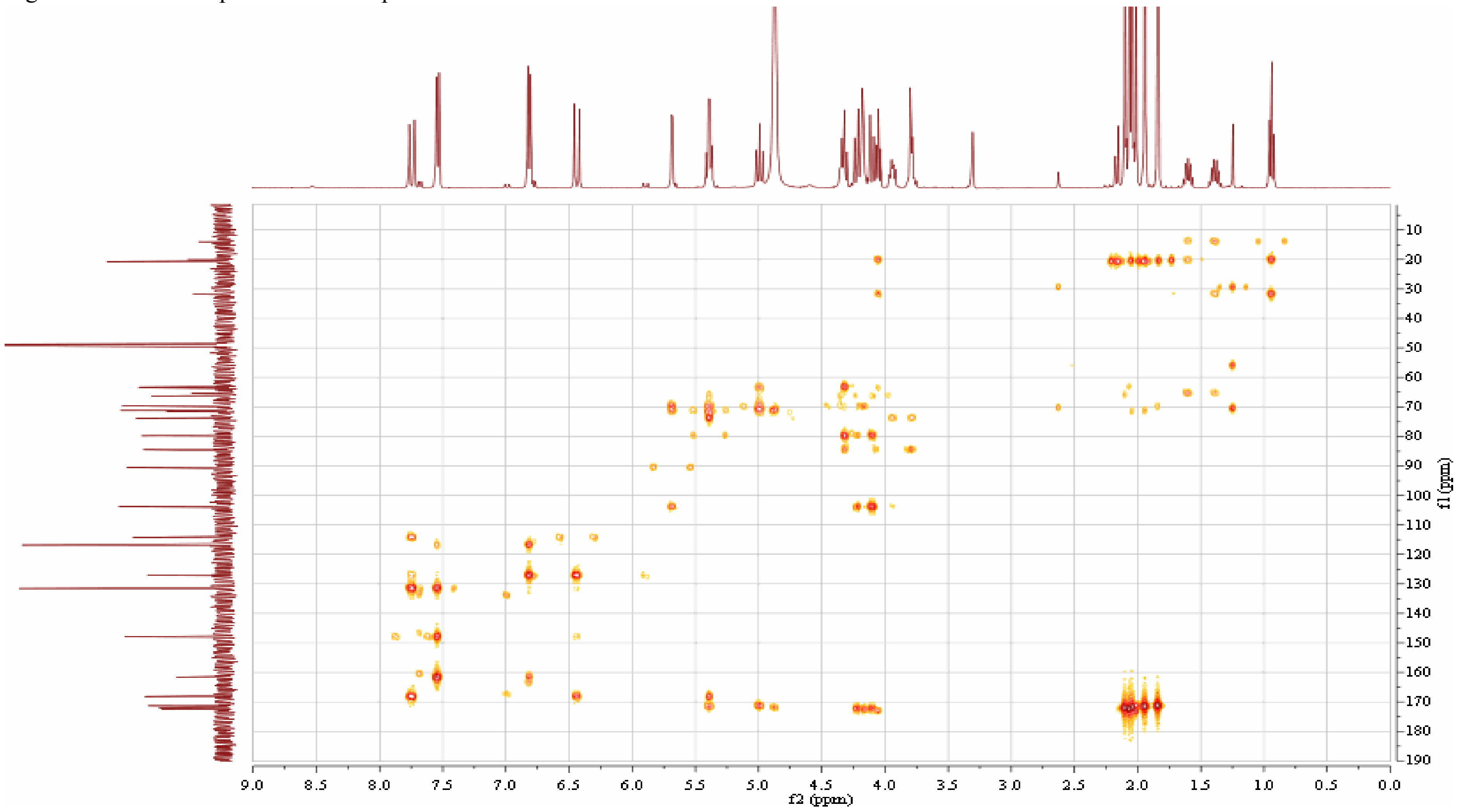

Figure S45. ROESY Spectrum of Compound **16**.

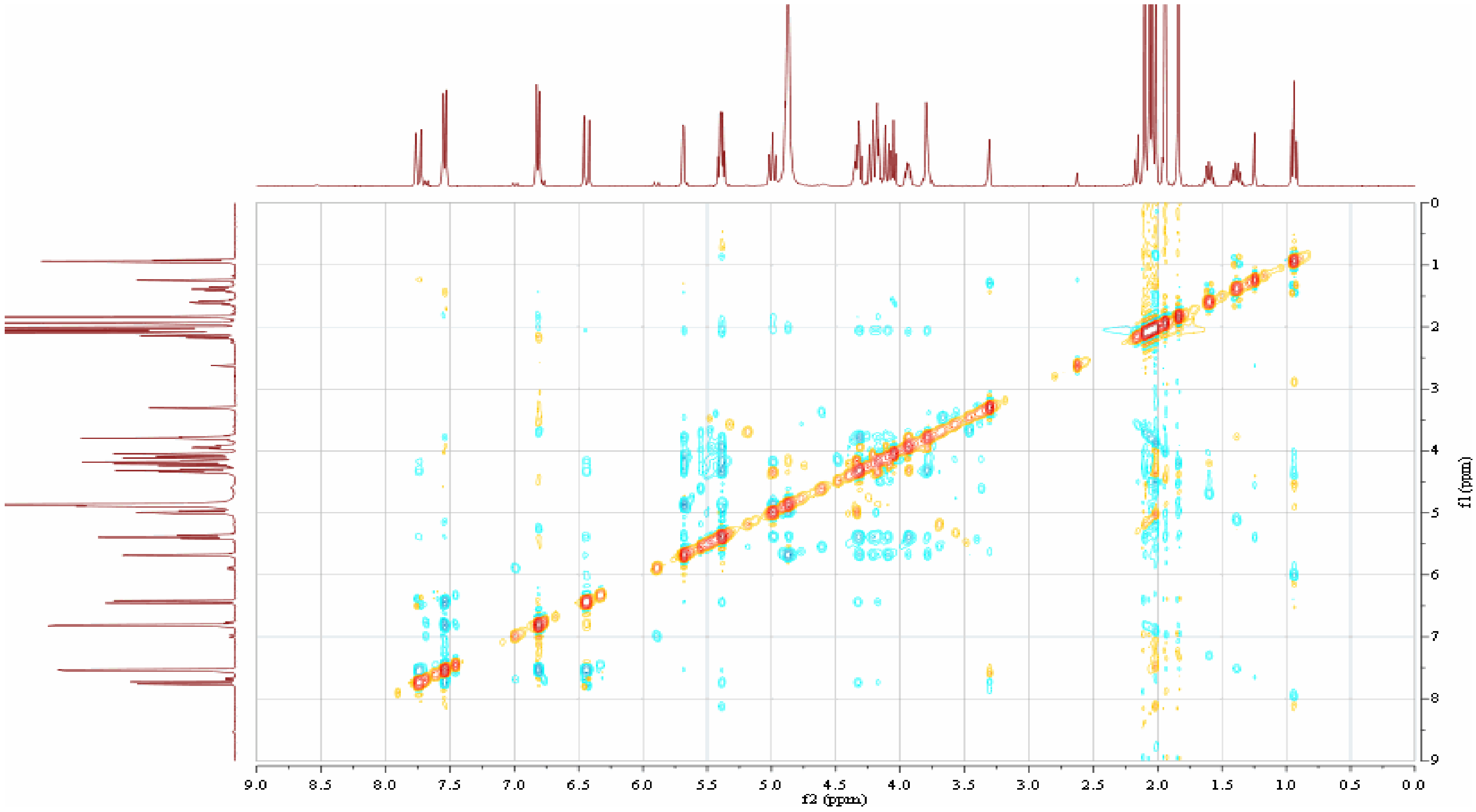

Figure S46. HRESIMS Spectrum of Compound **16**.

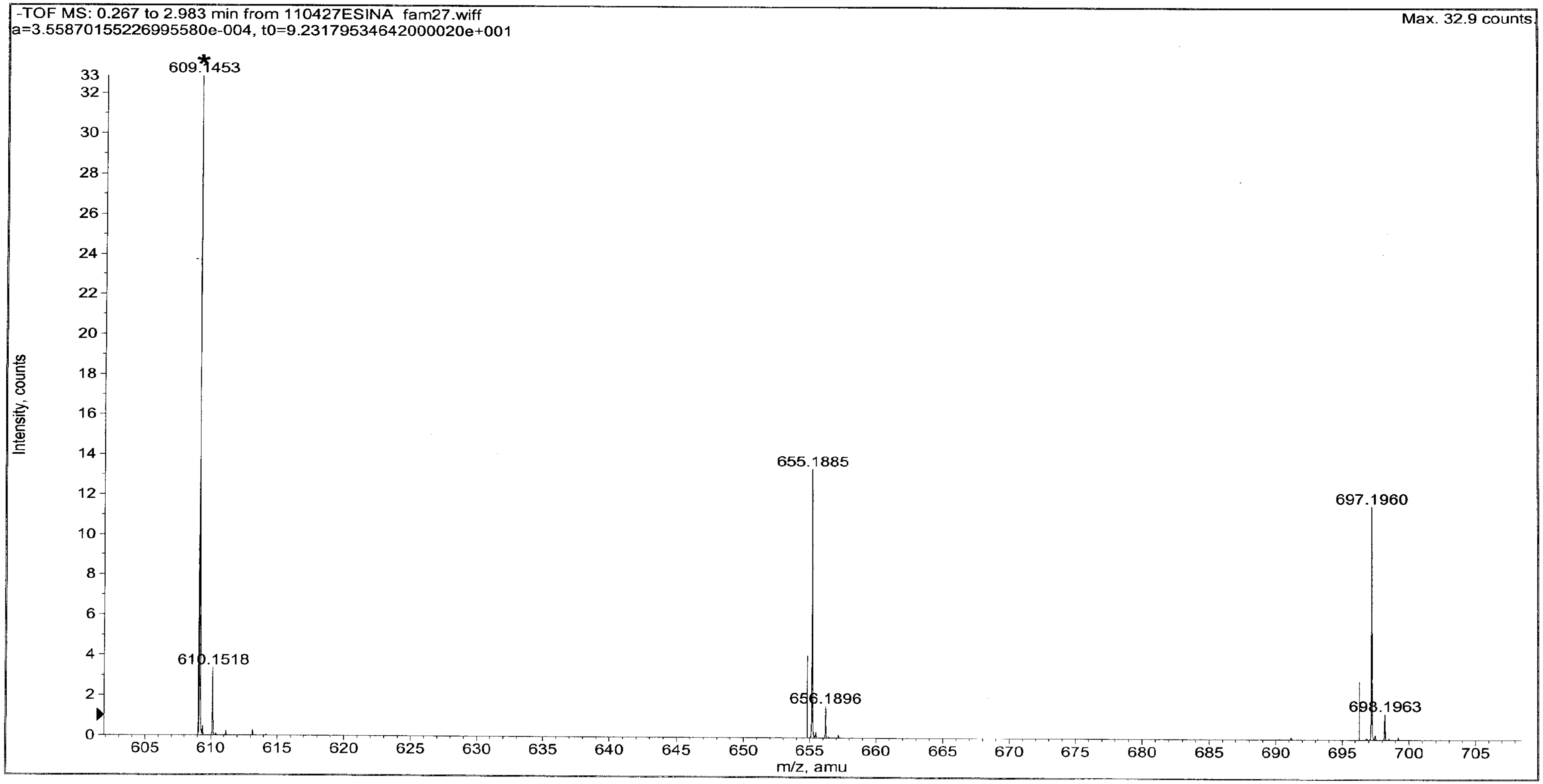

Figure S47. $^{1}$H NMR Spectrum of Compound **17**.

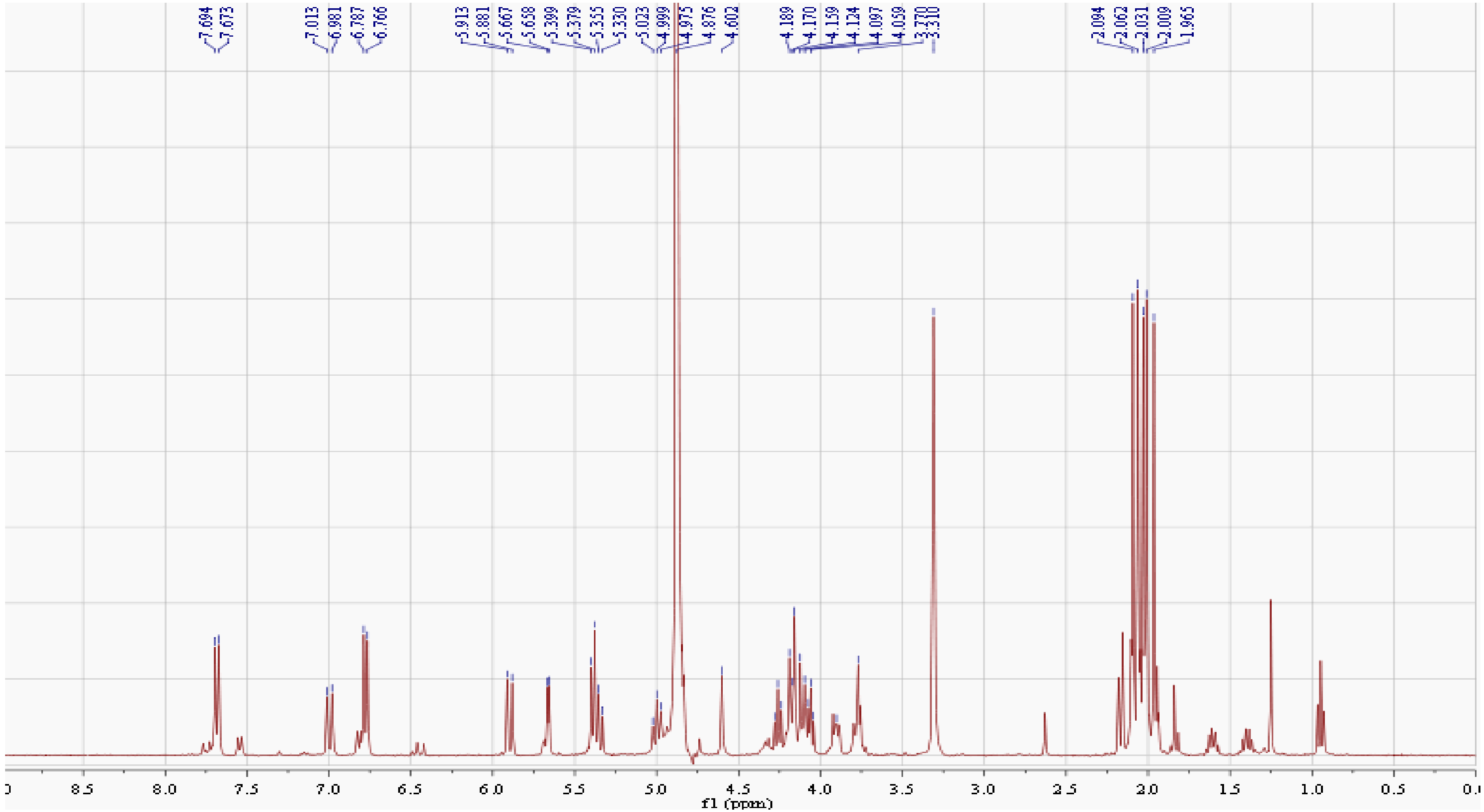

Figure S48. $^{13}C$ NMR Spectrum of Compound **17**.

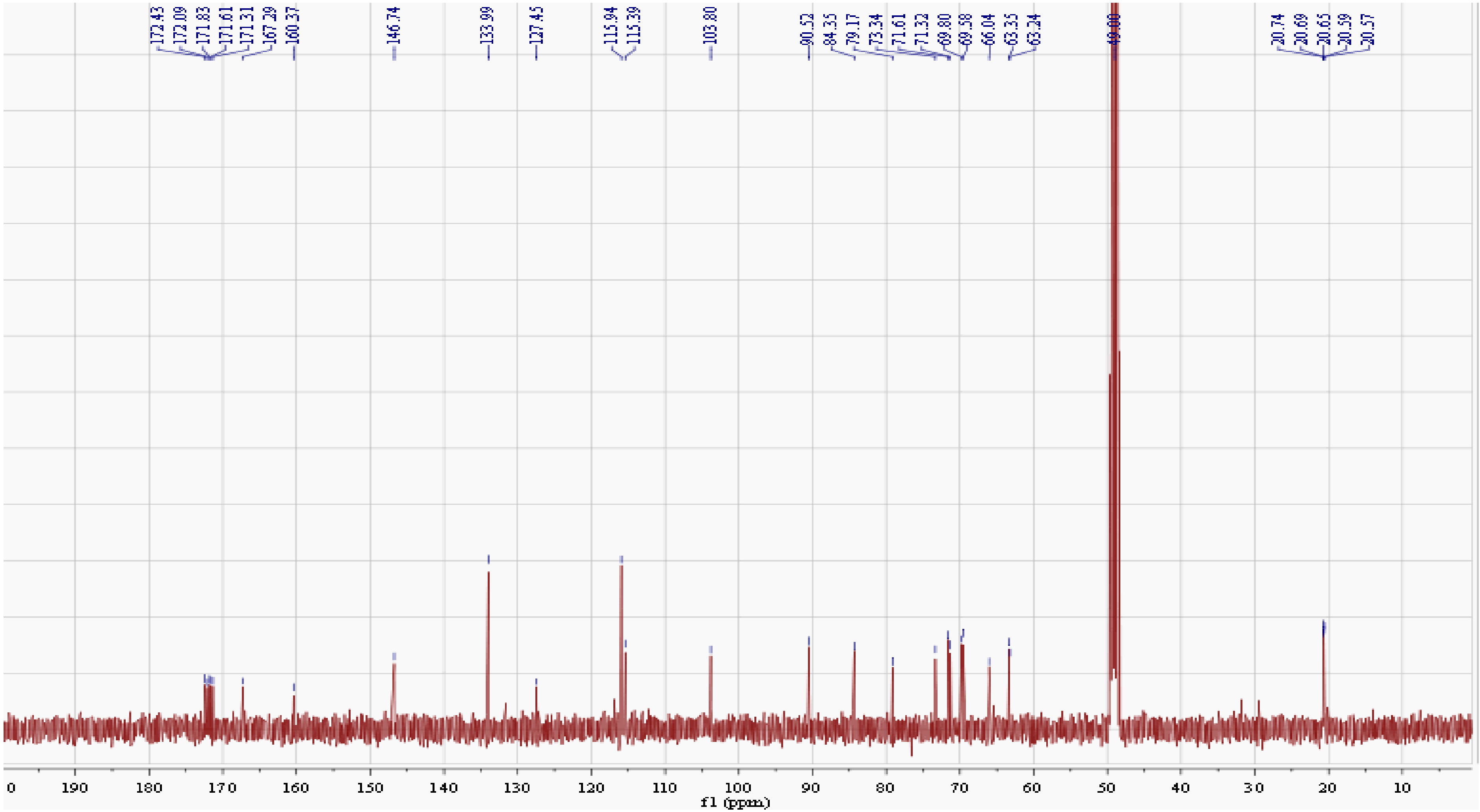

Figure S49. HRESIMS Spectrum of Compound **17**.

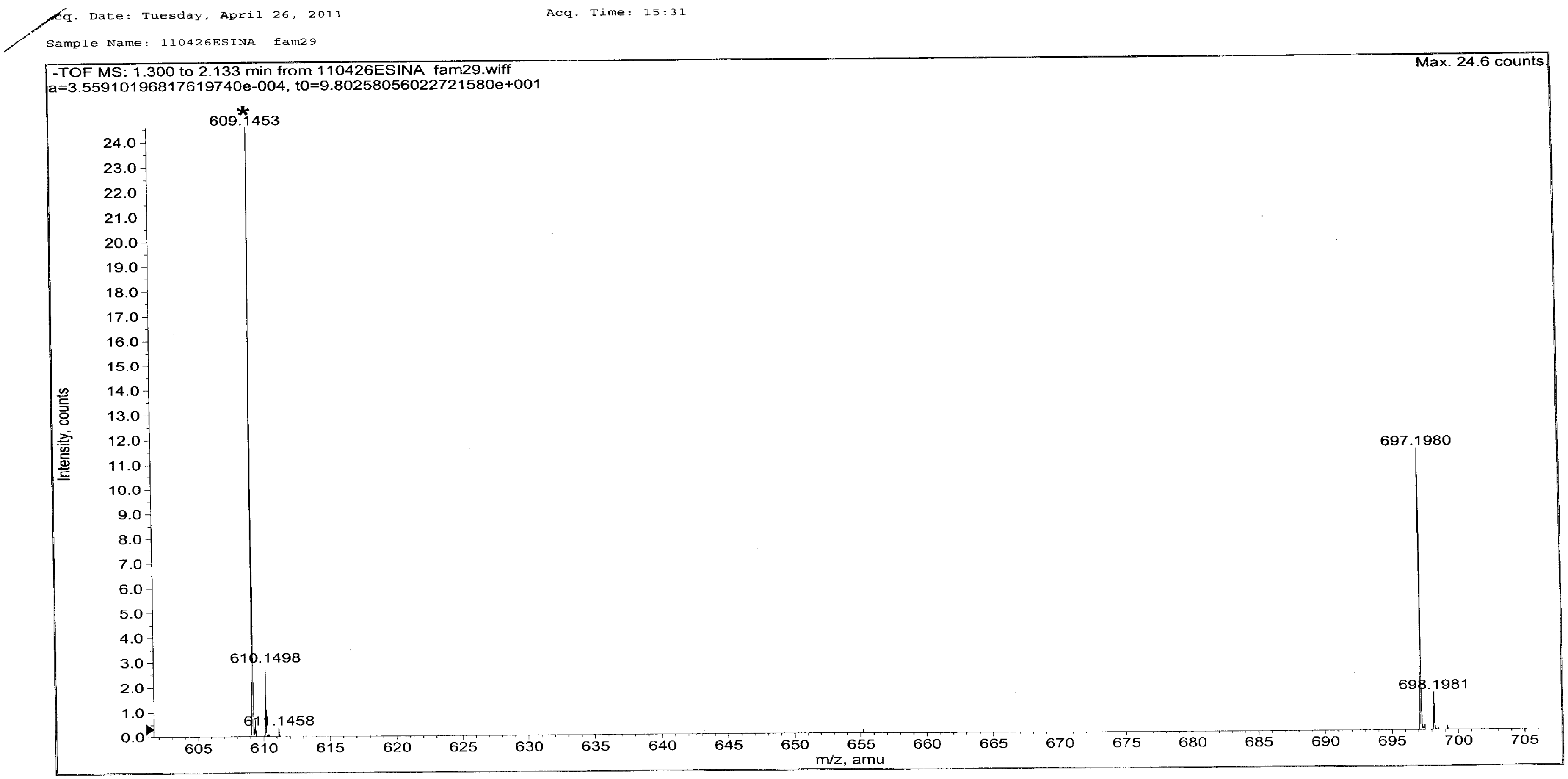

Figure S50. ESIMS Spectrum of sucrose.

# Mass Spectrum List Report

**Analysis Info**

| | | | |
|---|---|---|---|
| Analysis Name | D:\DATA\2011file\110513\fam 27T3000.d | Acquisition Date | 5/13/2011 9:28:38 AM |
| Method | ms_ptservice.m | Operator | QUYAN |
| Sample Name | xl-1-2 | Instrument | HCT |
| Comment | balance method | | |

**Acquisition Parameter**

| | | | | | |
|---|---|---|---|---|---|
| Ion Source Type | ESI | Ion Polarity | Negative | Alternating Ion Polarity | off |
| Mass Range Mode | Std/Enhanced | Scan Begin | 100 m/z | Scan End | 1000 m/z |
| Capillary Exit | -100.0 Volt | Skimmer | -40.0 Volt | Trap Drive | 73.9 |
| Accumulation Time | 2000 µs | Averages | 7 Spectra | Auto MS/MS | off |

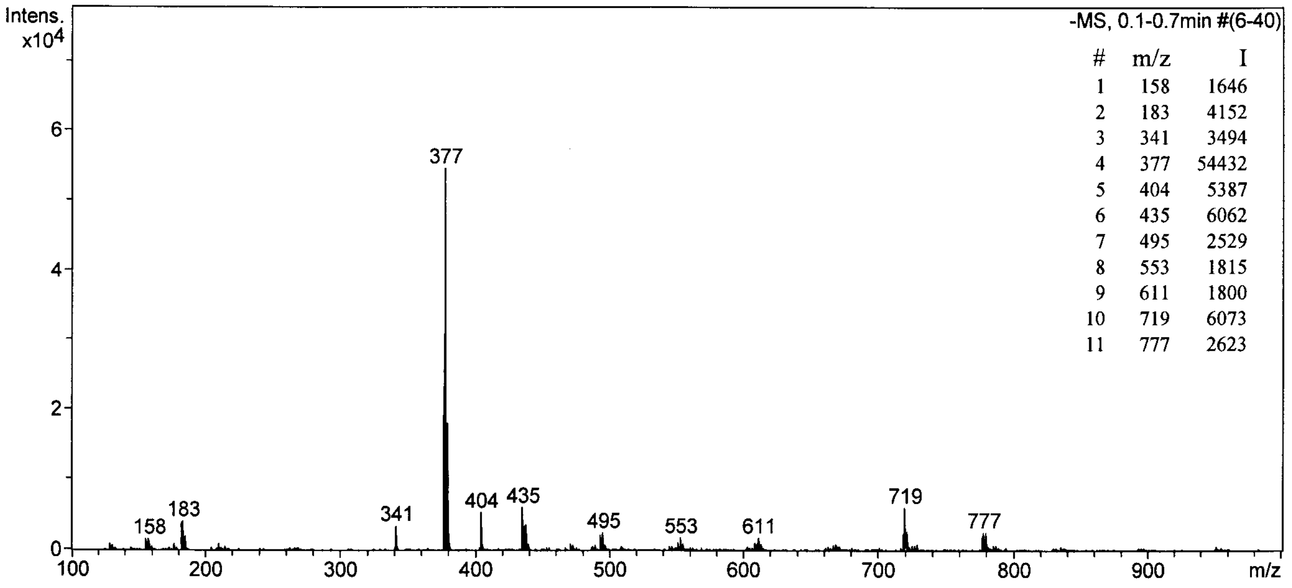

Supplement: Supplementary file 1 — Supplementary material, approximately 1.23 MB. [file 13659_2011_7_MOESM1_ESM.pdf]
